# RNA m^6^A modification orchestrates a LINE-1–host interaction that facilitates retrotransposition and contributes to long gene vulnerability

**DOI:** 10.1038/s41422-021-00515-8

**Published:** 2021-06-09

**Authors:** Feng Xiong, Ruoyu Wang, Joo-Hyung Lee, Shenglan Li, Shin-Fu Chen, Zian Liao, Lana Al Hasani, Phuoc T. Nguyen, Xiaoyu Zhu, Joanna Krakowiak, Dung-Fang Lee, Leng Han, Kuang-Lei Tsai, Ying Liu, Wenbo Li

**Affiliations:** 1 Department of Biochemistry and Molecular Biology, McGovern Medical School, University of Texas Health Science Center, Houston, TX USA; 2 Graduate School of Biomedical Sciences, University of Texas MD Anderson Cancer Center and UTHealth, Houston, TX USA; 3 The Vivian L. Smith Department of Neurosurgery, McGovern Medical School, University of Texas Health Science Center, Houston, TX USA; 4 Center for Stem Cell and Regenerative Medicine, The Brown Foundation Institute of Molecular Medicine for the Prevention of Human Diseases, The University of Texas Health Science Center at Houston, Houston, TX USA; 5 Department of Integrative Biology & Pharmacology, McGovern Medical School, University of Texas Health Science Center, Houston, TX USA; 6 Center for Precision Health, School of Biomedical Informatics, The University of Texas Health Science Center at Houston, Houston, TX USA; 7 Center for Epigenetics & Disease Prevention, Institute of Biosciences and Technology, Texas A&M University, Houston, TX USA

**Keywords:** Transcriptional regulatory elements, RNA modification, Neural stem cells

## Abstract

The molecular basis underlying the interaction between retrotransposable elements (RTEs) and the human genome remains poorly understood. Here, we profiled *N*^6^-methyladenosine (m^6^A) deposition on nascent RNAs in human cells by developing a new method MINT-Seq, which revealed that many classes of RTE RNAs, particularly intronic LINE-1s (L1s), are strongly methylated. These m^6^A-marked intronic L1s (MILs) are evolutionarily young, sense-oriented to hosting genes, and are bound by a dozen RNA binding proteins (RBPs) that are putative novel readers of m^6^A-modified RNAs, including a nuclear matrix protein SAFB. Notably, m^6^A positively controls the expression of both autonomous L1s and co-transcribed L1 relics, promoting L1 retrotransposition. We showed that MILs preferentially reside in long genes with critical roles in DNA damage repair and sometimes in L1 suppression per se, where they act as transcriptional “roadblocks” to impede the hosting gene expression, revealing a novel host-weakening strategy by the L1s. In counteraction, the host uses the SAFB reader complex to bind m^6^A-L1s to reduce their levels, and to safeguard hosting gene transcription. Remarkably, our analysis identified thousands of MILs in multiple human fetal tissues, enlisting them as a novel category of cell-type-specific regulatory elements that often compromise transcription of long genes and confer their vulnerability in neurodevelopmental disorders. We propose that this m^6^A-orchestrated L1–host interaction plays widespread roles in gene regulation, genome integrity, human development and diseases.

## Introduction

Retrotransposable elements (RTEs), consisting of Long Interspersed Elements (LINEs), Short Interspersed Element (SINEs), and endogenous retroviruses (ERVs), make up nearly half of the mammalian genomes.^1,2^ RTEs are major evolutionary parasites in the mammalian genomes that continuously develop new means to propagate, which on one hand threatens the stability of the host genome, but on the other can also drive new genome evolution.^3–9^ In humans, LINE-1 (or L1) is the most dominant RTEs in terms of the genome sizes they occupy (~17%–21%),^10,11^ and is the only active category capable of autonomous retrotransposition.^6,12,13^ To safeguard the genome integrity, the host employs a series of epigenetic strategies to suppress the transcription of L1 and other RTEs, e.g. DNA methylation^14^ and histone methylation (e.g. H3K9me3).^15,16^ Post-transcriptional mechanisms were also involved in RTE suppression.^17,18^ Recently, DNA damage repair (DDR) and replication related factors were identified as new suppressors of L1 activity.^11,19,20^ However, as compared to these defense systems deployed by the hosts to suppress RTEs, it is less known as to how RTEs harness epigenetic mechanisms to benefit their own propagation, or how RTEs may exploit the vulnerability of the host genome to undermine its defense, and what the human genome uses to cope with these L1 actions.

More than 520,000 copies of L1s exist in the human genome, with about 3000–5000 being full length,^2,13^ among which ~100–140 L1s are potentially capable to transpose autonomously (i.e., retrotransposition-competent L1s or RC-L1s).^10,12,21^ About 20 RC-L1s are particularly active, and are responsible for a majority of the ongoing L1 mobilization.^2,22^ RC-L1s transcribe ~6-kb-long RNA by autonomous promoters and fulfill a life cycle of transcription, RNA processing, translation (into ORF1p and ORF2p), trafficking back to the nucleus, and finally the new genomic insertion^13^ (we sometimes refer to these as live L1s in this paper). It is important to note that while RC-L1s are the primary focus of many studies, they are actually the extreme minority as compared to the total number of annotated L1s in the reference human genome.^2,10^ Most annotated L1 sequences are truncated or mutated, incapable of coding for functional ORF1p/ORF2p, and/or have lost their 5’ends including autonomous promoters.^2,3,6^ They are retrotranspositionally incompetent, ‘dead’ L1s that represent evolutionary relics of L1 insertions in the past but were since fixed in our genome. Less is known about the potential functions and mechanisms of these dead L1s despite their > 17% occupancy of our genome.^3,6^ Importantly, the repertoire of L1 relics is continuously expanding because the great majority of RC-L1 jumping events will create new ‘dead’ L1s at the new location.^2,6^ Thus, understanding the regulation/function of both RC-L1s and the dead L1s is crucial to unravel how L1 transposition takes place in the human genome, and how L1 mobilization and deregulation may impact human development or diseases.

L1 mobilization contributes to more than 100 human genetic diseases.^23–25^ Studies reported that L1 insertion may disrupt protein sequences,^26^ can act as alternative promoters,^27,28^ or may be mis-spliced into mRNAs.^29^ However, there is an exceeding rarity of L1 insertion into coding regions,^22,30^ and most post-insertion L1s lose their 5’ end and autonomous promoters,^2,6,22^ suggesting that some other mechanisms may more widely underlie L1 impact on host gene expression. Introns harbor a large portion of the pre-existing L1s (both live and dead L1s),^1,10,12^ and are the major targets for de novo L1 insertion (~35%–50%)^22,30–34^ (although this shall be also attributed to the large portion of the genome occupied by introns^20,35^). Intronic L1s may be common functional contributors to altering the genome or changing gene expression, as have been implicated by several observations,^30,36^ but a mechanistic understanding of intronic L1s on host gene control is lacking.^6^

Human brain possesses a uniquely high somatic activity of L1s.^7,32,33,37^ Coincidentally, the nervous system also uniquely expresses a large number of long genes.^38,39^ These long genes display a particular vulnerability to deregulation in neurodevelopmental or neuropsychiatric disorders (NNDs).^39–41^ The mechanisms underlying long gene vulnerability are unclear, despite a role of DNA topological stress in their gene bodies was suggested.^39,41^ Intriguingly, increased L1 activity was often observed in the brains of NND patients, and many of L1 pathological transpositions directly landed to long gene introns.^31,34,42,43^ However, a mechanistic link between long gene control/vulnerability and the intronic L1s has not been appreciated.

L1s need to be transcribed into RNAs before they can insert back to the host genome (i.e., copy and paste).^13^ RNAs in mammals are subjected to more than 100 different types of chemical modifications, which emerge as a new paradigm in transcriptome control.^44,45^ Among these, *N*^6^-methyladenosine (i.e., m^6^A) is the most abundant, and was found important for mRNA metabolism, stability, and translation.^44,45^ The discoveries of specific enzyme complexes that methylate (“writer”) or demethylate (“eraser”) the m^6^A marks, and of proteins recognizing m^6^A marks or m^6^A-marked RNA motifs (“reader”) give birth to the concept of “epitranscriptomics” or “RNA epigenetics”.^44–47^ This was coined in analogy to the well-known concept of “epigenetics” that comprises dynamic and reversible regulation of DNA and histone chemical modifications.^48,49^ Whether RNA m^6^A modification may play roles in L1 retrotransposition or RTE–host interaction has been underexplored.

Here we report that the RTE-derived RNA transcripts, particularly evolutionarily young L1s, are heavily marked by m^6^A modification in human cells. We uncovered that m^6^A is a unique epigenetic mark that acts to promote L1 RNA expression and retrotransposition, and we further identified new m^6^A-modulated RNA binding proteins (RBPs) in human cells that act to counteract such m^6^A benefits. Unexpectedly, our results discovered thousands of sense-oriented m^6^A-marked intronic L1s (MILs) as novel regulatory elements that preferentially suppress the transcription of long human genes. This epitranscriptomic mechanism lays a new foundation to understand RTE–host interaction in gene regulation and genome maintenance, with broad implications for human development and genetic diseases, such as neurodevelopmental disorders.

## Results

### LINE-1 constitutes a major category of m^6^A-methylated RNAs in human cells

**Fig. 1:**
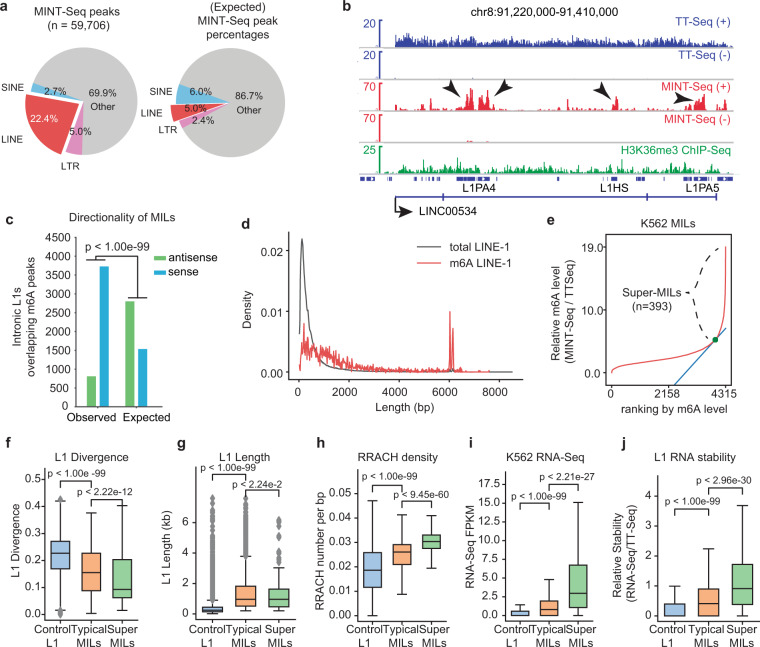
Retrotransposons and LINE-1s are highly marked by RNA m^6^A modification in the human transcriptome. **a** Pie charts showing the genomic distribution of m^6^A peaks on non-LTR (LINE, SINE) and LTR retrotransposon elements based on K562 MINT-Seq. Left, Genomic distribution of MINT-Seq m^6^A peaks. Right, Expected distribution of MINT-Seq m^6^A peaks. These expected percentages were calculated based on a null hypothesis that any transcribed regions in the genome have equal chances to contain m^6^A peaks. Thus, from the TT-Seq reads mapped to LINE, SINE, and LTR elements in the reference genome (hg19), we can deduce the peaks to be expected from these regions. **b** A snapshot of genome browser tracks of TT-Seq, MINT-Seq, H3K36me3 ChIP-Seq data in K562 cells, together with the LINE and gene annotations in genome hg19 (below the tracks). RefSeq RNA gene *LINC00534* is shown that it contains many strong intronic m^6^A peaks perfectly overlapping L1s (arrows). (+) and (−) in the data tracks indicate Watson and Crick strands. **c** A bar plot showing numbers of intronic L1s that are sense- (blue) or antisense- (green) oriented to the hosting genes. The “Expected” denote numbers calculated using all intronic L1s, while “Observed” using intronic L1s overlapping m^6^A MINT-Seq peaks. *P*-value was calculated with Fisher’s exact test. **d** A density plot showing the percentage of L1 distribution based on the length of all hg19 annotated L1s (gray) or of the m^6^A-marked L1s (red). **e** A plot showing relative m^6^A levels (MINT-Seq/TT-Seq) across all MILs (intronic L1s that overlap MINT-Seq peaks). A subset of MILs harboring exceptionally high levels of m^6^A was identified as Super-MILs (*n* = 393), achieved by using the slope of the distribution curve (blue line and green point indicate the boundary between Super-MILs and Typical MILs). **f**–**h** Boxplots showing features of Super-MILs, Typical MILs and the Control L1s (transcribed intronic L1s without m^6^A peaks), in terms of sequence divergence as compared to L1 consensus (**f**), length (**g**) and m^6^A motif (RRACH) density (**h**). *P*-values were calculated with Mann-Whitney *U* tests. **i**, **j** Boxplots of the same three groups of L1s as in the previous panels, showing their transcript levels (**i**), and relative RNA stability (calculated by taking the ratio between RNA-Seq and TT-Seq FPKM, panel **j**). *P*-values were calculated with Mann–Whitney *U* tests.

We developed a new method to examine m^6^A landscape on nascent RNAs, which we refer to as m^6^A inscribed Nascent Transcript Sequencing (MINT-Seq) (Supplementary information, Fig. S1a; Materials and Methods). This method was based on 4-thiouridine (4SU) metabolic labeling of nascent RNAs, followed by tandem purification with streptavidin beads and an m^6^A antibody, and we added dual spike-in controls to verify the purification efficiency and sensitivity. A fraction of biotin-purified 4SU-marked nascent RNA was used for Transient Transcriptome sequencing (TT-Seq),^50^ which served as the input for MINT-Seq (Supplementary information, Fig. S1a–c). Analysis of paired MINT-Seq and TT-Seq in K562 cells uncovered 59,706 m^6^A peaks on nascent RNAs transcribed in less than 5 min, as compared to 19,306 peaks found on steady-state RNAs (i.e., by conventional MeRIP-Seq with total RNA-seq as inputs) (Supplementary information, Fig. S1d and Table S1). A remarkable number of m^6^A peaks were only found in nascent RNAs (> 40k, Supplementary information, Fig. S1d, e), which are largely uncharacterized m^6^A sites that cannot be robustly detected by regular MeRIP studies (Supplementary information, Fig. S1h). A similar pattern was found in HeLa cells (Supplementary information, Fig. S1f). These nascent RNA m^6^A peaks are discovered in part due to our robust enrichment of nascent RNAs, as revealed by an extremely high intron/exon ratio in the TT-Seq (Supplementary information, Fig. S1g). Strikingly, a very high (~30%) percentage of nascent RNA m^6^A peaks overlap with annotated retrotransposons including non-LTR (e.g., LINEs) and LTR retrotransposons (e.g., ERVs), which is significantly higher than expected (Fig. 1a, left vs right). Among these, LINEs showed the highest numbers of MINT-Seq peaks (22.4% of all peaks), representing strong enrichment of m^6^A peaks (~4-fold higher than expected, Supplementary information, Fig. S2a). Consistently, L1 RNAs contain the highest levels of m^6^A among RTEs by calculating the FPKM ratios between MINT-Seq and TT-Seq (Supplementary information, Fig. S2b). LTR retrotransposons such as ERVs showed moderate levels of m^6^A; but SINEs showed no m^6^A peak enrichment and overall low methylation level (Supplementary information, Fig. S2a, b), as exemplified by Alu (a major type of primate-specific SINEs), consistent with its overall low A/T constituents.^13^ This strong enrichment of m^6^A on L1s (Fig. 1a; Supplementary information, Fig. S2a, b) suggests its yet unappreciated role in L1 expression control or mobilization.

Signals of m^6^A are particularly strong on L1s located in gene introns (Fig. 1b; Supplementary information, Fig. S2c). For example, for the LINC00534 RNA gene, while TT-Seq displays a broad and “flat” pattern across the entire transcription unit, MINT-Seq signals enrich to several “islands”, which perfectly overlap annotated L1s (Fig. 1b). Intronic L1 sequences distribute either in the same or reverse direction as the hosting genes (i.e., sense vs antisense), at a ratio of approximately 1:2 (Fig. 1c). Interestingly, m^6^A peak was strongly biased to mark intronic L1s sense-oriented to host genes, suggesting the deposition of m^6^A is likely guided by L1 RNA sequences rather than L1 DNA sequences or associated chromatin status (Fig. 1c, see below and Discussion). As m^6^A is a mark on RNAs, we used m^6^A-marked L1s to denote the RNA transcripts; whenever applicable, we used L1 regions to denote the genomic sequences.

We compared the length of m^6^A-marked L1s to all annotated L1s in the human genome and found that m^6^A-methylated L1s are generally longer and enrich full-length L1s (Fig. 1d). Based on m^6^A peaks in MINT-Seq (FDR < 0.01 by MACS2) and signals of transcription (TT-Seq, FPKM > 0.1), we identified 4315 m^6^A-methylated intronic L1s (MILs) in K562 cells (Materials and Methods; Supplementary information, Tables S2, S3). Among these, a subset of MILs harbors exceptionally high levels of m^6^A, reminiscent of the exceptionally high level of histone acetylation H3K27ac at specific enhancer regions that coined the concept of super- or stretch-enhancers^51,52^ (e.g., arrows in Fig. 1b). We therefore used an analogous computational strategy to rank MILs based on m^6^A levels, which permitted the identification of a subset of MILs with exceptionally high m^6^A levels that we referred to as Super-MILs (Fig. 1e; Materials and methods). Compared to other transcribed intronic L1s without m^6^A mark (i.e., Control L1s), Super-MILs and MILs possess lower sequence divergence as compared to L1 consensus sequence, and they also bear longer length, suggesting that they are evolutionarily younger^53^ (Fig. 1f, g). Super-MILs are the least divergent (i.e., youngest), while their length is overall similar to that of MILs. We performed de novo RNA motif analyses of m^6^A peaks on MILs, and found that the top motif was “AAAGAC”, resembling the well-known m^6^A motif “RRACH” (where R = A/G, and H = A/C/U)^54,55^ (Supplementary information, Fig. S2d). Indeed, the L1 m^6^A level was positively correlated with RRACH motif density, which was particularly high on Super-MILs, moderately high on typical MILs and low on other transcribed L1s without m^6^A (Fig. 1h). In the human cell types we studied, there are often ~200–400 Super-MILs (Fig. 1e; Supplementary information, Fig. S2f). Interestingly, the landscapes of both MILs and Super-MILs showed quite strong degrees of cell type specificity (Supplementary information, Fig. S2e, f), which is not just the consequence of cell type-specific transcription considering the fact that the majority of these MILs or Super-MILs are transcribed in other cell types (Supplementary information, Fig. S2g), suggesting that levels of m^6^A deposition are not solely dependent on L1 RNA sequences. Overall, these results suggest that a group of evolutionarily young L1s are deposited with a high level of m^6^A on their transcripts in a very early stage of nascent RNA production.

While many MILs can only be detected at the nascent RNA stage, i.e., solely by MINT-Seq (arrows in Supplementary information, Fig. S2c), Super-MILs are often readily detectable in steady-state RNA methylome by MeRIP-Seq (yellow highlights in Supplementary information, Fig. S2c). By analyzing total RNA-Seq, we found that the RNA abundance of Super-MILs was much higher than that of MILs and other intronic L1s (Fig. 1i). These results suggest that m^6^A levels positively correlate with L1 RNA stability. Indeed, by inferring RNA stability via calculating the signal ratio between steady-stage transcripts (RNA-Seq) and nascent transcripts (TT-Seq), Super-MILs were found to be more stable than typical MILs or other L1s (Fig. 1j). The high detectability of most Super-MILs and some MILs also allowed us to use published MeRIP-Seq data to analyze L1 RNA m^6^A methylome (see below).

### RNA m^6^A modification is an evolutionary feature of young L1 transcripts

**Fig. 2:**
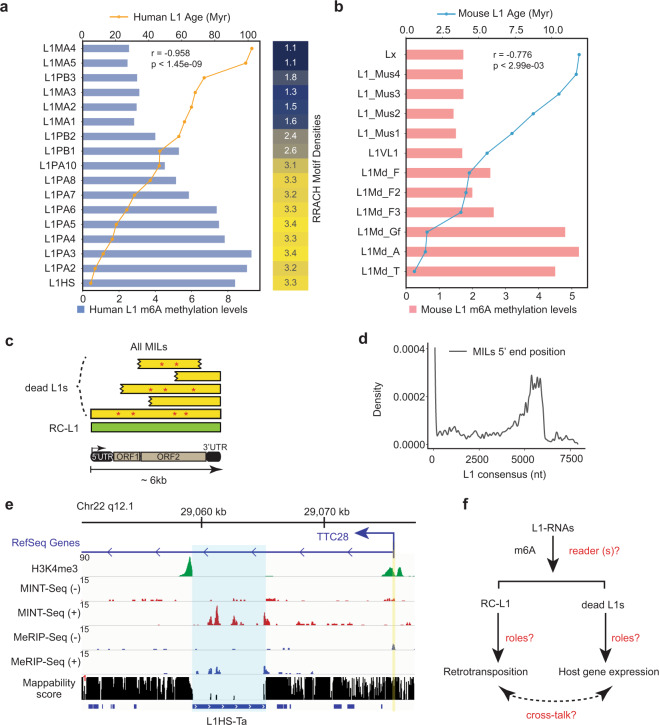
m^6^A methylation prefers evolutionarily young L1 and was deposited to both transpositionally live and dead L1s. **a** A ranked bar plot shows relative levels of m^6^A (ratios between MINT-Seq FPKM and TT-Seq FPKM) on different human L1 sub-families, and their estimated evolutionary ages (dots connected by the yellow line). The heatmap on the right shows RRACH motif densities on L1 consensus sequences (from Dfam, https://dfam.org/) of each sub-families (numbers indicate motif counts per 100 nucleotides). Myr, millions of years. The r value (correlation coefficient) and *P*-value indicate the Spearman’s rank correlation between the m^6^A methylation levels and the estimated ages of L1 sub-families. **b** A ranked bar plot generated in the same way as in panel **a** but for mouse L1s, using relative m^6^A levels calculated from published data (nuclear RNA MeRIP-Seq FPKM/RNA-Seq FPKM, Wen et al.^58^). The r value and *P*-value indicate the Spearman’s rank correlation between the m^6^A methylation levels and the estimated ages of L1 sub-families. **c** A diagram showing the features of L1s, including retrotransposition-competent L1 (RC-L1, green) and other L1s that are no longer capable to transpose (dead L1s, yellow) characterized by truncations (wedged edges) and mutations (red stars). In the lower part, a diagram of ~6 kb full-length L1 sequence with known features is shown. **d** A density plot showing the “relative” first nucleotide position of the MILs’ 5’ ends aligning them to the consensus sequences of L1s. **e** A snapshot of genome browser tracks of MCF7 H3K4me3 ChIP-Seq, MINT-Seq (±), MeRIP-Seq (±), and mappability score (from ENCODE) for the Chr22-q12.1 L1HS-Ta (in the intron of TTC28 gene in an antisense direction). Blue highlight indicates the L1HS-Ta region and yellow the TTC28 gene TSS. **f** A diagram showing the questions raised by our findings, with some of them pursued in the following part of this paper. Red text indicates some important unknowns.

We examined the evolutionary trajectory of different L1 sub-families in humans and observed a strong correlation between m^6^A levels and L1 evolutionary ages (r = −0.958, *P* < 1.45e−09, Fig. 2a), with the youngest L1 sub-families^56^ such as L1HS (a.k.a., L1PA1), L1PA2 and L1PA3 being the most methylated (Fig. 2a). We reached this conclusion by either using uni-mapped reads for analyses, or by an expectation-maximization (EM) algorithm of the *TEtranscript* pipeline to include non-uniquely mapped reads^2,57^ (Supplementary information, Fig. S3a; Materials and Methods). Interestingly, the densities of the “RRACH” motif in the consensus sequences of different L1 sub-families are also correlated with the evolutionary ages of L1s: the younger families have higher densities (Fig. 2a, right side heatmap). Looking into MILs in other species, we analyzed MeRIP-Seq of nuclear RNAs from mouse embryonic stem cells (mESCs)^58^ and identified 2033 mouse MILs (Supplementary information, Fig. S3b). Consistent with human MILs, mouse MILs are also longer than average and carry less divergent sequences from consensus (Supplementary information, Fig. S3c, d). The correlation between m^6^A levels and L1 evolutionary ages is overall conserved in mice^59^ (Fig. 2b). The youngest and retrotranspositionally active sub-families, L1Md_T, L1Md_A and L1Md_Gf, are highly methylated, and are also of higher RNA abundance (Fig. 2b). Together, these results supported that high RNA m^6^A is a conserved feature of evolutionarily younger L1s observed across species.

We queried features of the hosting genes of these evolutionarily young L1s. MILs show no obvious preference in terms of locations in the host genes (e.g., towards 5′ or 3′ ends; Supplementary information, Fig. S3e). Functional enrichment analysis of the hosting genes in K562 cells identified “regulation of double-strand break repair” (Supplementary information, Fig. S3f) as the most enriched term. Similar functional terms were also identified for MIL-hosting genes in HeLa, MCF7, and mouse ESCs (Supplementary information, Fig. S3f).

### m^6^A deposits to both autonomous RC-L1s and co-transcribed dead L1s

In most somatic tissues, live L1s are epigenetically silenced via DNA 5-cytosine methylation (5mC) and/or histone H3 methylation (H3K9me3).^14–16,60,61^ We asked what are the epigenetic features on the genomic regions of MILs. Using a published whole genome bisulfite sequencing data (WGBS) in K562,^62^ we found little enrichment of DNA 5mC on the genomic regions coding for MILs (Supplementary information, Fig. S4a). It was reported that some intronic L1s are repressed by the HUSH complex, which facilitates H3K9me3 deposition and transcriptional suppression.^63,64^ Analysis of ChIP-Seq data found mild enrichments of H3K9me3 or HUSH components (i.e., MORC2, MPP8, and TASOR) on the genomic regions of MILs (Supplementary information, Fig. S4a). Only a small fraction of MILs overlapped with H3K9me3 peaks (Supplementary information, Fig. S4b), and an even smaller number overlapped with HUSH complex binding (Supplementary information, Fig. S4c). Furthermore, H3K9me3 was deposited more often to intronic L1s that are antisense to host genes (Supplementary information, Fig. S4d), while MORC2 marked both sense and antisense L1s similarly (Supplementary information, Fig. S4e), indicating a lack of directionality preference. By contrast, m^6^A strongly prefers to be deposited to sense L1s (Fig. 1c). The deposition of m^6^A in gene regions was reported to be mediated by elongation-associated histone modification H3K36me3.^65^ By inspecting the browser tracks, we did not find strong overlaps between the m^6^A signals on L1s and the H3K36me3 peaks (Fig. 1b), which is generally applicable to all MILs (Supplementary information, Fig. S4a).

Retrotranspositionally competent L1s (RC-L1s) use their autonomous promoters near 5’end to drive transcription of a ~6kb-long intronless RNA.^66,67^ Characteristic promoter-associated histone marks H3K4me3 and H3K27ac were deposited to RC-L1 promoters.^67,68^ Analysis of published ChIP-Seq identified no enrichment of these marks at the 5’ ends of genomic regions coding for MILs, indicating that most MIL RNAs are not independently transcribed (Supplementary information, Fig. S4a). Indeed, most MILs were truncated and mutated as compared to consensus sequences (Fig. 2c, d), and have lost their promoters or 5’UTR (3390 out of 4315 have lost their 0–1 kb regions, Fig. 2d). Analyses of ATAC-Seq, GRO-CAP, other ChIP-Seq of RNA polymerase II (RNAPII) or transcription factors/coactivators reported to bind L1 promoters (e.g., YY1, MYC and EP300)^67,69,70^ showed no enrichment on the 5’ ends of genomic regions coding for MILs, which can be exemplified by two prominent Super-MILs (both are > 6 kb and are located in the introns of *PSMA1* and *ZRANB3* genes, respectively); whereas the signals are high on annotated human gene promoters/TSSs (Supplementary information, Fig. S5a, b). We experimentally used the CRISPR interference (CRISPRi) system (dCas9-KRAB together with negative control or specific gRNAs) to suppress the transcription of the *PSMA1* promoter^71^ and found concomitant reduction of both the *PSMA1* mRNA and the Super-MIL residing in its intron (Supplementary information, Fig. S5c, d). These results together indicate that the majority of MILs are not transcribed via autonomous promoters, instead they are co-transcribed with hosting genes.

Some intronic L1s were reported to be mis-spliced into hosting mRNAs.^29^ To test the commonality of this behavior for MILs, we used a de novo transcript assembly method, *Stringtie*, to identify transcripts from RNA-Seq data,^72^ and examined the frequency of MILs being spliced into mRNA transcripts (Supplementary information, Fig. S5e). As expected, most de novo transcripts overlapping annotated GENCODE genes showed multiple exons (Supplementary information, Fig. S5e, f). By contrast, when de novo called RNA transcripts overlap MILs, they are primarily single exonic, and the majority of MIL-containing de novo transcripts (346 out of 400) do not contain any GENECODE protein-coding exons, indicating that MILs are rarely spliced into host gene mRNAs (Supplementary information, Fig. S5e, g). This result can be exemplified by the raw RNA-Seq data aligned to the *ZRANB3* Super-MIL region: while exons flanking the Super-MIL are generally spliced together, Super-MIL reads are not spliced to exons (Supplementary information, Fig. S5h).

We also examined whether high m^6^A methylation applies to RC-L1s, which in humans belong to L1HS, mostly the L1HS-Ta subset (Ta: transcribed subset a).^21,73^ While the extremely repetitive nature of L1HS precludes their full alignment by short reads sequencing, there are a few that can be detected based on unique-mappable regions in the L1 body and immediate downstream sequences.^68^ Breast cancer cell line MCF7 harbors one such RC-L1 in the first intron of *TTC28* gene in the antisense direction (a.k.a., Chr22-q12.1 L1HS-Ta)^68^ (Fig. 2e). This is the most active L1 in human cancers responsible for nearly a quarter of all cancer-associated L1 retrotransposition (particularly in breast cancers it drives ~70% of retrotransposition events).^22^ MINT-Seq in MCF7 cells revealed that this L1HS-Ta RNA is highly m^6^A-methylated (Fig. 2e). In this case, contrasting most other MILs, a strong H3K4me3 peak can be seen on its promoter because RC-L1s are autonomous transcription units (Fig. 2e).

Taken together, these data demonstrated that: 1), the category of MILs is predominantly composed of retrotranspositionally dead L1s, which are not, or are weakly, associated with conventional epigenetic/chromatin states (5mC DNA methylation, histone H3K9me3, H3K36me3, or H3K4me3); 2), Super-MILs and MILs are rarely spliced to adjacent gene mRNAs, which together with the fact that their RNAs are more stable than flanking introns (Fig. 1i; Supplementary information, Fig. S5b) suggest that they are processed post-transcriptionally from introns (see discussion); 3) there is high m^6^A methylation of a single active L1HS-Ta in Chr22q12.1 (Fig. 2e), suggesting that RC-L1s share similar RNA m^6^A features as other MILs/Super-MILs. Several important questions are raised by these data (Fig. 2f): what are the potential m^6^A readers of the methylated L1 RNAs? How would m^6^A mark and its readers impact L1s, i.e., for the expression or retrotransposition of RC-L1s, or for the dead MILs to potentially impact hosting genes? What is the implication of these processes to human development or diseases?

### MILs are bound by heteromeric RBPs

**Fig. 3:**
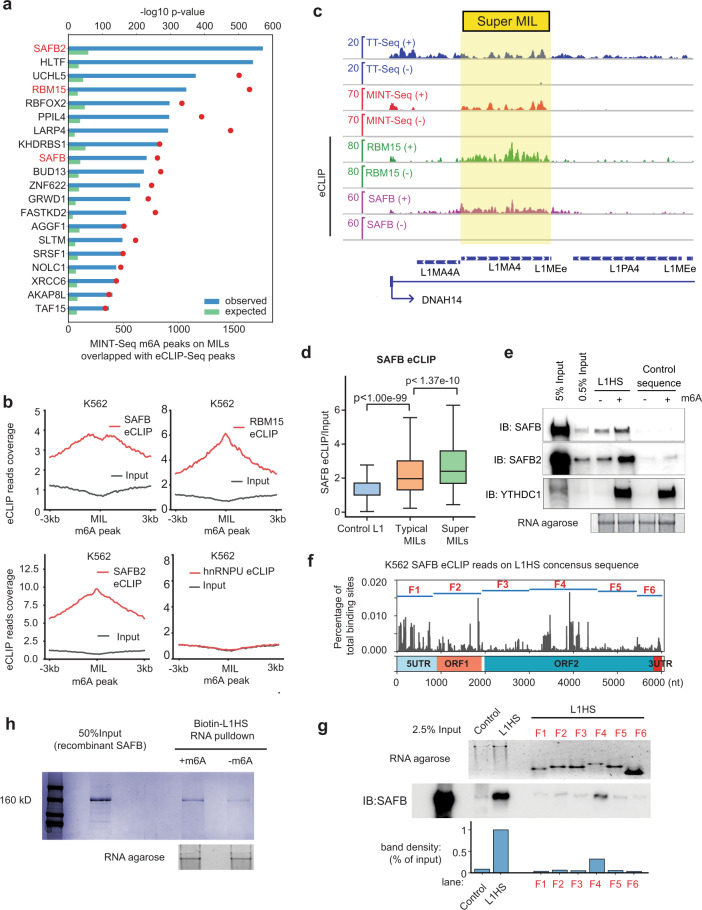
Identification of MIL-binding RBPs, with SAFB acting as a novel m^6^A-L1 reader. **a** A ranked bar plot showing the numbers of m^6^A peaks on L1s that overlap with RBP eCLIP peaks (ENCODE K562 datasets). The blue bars indicate observed numbers, and the green bars indicate expected numbers calculated using randomly shuffled regions. The statistical significance for each RBP enriching on MILs was calculated by comparing the observed to the expected numbers; the *P*-values (the red dot) are labeled based on the scale shown on top of the panel (Fisher’s exact tests). The first two RBPs (SAFB2 and HLTF) had too significant *P*-values to be included in the scale (i.e., -Log_10_ of *P*-values > 600), therefore, no red dot is shown. **b** Metagene profile plots of eCLIP-Seq signals showing the binding of SAFB, RBM15, SAFB2 and hnRNPU on MILs, with signals from the same-molecule-weight input controls (gray) plotted as background. Read densities were centered on intronic L1 m^6^A peaks (±3 kb). **c** A genome browser snapshot of TT-Seq, MINT-Seq, and multiple eCLIP-Seq data at the *DNAH14* locus. Yellow highlight indicates a Super-MIL region. **d** A box plot showing the SAFB binding intensities (eCLIP reads normalized to input) on Super-MILs, typical MILs and Control L1s (the same groups in Fig. 1). *P*-values: Mann–Whitney *U* test. **e** Western blots of SAFB, SAFB2 and YTHDC1 following biotinylated RNA pull-down using in vitro synthesized RNAs (with or without m^6^A) against K562 whole cell lysate. The RNAs were either m^6^A-marked (+) or non-methylated (−). **f** Distribution of SAFB eCLIP-Seq binding sites on the L1HS consensus sequence. Length and position of six L1HS fragments (F1 to F6) are shown and are used for RNA pull-down in panel **g**. **g** Western blots of SAFB following RNA pull-down using in vitro synthesized biotinylated L1 RNA fragments. Lower panel: quantitation of western blots showing the binding affinity between full length L1HS and its fragments with SAFB. **h** Coomassie blue staining of proteins in the biotinylated RNA pull-down using in vitro synthesized L1HS RNA (with or without m^6^A) against the recombinant full-length SAFB protein expressed in insects. The lower gel picture shows equal amount of L1HS RNAs used for pull down.

To identify potential regulatory proteins of MIL RNAs, we analyzed a large collection of enhanced Cross-Linking and Immunoprecipitation (eCLIP-Seq) data generated by the ENCODE consortium in K562 cells^74^ (Supplementary information, Table S5). By unbiasedly comparing the eCLIP binding sites of ~150 RBPs with the m^6^A MINT-Seq peaks on MILs, we identified more than a dozen RBPs that displayed particularly strong binding with MILs, including scaffold attachment factor B2 (SAFB2), its ~70% homologous paralogue SAFB, and RBM15, a nuclear adapter protein that recruits m^6^A methyltransferase to *Xist* lncRNA^75^ (Fig. 3a). Except for RBM15, none of the other MIL-RBPs has been suggested to be m^6^A regulators/readers, and their roles as RBPs are poorly characterized, particularly for SAFB2, SAFB, HLTF, UCHL5, PPIL4, LARP4, BUD13 and ZNF622 (Fig. 3a). The strong enrichment of SAFB2, RBM15 and SAFB on MILs is shown by metagene analyses of eCLIP-Seq signals (Fig. 3b), and is exemplified by the *DNAH14* locus (Fig. 3c). As a control, another abundant RBP in the nucleus, hnRNPU (a.k.a., SAF-A), displays no binding with MILs (Fig. 3b). UV cross-linking used by eCLIP-Seq predominantly reveals direct protein–RNA interactions,^74,76^ therefore, these results suggested that MILs are bound by a large collection of RBPs (which we will refer to as MIL-RBPs). The strong binding of m^6^A-MILs by these RBPs, but not by any other abundant nuclear RBPs such as hnRNPU, suggest that they may be putative novel m^6^A-RNA binding proteins (i.e., readers). One known nuclear m^6^A reader protein, YTHDC1,^44^ was not included in ENCODE eCLIP-Seq datasets. Re-analysis of published iCLIP-Seq data^75^ showed that YTHDC1 also binds MILs (Supplementary information, Fig. S6a). Our RIP-qPCR using a native antibody against YTHDC1 confirmed that it bound m^6^A-marked L1s, including L1HS and some Super-MILs (Supplementary information, Fig. S6b). We applied similar eCLIP-Seq analysis to gene 3’ UTRs, the canonical m^6^A sites on mRNAs.^55^ This analysis revealed another list of RBPs, including the recently reported m^6^A readers IGF2BP1 and IGF2BP2 (Supplementary information, Fig. S6c).^77^ We refer to this group of RBPs as 3UTR-m^6^A-RBPs, which showed a limited overlap with MIL-RBPs. RBM15 is one of the RBPs that exist in both lists (Fig. 3a; Supplementary information, Fig. S6c), suggesting that it is a shared adapter for m^6^A deposition at both locations.^75^ Some top MIL-RBPs were not found in 3UTR-m^6^A-RBPs, such as SAFB, HLTF, UCHL5, LARP4, and RBFOX2. The lack of binding of MIL-RBPs to 3’UTRs suggests that their interactions with MILs are not solely dependent on m^6^A signals.

**Fig. 4:**
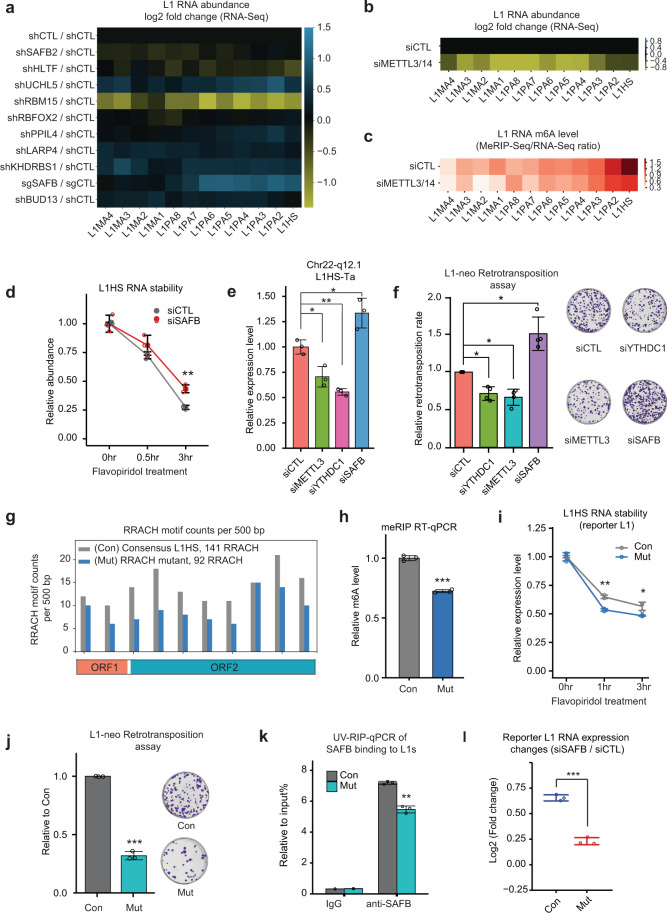
Opposite roles of m^6^A modification and SAFB on L1 expression and retrotransposition. **a** A heatmap showing the RNA expression changes of L1 sub-families after knocking down target proteins (based on re-analysis of ENCODE K562 RNA-Seq data). Fold changes were based on comparison to respective sgRNA or shRNA controls. **b**, **c** Heatmaps showing the Log_2_ fold change of L1 RNA abundances (**b**, measured by ribo-depleted total RNA-Seq) and m^6^A ratio (**c**, measured by FPKM of MeRIP-Seq / FPKM of RNA-Seq) of different L1 sub-families after co-depletion of METTL3 and METTL14 by siRNAs (siMETTL3/14). RNA-Seq after transfection by a scramble control siRNA (siCTL) was used as control. **d** A line plot showing RNA stability of L1HS after flavopiridol treatment for the indicated time. RNA abundance was calculated by RT-qPCR and normalized to 0 h time point in each group. **e** Normalized RNA expression levels of an active L1HS-Ta (Chr22-q12.1) after depletion of METTL3, YTHDC1 and SAFB by siRNA in MCF7 cells. **f** Bar plot showing the normalized L1-Neo retrotransposition activity in cells with specific target proteins depleted by siRNAs. Cell colony numbers were counted and compared to the control group. Representative pictures of cell colonies growing in a culture dish are shown on the right. **g** Sequence comparison between the L1 consensus reporter construct (Con, gray bars) and an L1 RRACH mutant construct (Mut, blue bars). The numbers of RRACH motif in each 500 bp bin throughout the L1HS are shown. **h** RT-qPCR following m^6^A RIP (meRIP) showing the relative m^6^A levels of reporter L1HS RNAs (the Con was set as 1). m^6^A levels were normalized with synthetic m^6^A-RNA spike-in. **i** Similar to panel **d**, RNA stability of Con L1HS and Mut L1HS RNAs was measured. **j** Similar to panel **f**, L1-Neo reporter assay showing the retrotransposition activity of Con L1HS and Mut L1HS. **k** UV**-**RIP assay using a native antibody against SAFB showing the normalized binding between SAFB and Con / Mut L1HS RNAs. **l** Expression changes of Con L1HS or Mut L1HS RNAs after knocking down SAFB. All qPCR data show means ± SD. *P < 0.05; **P < 0.01; ***P < 0.001, Student’s *t*-test.

Among the MIL-RBPs, we choose to focus on SAFB, which was recently reported to regulate L1 retrotransposition by a CRISPR screening.^63^ This is also due to its uniquely strong roles in affecting L1 RNA expression and retrotransposition (see below, Fig.4). SAFB is a protein associated with the nuclear matrix,^78,79^ a structure considered important for maintaining high-order chromatin architecture and gene regulation, despite controversy may exist.^80,81^ Analysis of eCLIP data showed that SAFB displayed a significantly higher affinity for Super-MILs than typical MILs or non-m^6^A marked L1s (Fig. 3d), suggesting that SAFB is potentially an m^6^A-L1-RNA reader. We used in vitro biotinylated RNA pulldown experiments to study their binding. RNAs labeled with or without m^6^A were in vitro transcribed for pull-down against K562 cell lysates. Western blots following this experiment showed that SAFB specifically binds to L1HS RNA but not to a length-matched control RNA (Fig. 3e). Importantly, the affinity of SAFB to L1HS was significantly enhanced by the presence of m^6^A, while the control RNA showed negligible SAFB binding regardless of the m^6^A status (Fig. 3e). SAFB2, similar to SAFB, exhibited stronger affinity to m^6^A marked L1HS (Fig. 3e). By contrast, a canonical m^6^A reader, YTHDC1, bound both RNAs in their m^6^A-marked forms but showed negligible affinity to non-methylated RNAs (Fig. 3e). We also tested the binding between SAFB and a Super-MIL in the PSMA1 gene, using L1HS and a non-L1 intronic region as controls (Supplementary information, Fig. S6d and Table S4). The results showed that SAFB binds the Super-MIL and L1HS with similar affinity, displaying a stronger binding to the m^6^A-labeled L1s, but it does not bind non-L1 RNA regardless of the m^6^A presence (Supplementary information, Fig. S6d).

The binding of SAFB to m^6^A-L1 transcripts may be based on specific RNA regions or motifs. To identify such regions/motifs that may explain the high affinity of SAFB binding, we re-aligned SAFB eCLIP-Seq binding sites to a pseudo genome of L1HS consensus sequence (Fig. 3f; Materials and Methods). We found a few regions of L1HS that appear to be the “high affinity” sequences (e.g., the 5’UTR, the end of ORF1 and the middle part of ORF2, Fig. 3f). Based on these, we divided L1HS into 6 fragments to perform biotinylated RNA pulldown assay (fragments 1 to 6, or F1 to F6, Fig. 3f). This experiment revealed that none of the fragments showed strong binding with SAFB (Fig. 3g), despite that the F4 (~3000–4300 nt of the L1HS consensus) possessed a detectable level of binding (Fig. 3g). We sought to identify putative SAFB binding motifs from its eCLIP binding sites on L1s, using a dedicated CLIP-Seq motif discovery tool (i.e., GraphProt).^82,83^ This identified a 5-mer putative SAFB motif consisting of A/G enriched pentamers (e.g., GAAAA, Supplementary information, Fig. S6e), consistent with a previous report by iCLIP.^84^ However, this putative motif showed a broad occurrence on the L1 sequence (Supplementary information, Fig. S6f). The density of these pentamic motifs appears similar on the full-length L1HS as compared to the L1 fragments (Supplementary information, Fig. S6f). This result suggests that the density of pentamic motifs cannot explain the selective binding of SAFB to full length L1 RNA rather than L1 fragments (Fig. 3g). In addition, we found no correlation between the density of these pentamers on each MIL and the respective SAFB eCLIP-Seq binding affinity (Supplementary information, Fig. S6g). These data indicate that the binding between L1 and SAFB is unlikely mediated by a short RNA motif, but more likely by RNA structures depending on long sequences.

To study the direct binding between SAFB/L1-RNA, we generated recombinant full-length SAFB protein and mixed it with the L1HS RNAs in vitro. This confirmed that they directly bind each other and m^6^A enhances their interaction (Fig. 3h). We further conducted a RNA competition assay to characterize the binding affinity between SAFB and L1 RNAs. In this assay, immobilized SAFB/L1–RNA complex was subjected to competition by various forms of L1HS RNA, antisense L1HS RNA or a control length-matched RNA (Supplementary information, Fig. S6h). Our results verified that SAFB binds L1 RNA in both non-m^6^A- or m^6^A-modified forms, but displaying higher affinity with its m^6^A form (Supplementary information, Fig. S6i, j). SAFB showed no detectable binding with L1 RNA in antisense direction or with a control RNA, no matter if they are m^6^A-marked or not (Supplementary information, Fig. S6i). Collectively, our results indicate that SAFB is a reader of m^6^A-L1 RNAs, but not a reader of the m^6^A mark; such “reader” behavior depends on the presence of long L1 RNA sequences and was enhanced by m^6^A. This binding is distinct from canonical m^6^A/reader binding such as that of YTHDC1, which recognizes the m^6^A mark^85^ (Fig. 3f; Supplementary information, Fig. S6d).

### m^6^A modification versus SAFB: opposite roles in controlling L1 expression and retrotransposition

We next examined the roles of m^6^A modification and MIL-RBPs. Analysis of ENCODE RNA-Seq generated in K562 cells^74,86^ showed that depletion of the top MIL-RBPs elicited variable changes of L1 RNA expression (Fig. 4a). Depletion of RBM15, a nuclear adapter that recruits m^6^A methyltransferase,^75^ markedly reduced the RNA levels of many L1 subfamilies (Fig. 4a). Knockdown of the newly identified m^6^A-L1 reader, SAFB, strongly increased L1 RNA expression (Fig. 4a), indicating that it acts as an L1 suppressor^63^; whereas knockdown of SAFB2, another top MIL-RBP and a SAFB homolog, caused negligible effects (Fig. 4a). We confirmed these changes on L1HS and two Super-MILs by RT-qPCRs (Supplementary information, Fig. S7a, b). For other newly identified MIL-RBPs, some were also found to regulate L1 RNA expression, i.e., UCHL5, KHDRBS1 and PPIL4, but the extents of L1 increases upon their knockdown were not as strong as those seen after SAFB depletion (Fig. 4a). We noticed that the impact of SAFB and RBM15 depletion on L1s was more prominent for young L1s (i.e., L1PA1-6, Fig. 4a), which correlates well with their higher m^6^A levels (Fig. 2a). Indeed, the knockdown of these two factors most prominently impacted Super-MILs as they carry highest m^6^A levels (Supplementary information, Fig. S7c). Together with the data on positive correlation between m^6^A and L1 stability (Fig. 1j), these results suggested that m^6^A deposition promotes L1 RNA expression or stability, whereas the novel L1 reader SAFB counteracts such roles.

We examined this hypothesis, first, by co-depleting the m^6^A methyltransferase (i.e., writer) complex METTL3 and METTL14 using siRNAs (Supplementary information, Fig. S7d, e). This resulted in a significant reduction of m^6^A level as well as RNA abundance of L1s (Fig. 4b, c; Supplementary information, Fig. S7d, f), indicating a positive role of m^6^A on L1 expression. The changes of L1 abundance shown by RNA-Seq were more prominent for m^6^A-marked L1 RNAs than for L1 RNAs without this mark, suggesting m^6^A dependence (Supplementary information, Fig. S7g). It is notable that the m^6^A reduction on L1s after writer knockdown was significant but incomplete (Fig. 4c; Supplementary information, Fig. S7f), consistent with a previous suggestion that even residual amounts of METTL3/14 complex may be sufficient to generate a significant level of m^6^A on many RNAs.^87^ Another RNA modification, *N*^6^, 2-O-dimethyladenosine (m^6^Am), can also be recognized by the anti-m^6^A antibody during MeRIP or MINT-Seq.^88,89^ Knockdown of PCIF1,^90,91^ the methyltransferase of m^6^Am, did not affect the expression levels of L1 RNAs (Supplementary information, Fig. S7b), suggesting this mark was not directly involved in L1 control.

To corroborate these findings, we reanalyzed a series of recently published RNA-Seq data,^58,92–97^ and found that depletion of m^6^A writers or reader (METTL3, METTL14, ZC3H13, YTHDC1) generally reduced levels of young L1 RNAs in both mouse and human cells (Supplementary information, Fig. S8a–f), indicating a positive role of m^6^A on L1 expression. In mouse, L1Md_T, L1Md_A and L1Md_Gf are the youngest sub-families and are also the main groups known to be retrotranspositionally active.^59,98^ The RNA abundances of these youngest sub-families were reduced significantly in mouse ESCs upon knockout (KO) of m^6^A writers (Supplementary information, Fig. S8a–d), correlating with their higher m^6^A levels shown above (Fig. 2b). By contrast, the abundances of some relatively older L1 RNAs, such as L1Md_F, were moderately increased or unchanged in several datasets (Supplementary information, Fig. S8a–d). Consistent with our hypothesis, L1 RNA stability was globally reduced in the absence of a m^6^A writer, as shown by re-analysis of a published time-course RNA-Seq dataset after METTL14 knockdown^65^ (Supplementary information, Fig. S8g). By contrast, L1 stability was increased by SAFB knockdown (Fig. 4d). These results indicate that m^6^A and its reader SAFB oppositely control L1 RNA expression, at least in part, via modulating RNA stability.

L1 RNA is the key intermediate for its retrotransposition. We interrogated an important question mentioned earlier (Fig. 2f): how does m^6^A mark impact L1 retrotransposition activity? By using specific RT-qPCR primers targeting the aforementioned L1HS-Ta at Chr22-q12.1 (Fig. 2e), the most active RC-L1 in human cancers,^22^ we examined its expression upon depleting SAFB, METTL3 and YTHDC1 in MCF7 cells where this L1HS-Ta is active.^68^ This experiment revealed reduction of this single live L1 (Fig. 4e), in a manner similar to pan-L1HS (the entirety of all L1HS in the genome) or other dead L1s. Importantly, by a well-established L1-neo retrotransposition reporter assay^99^ (Supplementary information, Fig. S9a), we found L1 retrotransposition activity was significantly increased after SAFB depletion, but impaired by YTHDC1 and METTL3 knockdown (Fig. 4f; Supplementary information, Fig. S9b). These changes of retrotransposition activity were not attributed to different transfection efficiency or proliferation rates because co-transfected puromycin-resistant construct led to similar cell survival rates (Supplementary information, Fig. S9c). RT-qPCR across introns or exons of the neomycin gene in the reporter showed no decrease of its splicing, ruling out that the effects were due to splicing alteration of reporter RNA (Supplementary information, Fig. S9d). Consistent with the increased L1 retrotransposition in reporter assays, after culturing SAFB-depleted cells for > 20 passages,^100^ we found a significantly higher number of L1HS in their genomic DNA (Supplementary information, Fig. S9e). There was no alteration of an inactive L1 subfamily, the L1PA2 (Supplementary information, Fig. S9e), supporting that the L1 retrotransposition changes were specific effects. The L1 copy increase was abolished in the presence of an inhibitor of reverse transcriptase, lamivudine (3TC) (Supplementary information, Fig. S9f), suggesting bona fide retrotransposition events. These data together indicated that the known m^6^A writer/reader positively promoted L1 retrotransposition, while SAFB specifically suppressed that.

### Direct and positive roles of m^6^A on L1 RNA expression and retrotransposition

Although these data are highly consistent, the knockdown of m^6^A writers can potentially affect other genes and may impact L1s indirectly. We sought to consolidate a direct role of m^6^A on L1 control. m^6^A deposits to the RRACH motif on mRNAs,^54,55,89,101^ and we found this to be consistent on L1s (Supplementary information, Fig. S2d). We therefore conducted an “m^6^A mutagenesis” experiment by generating an L1-neo reporter construct with ~35% (from 141 to 92) of its RRACH motifs mutated to lose the “RAC” (Fig. 4g; Supplementary information, Fig. S9a). We ensure this “m^6^A mutagenesis” introduced minimal L1 RNA sequence change (<1%, i.e., 55 out of ~6000 nt) and no amino acid difference. We hereafter investigate the causal function of m^6^A by comparing this m^6^A-mutant L1-neo reporter (i.e., Mut) to the original reporter with consensus L1HS sequence (i.e., Con) (Fig. 4g; Supplementary information, Table S4).

Consistent with prediction, the Mut reporter expressed an L1HS RNA of lower m^6^A level as compared to that by Con reporter (Fig. 4h). The Mut L1HS RNA was also less stable (Fig. 4i), consistent with a positive role of m^6^A on L1 RNA stability (Fig. 1i; Supplementary information, Fig. S8g). Importantly, the Mut L1HS showed a lower activity of retrotransposition as compared to Con L1HS (Fig. 4j). Northern blot showed a similar RNA profile for the two reporter L1 RNAs (Supplementary information, Fig. S9g), ruling out that RRACH mutations may cause transcriptional pre-termination or aberrant splicing of L1 RNAs. These results demonstrated a direct role of m^6^A in promoting L1 RNA stability and retrotransposition activity. We also assessed whether the binding or roles of SAFB on L1s directly depends on m^6^A. UV crosslinked RNA Immunoprecipitation (UV-RIP) assay showed that as compared to the Con L1HS, the Mut L1HS was less bound by SAFB (Fig. 4k). This is consistent with the quantitative m^6^A reduction on Mut L1HS (Fig. 4h), confirming that SAFB-L1 binding is directly mediated by m^6^A levels (also see Fig. 3). As a consequence of lower SAFB binding, the Mut L1HS RNA was less affected by SAFB depletion as compared to the Con L1HS RNA (Fig. 4l).

Taken together, these data demonstrated that m^6^A is a unique mark that benefits L1 expression (both RC-L1 and dead L1s) and retrotransposition (RC-L1s), which at least in part was mediated by RNA stability control. By contrast, SAFB is a host factor that counteracts such beneficial roles of m^6^A through directly binding m^6^A-L1s to decrease their abundance, consistent with its role as an L1 suppressor identified in a CRISPR screening.^63^

### MILs are a novel class of regulatory elements that often suppress hosting gene transcription

**Fig. 5:**
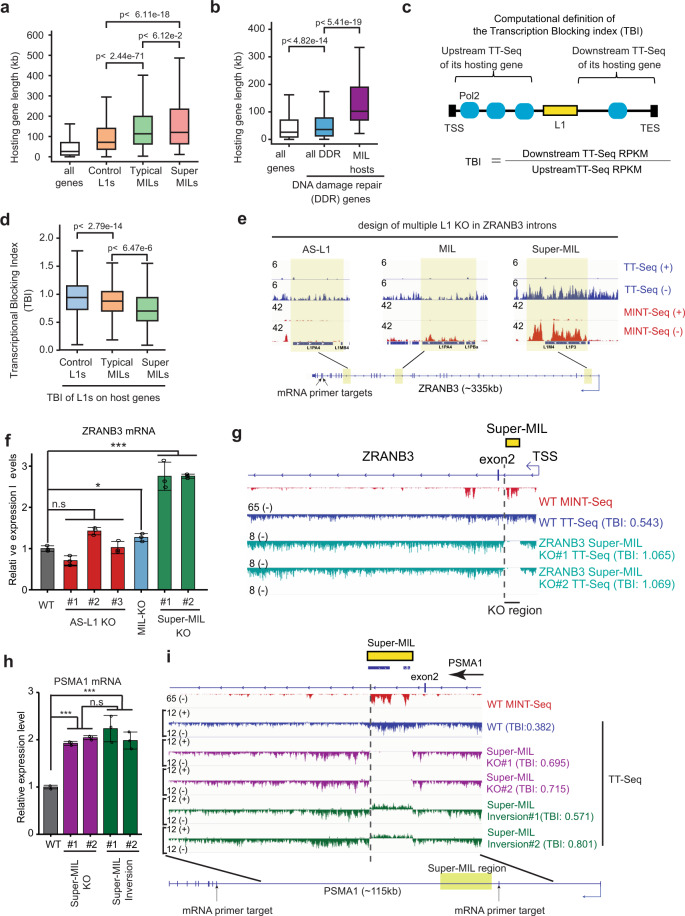
MILs enrich in long genes and represent an unappreciated category of regulatory elements that impede gene transcription. **a** A box plot showing the lengths of human genes that host Super-MILs, Typical MILs and Control L1s. The length of all hg19 RefSeq genes is also plotted as a comparison. **b** A box plot showing the lengths of human DNA damage repair genes (DDR, *n* = 448) and of DDR genes that host MILs (*n* = 37). The length of all RefSeq human genes is also shown. **c** A diagram showing the strategy to calculate the TBI for intronic L1s based on TT-Seq data. A smaller index indicates a stronger blocking role, while a number close to 1 indicates a lack of blocking. **d** A box plot showing the TBIs of Super-MILs, Typical MILs and Control L1s that indicates their impact on respective hosting genes (based on K562 TT-Seq using the equation in panel **c**). **e** Genome browser snapshots showing the KO design of three different L1s in ZRANB3 introns: an antisense L1(AS-L1, no m^6^A in MINT-Seq), a MIL (low m^6^A) and a Super-MIL (strong m^6^A). Yellow highlights show the locations of these regions in the *ZRANB3* gene (bottom of this panel). Targeted mRNA regions by qPCR primers are also shown. **f** RT-qPCR results showing the expression levels of *ZRANB3* mRNA after knocking out three different L1 regions (as in panel **g**). **g** Genome browser snapshot of TT-Seq showing the increase of transcription of *ZRANB3* gene after Super-MIL KO. MINT-Seq indicates the m^6^A levels of Super-MILs. The dash line indicates the 3’ end of the Super-MIL being deleted. WT, wildtype; KO #1/#2, two knockout cell clones. TBI of Super-MIL calculated by each TT-Seq was labeled. **h** RT-qPCR results showing the expression levels of *PSMA1* mRNA after knocking out or inversion of a Super-MIL region (see panel **i**). **i** Similar to panel **g**. Genome browser snapshot of TT-Seq showing the increase of transcription of *PSMA1* gene after Super-MIL KO and/or inversion. WT, wildtype; KO #1/#2, two knockout cell clones; Inversion #1/#2, two Super-MIL inverted cell clones. TBI of Super-MIL calculated by each TT-Seq was labeled. Primers for mRNA RT-qPCR are indicated. For all box plots, *P*-values were calculated with Mann–Whitney *U* test and are labeled in each panel. For RT-qPCR results, data show means ± SD. *P < 0.05; **P < 0.01; ***P < 0.001, Student’s *t*-test.

The large number of MILs we identified in each cell type (~2000 to > 4000 per cell type) are predominantly sense-oriented to hosting genes (Fig. 1c), are co-transcribed without autonomous promoters, and are often quite stable but are not spliced into host mRNAs (Supplementary information, Fig. S5), raising questions as to what are their impacts on host gene expression (Fig. 2f). Importantly, the intronic L1s can be either pre-existing/annotated in the human genome or be created by de novo L1 insertion.^22,30–34^ We already observed that MILs tend to exist in host genes associated with DNA damage repair and response (DDR genes, Supplementary information, Fig. S3f). Further examining MIL-hosting human genes, we found that they have a median length of >100 kb, which is significantly longer than that of all RefSeq genes, or of genes hosting non-m^6^A-L1s (Fig. 5a). Interestingly, human DDR genes (Supplementary information, Table S6) are overall longer than average, and the subset of genes that host MILs are particularly long (Fig. 5b). Disrupted transcription of long genes has been hypothesized to underlie disease etiology,^39,41,102^ but the underlying mechanisms are elusive. We are therefore curious to test a hypothesis that the MILs harbored in the long genes may regulate their expression, representing an unappreciated L1–host interaction mechanism (Fig. 2f).

Taking advantage of TT-Seq, we developed a computational strategy that we referred to as transcription blocking index (TBI) to measure the transcriptional activity of hosting genes after each intronic L1 as compared to that before it (Fig. 5c). TBI is a function for individual L1s that quantitatively reflects how strong they can impact the transcription of their hosting genes, with a number close to 1 indicating a lack of function; and the lower the TBI is, the stronger the transcription blocking effect is (Fig. 5c). Remarkably, this analysis revealed that MILs exhibited a significant transcription-blocking effect on hosting genes than that conferred by control intronic L1s without m^6^A marks (Fig. 5d). The blocking effects of MILs are correlated with their m^6^A levels, i.e., Super-MILs showed significantly lower TBIs as compared with typical MILs (Fig. 5d). This observation suggests that MILs may represent a previously unappreciated large category of transcriptional elements for human gene regulation.

To functionally validate this finding, we knocked out several genomic L1 regions coding for MIL RNAs or control L1s by CRISPR/Cas9. We selected the *ZRANB3* locus because it not only harbors a Super-MIL, but also two other L1 regions of similar length, which however differ in having lower m^6^A (a MIL) or no m^6^A (an intronic L1 anti-sense to *ZRANB3* gene, AS-L1) (Fig. 5e). KO of the Super-MIL resulted in a significantly increased expression of *ZRANB3* mRNA (~2.8-fold) in two independent cell clones (Fig. 5e, f). By contrast, the deletion of an AS-L1 region that does not generate m^6^A-RNA caused no consistent/significant change of *ZRANB3* expression in three cell clones (Fig. 5f). KO of a low-m^6^A MIL (MIL-KO, Fig. 5e) moderately increased *ZRANB3* mRNA expression as compared with WT cells (Fig. 5f). We conducted TT-Seq in the wild-type and Super-MIL KO cells to test the transcriptional basis of *ZRANB3* upregulation (Fig. 5g). In support of a role of Super-MIL as “transcriptional roadblock”, a TBI of around 0.543 in WT cells (Fig. 5g, comparing TT-Seq signals to the left vs those to the right of the dashed line) was increased to ~1.06 after the Super-MIL deletion; notably, the transcription downstream to Super-MIL was restored to a level comparable to the upstream region (Fig. 5g, comparing signals of KO and WT to the left of the dashed line). Such transcriptional “unblocking” is consistent with significant upregulation of *ZRANB3* mRNA (Fig. 5f). We observed very consistent results for another Super-MIL located in *PSMA1* intron, i.e., genetic deletion of this Super-MIL significantly increased the expression of *PSMA1* mRNA (~2-fold) in two cell clones (Fig. 5h). In this case, TT-Seq showed that the TBI was increased from ~0.38 to ~0.7 (Fig. 5i). The *PSMA1* mRNA increase was clearly due to the removal of “transcriptional roadblock” as shown by TT-Seq that the transcription increases were specific to the regions downstream of Super-MIL (left to the dashed line, Fig. 5i), whereas little difference can be found for the regions upstream of it (right side of the KO region in Fig. 5i).

Both our global analysis and locus-specific deletion of multiple L1s (Fig. 5d–i) support that only sense-direction L1s coding for Super-MIL RNAs tend to act as strong transcriptional roadblocks. To further corroborate this conclusion, and to delineate if the effects of Super-MIL deletion should be attributed to the m^6^A-L1 RNA or the DNA region, we conducted an L1 inversion experiment. We identified two cell clones in which the original Super-MIL region was inverted. This can be verified by the presence of direction-flipped TT-Seq signals from the other strand that were not seen in WT cells (Fig. 5i). In cells with L1 inversion, *PSMA1* mRNA expression was still increased by ~2-fold as compared to WT, which appeared indistinguishable from Super-MIL KO (Fig. 5h); consistently, TT-Seq showed almost identical patterns of transcription and similar TBI levels in cells with L1 KO or inversion (Fig. 5i).

Moreover, we developed an “m^6^A eraser” system by fusing catalytically-dead Cas13d^103^ (i.e., dCasRx) with either the m^6^A demethylase FTO (dCasRx-FTO) or an FTO mutant with no enzymatic activity (H231A/D233A,^104^ dCasRx-FTO-mut) (Supplementary information, Fig. S10a). Targeted m^6^A editing by wildtype FTO but not the mutant FTO on *ZRANB3* Super-MIL reduced its m^6^A level and expression, resulting in increased *ZRANB3* mRNA expression (Supplementary information, Fig. S10b–d). Together, this series of data by deleting or inverting L1s and by dCasRx-FTO editing demonstrated that Super-MILs attenuate the host gene expression by blocking its transcription, and the blocking effect is dependent on L1 RNA directionality and its m^6^A level.

### SAFB/B2 safeguard long gene transcription by antagonizing MILs as transcriptional roadblocks

**Fig. 6:**
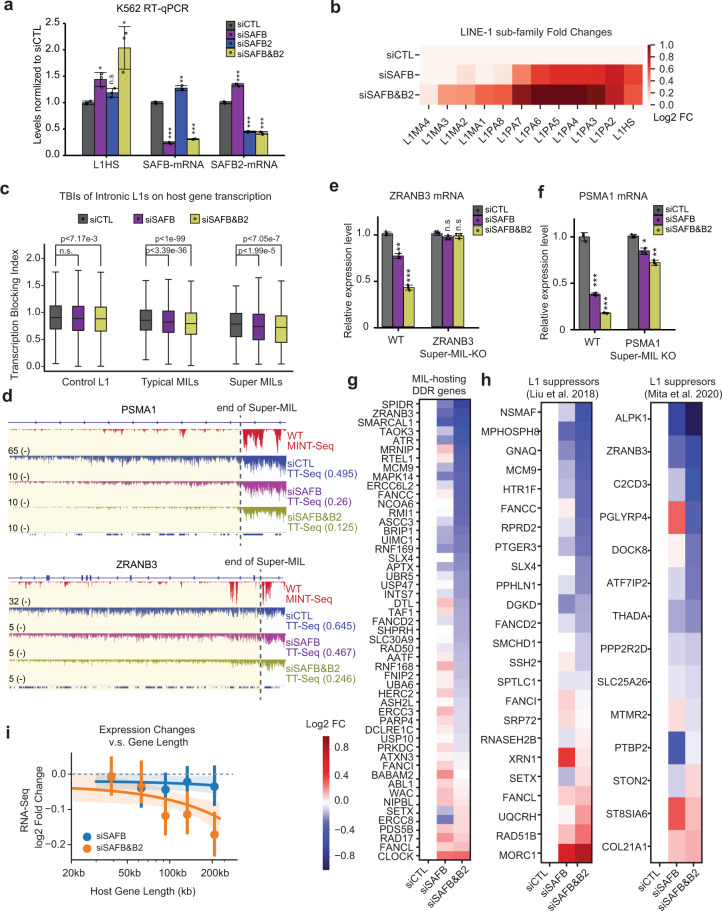
SAFB and SAFB2 safeguard host gene transcription by rectifying the transcription blocking effects of MILs. **a** RT-qPCR results showing the expression changes of L1HS after knocking down SAFB or SAFB2 in K562 cells by siRNA. The mRNA levels of SAFB and SAFB2 are also shown. siSAFB or siSAFB2 indicates single knockdown; siSAFB&B2 indicates dual depletion. **b** A heatmap showing the RNA abundances of different L1 sub-families after depleting SAFB (siSAFB) or co-depleting SAFB and SAFB2 (siSAFB&B2) by siRNAs. Data are from RNA-Seq and indicate Log_2_ fold changes of expression in the knockdown group as compared to siCTL group. **c** Box plot showing the TBIs (see Fig. 5c) of Control L1, Super-MILs or typical MILs on their hosting genes. Data are based on TT-Seq in K562 cells with the same groups of knockdown as in panel **b**. *P*-values were calculated with a paired Student’s *t*-test. **d** Genome browser snapshot showing TT-Seq signals over the *PSMA1* (upper) or *ZRANB3* gene loci (lower) in control or specific knockdown cells as indicated. The MINT-Seq track on top of each plot indicates m^6^A signals for strong Super-MILs. The dash lines denote the 3’ end of the Super-MIL regions. Yellow highlights point to regions with strong transcriptional change. TBI of Super-MIL in each TT-Seq is labeled. RT-qPCR result showing mRNA reduction of *ZRANB3* (**e**) and *PSMA1*(**f**) after depletion of SAFB (siSAFB) or co-depletion of SAFB and SAFB2 (siSAFB&B2) in wild type (WT) K562 cells or corresponding Super-MIL knockout cells. **g** Heatmaps generated by analyzing RNA-seq of siCTL, siSAFB and siSAFB&B2 K562 cells, showing the fold changes of MIL-hosting genes that are DDR genes. Scale bars are shown on the right (i.e., downregulated genes are in blue colors). **h** Similar to panel **g**, but these plots show fold changes of MIL-hosting genes that are putative L1 suppressors (by Liu et al.,^63^ left panel; and by Mita et al.,^11^ right panel). **i** A regression plot showing the relationship between gene length and their expression changes after SAFB or SAFB&B2 depletion in K562 cells. MIL-hosting genes were divided into five equal-numbered groups by length (*x*-axis), and the fold changes of each group are shown (the dots indicate mean fold changes for genes of each group, and vertical lines denote 95% confidence interval of mean fold changes). The lines show linear regression and the shaded areas of matched colors denote a 95% confidence interval for that regression. There are 422 long genes (> 200 kb) harboring MILs. Data of siSAFB or siSAFB&B2 in this figure were based on polyA selected RNA-Seq. For all qPCRs, data show means ± SD. *P < 0.05; **P < 0.01; ***P < 0.001, Student’s *t*-test.

Although SAFB2 knockdown alone caused negligible effects on L1 expression (Fig. 4a; Supplementary information, Fig. S7b), it interestingly exacerbated L1HS increase in cells depleted of SAFB (Fig. 6a). This finding suggests a partial functional compensation between these two homologous proteins on MIL RNA control, reminiscent of a similar phenomenon of YTHDF readers on mRNA control.^105^ In accord, RNA-Seq validated this collaborative suppression of L1 RNAs by SAFB and SAFB2 that siSAFB&B2 caused stronger increase of L1 abundance than siSAFB alone (Fig. 6b). The double knockdown specifically affected the m^6^A-marked L1s (Supplementary information, Fig. S10e), consistent with their preferential binding on m^6^A-L1 RNAs.

The broad “over-activation” of MILs after SAFB&B2 depletion has provided an opportunity to examine their regulatory roles on host gene transcription in a global manner. We conducted TT-Seq in cells depleted of SAFB alone or of both SAFB&B2, and calculated the TBI for intronic L1s on hosting genes (Fig. 5c). This analysis revealed that SAFB knockdown reduced the overall TBIs of Super-MILs and MILs, indicating “transcriptional blocking” of hosting genes by Super-MILs (Fig. 6c). In accord, siSAFB&B2 double knockdown exacerbated this effect even more (Fig. 6c). We ranked all MILs based on their delta TBI (TBI in siSAFB&B2 – TBI in siCTL), finding a general reduction by siSAFB&B2, with ~37% of MILs showing a TBI decrease greater than 0.1 (Supplementary information, Fig. S10f, g). The MILs with decreased TBIs are of higher m^6^A levels than those not showing obvious changes (Supplementary information, Fig. S10h), in support of m^6^A-dependent regulation of TBIs by SAFB&B2. As examples, TT-Seq tracks are shown for two Super-MILs in *PSMA1* and *ZRANB3* introns, which illustrated that they became strong “transcriptional blockade” (Fig. 6d). The TBIs were accordingly reduced (labeled on the plots) after depletion of SAFB/SAFB2. The enhanced blocking by over-active MILs is highly consistent with a loss of blocking upon MILs KO in Fig. 5g, i. These results together demonstrated that a large group of MILs are intronic transcriptional roadblocks, acting in a manner dependent on their m^6^A levels; SAFB and SAFB2 function in a collaborative manner to rectify the transcriptional defects of host genes caused by such roadblocks.

### A novel L1–host interaction between MILs, SAFB/B2 and host genes

As a result of “MIL transcriptional blockade”, mRNA levels of hosting genes such as *PSMA1* and *ZRANB3* were significantly decreased by either SAFB or SAFB&B2 knockdown (left halves of Fig. 6e, f), which is consistent with their increases elicited by MIL KO (Fig. 5f, h). Importantly, these two host genes were not or less affected by siSAFB or siSAFB&B2 when their Super-MILs were deleted (right halves of Fig. 6e, f), indicating that SAFB and SAFB/B2 act on these genes directly via the Super-MILs.

The apparent suppressive roles of MILs reminded us of the interesting features of MIL hosting genes, DNA damage repair (DDR) genes and long genes (Fig. 5a, b; Supplementary information, Fig. S3f). Inspection of MIL-hosting DDR genes identified *ZRANB3*, *SMARCAL1*,* ATR*, *ATRX*, *RB1*, *FANCC*, *FANCD2*, *FANCI* (Supplementary information, Table S6), many of which are important genome guardians that may prevent L1 mobilization.^11,19,20,63^ As an example, ZRANB3 is a DNA translocase crucial for replication fork maintenance,^106^ and was recently revealed as a suppressor of L1 retrotransposition.^11^ This gene was extensively shown to harbor strong MILs and was suppressed by them (Figs. 5e–g, 6d, e). Globally, DDR genes that host MILs commonly displayed a reduced expression upon SAFB or SAFB&B2 knockdown in RNA-Seq (Fig. 6g). The effect was stronger after SAFB&B2 co-depletion (Fig. 6g), consistent with a more dramatic increase of MIL expression (Fig. 6a–c**)**. This unbiased analysis not only revealed reduction of the few genes mentioned earlier (*ZRANB3*, *SMARCAL1*, *ATR*, *FANCD2*, and *FANCC*),^106^ but also identified other DDR genes as hosts for, and were suppressed by, MILs, such as *SPIDR*,^107^ *ERCC6L2*^108^ and *BRIP1* (BRCA1 interacting protein, a.k.a., *FANCJ*^109^) (Fig. 6g, left panel). We directly compiled two separate lists of MIL-hosting genes that have been identified as L1 suppressors.^11,63^ A number of them showed significantly inhibited expression upon MIL over-activation (i.e., by SAFB or SAFB&B2 depletion) (Fig. 6h). The reduced expression of MIL-hosting DDR genes by SAFB&B2 knockdown was consistently found in other cell types we examined (Supplementary information, Fig. S10i).

Another major feature of MIL-hosting genes is that they are very long (Fig. 5a, b). To test whether long genes are more vulnerable to MIL blocking, we plotted hosting gene length against their expression changes after SAFB or SAFB&B2 knockdown. This analysis showed an interesting trend that long genes were preferentially, and more significantly suppressed (Fig. 6i), in support of the notion that MILs are important regulators of long human genes. In addition, longer genes bear on average more MILs (Supplementary information, Fig. S10j), which may contribute to the stronger changes of long genes by siSAFB&B2. For example, human *ZRANB3* has a length of 336 kb, and its expression was reduced by ~60% after depletion of SAFB&B2 (Fig. 6e), whereas Super-MIL KO increased the expression by ~3-fold (Fig. 5h). As controls, we ruled out that transient knockdown of SAFB&B2 may impact overall chromatin state that indirectly caused the expression changes of L1 RNAs or host genes, for example, H3K9me3, the histone modification often associated with heterochromatin and RTE suppression, was not affected (Supplementary information, Fig. S10k).

### SAFB/B2 and MILs impact long genes involved in crucial neuronal/synaptic functions

**Fig. 7:**
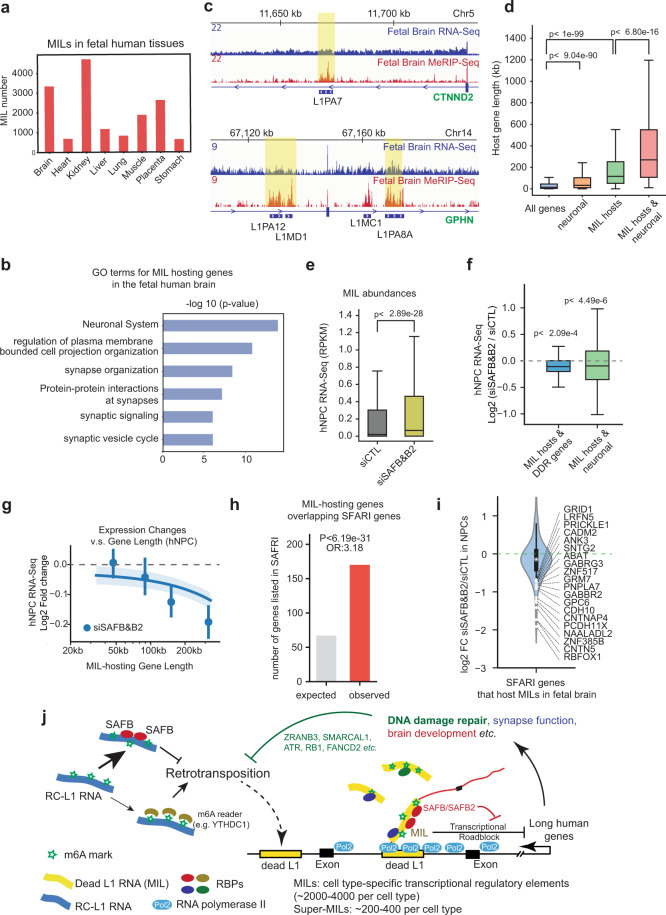
MIL landscapes in human fetal tissues, and their impediment of long neuronal/synaptic genes vulnerable to deregulation in neurodevelopmental diseases. **a** A barplot showing the MIL numbers identified by MeRIP-Seq in 8 fetal human tissues (Xiao et al.^112^). **b** The top functional gene categories that MIL-hosting genes in the fetal human brain are enriched against fetal brain expressed genes (RNA-Seq FPKM > 0.1) (by Metascape; see Materials and methods). **c** Genome browser snapshots showing tracks of fetal brain RNA-Seq and MeRIP-Seq at the CTNND2 and GPHN loci. MILs are highlighted. **d** A boxplot showing the length of four groups of genes. All genes, all RefSeq genes; neuronal, genes associated with neuronal or synaptic functions; MIL hosts, fetal brain genes that host MILs; MIL hosts & neuronal, the shared group between these two (gene lists in Supplementary information, Table S7). **e** A boxplot showing the expression levels of MILs after co-depletion of SAFB and SAFB2 as compared to control (siCTL). Data was generated from polyA RNA-Seq in human NPC cells. **f** A boxplot showing the expression changes of MIL-hosting DDR or neuronal/synaptic genes after siSAFB&B2 knockdown. The *y*-axis indicates Log_2_ fold changes based on hNPC RNA-Seq. **g** A regression plot showing the relationship between gene length and their expression changes after SAFB&B2 depletion in human NPCs, similar to Fig. 6i. **h** A barplot showing the observed or expected numbers of brain MIL-hosting genes that overlap with SFARI autism-associated genes. The expected number was calculated based on that all genes expressed in hNPCs (*n* = 19,286) have a chance to be SFARI genes; *P*-value and odds ratio: Fisher’s exact test. **i** A violin plot showing the Log_2_ fold changes of MIL-hosting genes listed in the SFARI database (the red group in Panel **h**) after SAFB&B2 co-depletion. The 20 most down-regulated genes are labeled. **j** A model figure showing the major findings of this work: 1), m^6^A on RC-L1s promotes the retrotranscription activity of these live L1s; 2), m^6^A on retrotranspositionally dead MILs mediates their roles in acting as transcriptional roadblocks that preferentially impede long human genes, which include DDR genes and neuronal/synaptic genes; 3), the m^6^A-L1 readers SAFB and SAFB2 represent a host defense system that on one hand binds RC-L1s to inhibit their expression and retrotransposition, and on the other hand reduces MIL expression to safeguard the transcription of long human genes; 4), MILs represent an unappreciated large category of cell-type-specific transcriptional elements for gene regulation in health or diseases. The colored round objects denote m^6^A-L1-binding RBPs that often associate with the nuclear matrix. Arrows indicate positive regulation or Pol2 direction, while “----|” indicates negative regulation or suppression. The *P*-value for panel **d** was calculated by Mann-Whitney *U* test; *P*-values in panels **e** and **f** were calculated with paired Student’s *t*-tests (siSAFB&B2 vs siCTL). Data in this figure of hNPC after siSAFB&B2 was based on polyA selected RNA-Seq.

This intriguing cross-talk between MILs, DDR factors and long genes is reminiscent of a noted long gene enrichment/regulation in the brain,^38,39^ particularly because both DDR^110,111^ and L1 activity^7,32,33,37^ were uniquely crucial in this tissue. Long genes are associated with vital neuronal functions and are involved in NNDs.^38–40^ We hence interrogated potential roles of MILs as unappreciated regulatory elements in the human brain. By analyzing published MeRIP-Seq datasets in fetal human tissues,^112^ we identified a large number of MILs in each tissue, among which the fetal brain contains one of the largest (*n* = 3339) (Fig. 7a). MIL-hosting genes in the fetal brain are enriched for functional terms “neuronal system” “cell projection”, “synapsis organization” and “synaptic genes” (Fig. 7b), a large number of which are crucial for neuronal and synaptic functions. As examples, strong MILs were harbored in key neuronal/neurodevelopmental genes *GPHN* (Gephyrin),^113^ *UBE3A*,^114^ and *CTNND2* (delta2-catenin)^115^ (two examples shown in Fig. 7c), in synaptic genes such as *DLG2* (coding for the postsynaptic density protein-93),^33^ in genes coding for crucial transmembrane molecules (*CNTNAP4* and *CTNNA2*),^116,117^ and in major neurotransmitter receptor genes such as GABA receptor type-A γ3 (*GABRG3*) and Glutamate Receptor AMPA Type-4 (*GRIA4*) (Supplementary information, Table S7). DDR genes were also enriched as MIL hosts in the fetal brain, but were not as highly ranked as neuronal or synaptic genes (not shown). In terms of gene length, neuronal/synaptic genes are longer than average (Fig. 7d), which is a known feature,^38,39^ but neuronal/synaptic genes that harbor MILs are exceptionally long (Fig. 7d; Supplementary information, Table S7).

Human neural progenitor cells (hNPCs) were known to harbor high L1 activity.^118^ To confirm the roles of MILs/SAFB&B2 in brain cells, we generated hNPCs from induced human pluripotent stem cells (iPSCs) with a high purity, as shown by expression of marker genes *NESTIN*, *SOX1* and *SOX2* in immunofluorescence (Supplementary information, Fig. S11a). Co-depletion of SAFB&B2 in hNPCs demonstrated their highly conserved function as we found in other transformed cells (Fig. 6). First, their co-depletion strongly induced L1 RNAs, to a level higher than that by SAFB knockdown alone (Supplementary information, Fig. S11b); and RNA-Seq analyses confirmed a global increase of MILs (Fig. 7e). Second, the upregulation of MILs was accompanied by a significant decrease of their hosting genes, including DDR genes and a large category of neuronal/synaptic genes (Fig. 7f). The genes down-regulated by SAFB&B2 knockdown (FDR < 0.05 by EdgeR) were highly enriched for functions related to nervous system development (Supplementary information, Fig. S11c). Third, when we ranked the MIL-hosting genes in NPCs by their length, it was obvious that long genes, especially those >100 kb, were particularly inclined to be inhibited (Fig. 7g).

Human brain also contains a myriad of non-neuronal cells that are not differentiated from NPCs. Microglia is the resident macrophage in the brain crucial in development and diseases,^119,120^ but L1 expression and function in this cell type are less explored. We conducted TT-Seq/MINT-Seq in HMC3 cells, a transformed primary cell type of embryonic human microglia,^121,122^ which identified 1607 MILs (Supplementary information, Fig. S11d). In contrast to the fetal human brain, the GO terms for MIL-hosting genes in HMC3 are similar to those in other somatic cell lines, such as DDR genes (Supplementary information, Fig. S11e), suggesting a unique program of MILs in the cells of neuronal lineage. Consistently, RT-qPCR showed that SAFB also suppressed L1 RNA expression in human microglia (Supplementary information, Fig. S11f). These results together established a general mechanism that SAFB&B2 suppressed MILs in long genes to safeguard their transcriptional output and critical functions.

### Implication of MILs in neurodevelopmental diseases

Aberrant L1 activity has been widely observed in NNDs.^31,34,43^ Of the genes affected by MILs/SAFB&B2, a large number are associated with NNDs. We examined the list of genes compiled by the SFARI database that are implicated in autism spectrum disorder (ASD)^123^ (https://www.sfari.org/resource/sfari-gene/). There is a strong association between autism-associated genes and MIL-hosting genes in the fetal brain (170 out of 861 SFARI genes are MIL hosts, Fisher’s exact test, *P*-value < 6.19e−31, Fig. 7h). Notably, the SFARI genes that host MILs are largely down-regulated in hNPCs after SAFB&B2 depletion (Fig. 7i; Supplementary information, Fig. S12a), including many aforementioned genes crucial in synaptic and neuronal functions (top 20 down-regulated genes shown in Fig. 7i). SAFRI genes without MILs were largely unaffected (Supplementary information, Fig. S12b), supporting that the effects of SAFB&B2 on neuronal/synaptic genes are specifically mediated by MILs.

## Discussion

Here, we report a remarkable enrichment of m^6^A modification on RTEs, making them major categories of m^6^A-marked RNAs in the human transcriptome. These findings revealed interesting convergence and distinction on retrotransposon control dictated by the methylation of DNAs, histones or RNAs. As parasitic genetic materials that threaten host genome stability, RTEs such as L1s are suppressed by sophisticated strategies of the hosts, notably, by methylation of DNA (i.e., 5mC) and of histone tails (e.g., H3K9me3).^14,61^ Our current findings demonstrated that methylation of RNAs also controls RTEs, but acts as a unique epigenetic mark to benefit L1 propagation, opposite to the suppressive roles of DNA and histone H3K9 methylation. We found that RNA m^6^A predominantly enriches on transcribed intronic L1s. In addition, RNA m^6^A prefers young L1s that are overall longer than average, and are in the same direction as the host genes, largely due to the enrichment of RRACH motifs to permit m^6^A deposition. Remarkably, the genes hosting intronic m^6^A-L1s are often associated with essential functions for cell survival (e.g., DDR). We are tempted to speculate that these genes are largely “un-silenceable” by DNA or histone methylation. As a result, many young L1s that “hide” in their introns can be co-transcribed together with the hosting genes. Interestingly, the *N*^6^-methyladenosine on DNA was also observed to exist on L1s in mouse ESCs, although its abundance is extremely low.^124^ Whether RNA and DNA m^6^A methylation on L1s crosstalk to each other in development or disease and how that mediates L1 activity or host gene expression are interesting questions for future investigation.

From the viewpoints of the L1s, it is interesting that m^6^A orchestrates a beneficial crosstalk between the living and the dead. Only ~100 RC-L1s in human genomes possess autonomous mobility, and the large majority of annotated L1s (> 520,000 copies) are transpositionally ‘dead’.^2,6,12^ Our current study provided two important insights: 1) RNA m^6^A modification represents a unique epigenetic mark harnessed by RC-L1s to self-benefit, at least in part through increasing RNA stability; 2) m^6^A-marked dead MILs act as transcriptional roadblocks to impede key host genes with roles in L1 suppression to indirectly support L1 transposition (Figs. 5, 6, 7j). ZRANB3 is a great example to illustrate this living/dead crosstalk. As a DNA translocase crucial for replication fork stability,^106^ ZRANB3 was recently found to be a strong suppressor of L1 retrotransposition.^11^ We showed that *ZRANB3* hosts several Super-MILs that can compromise its gene transcription, as revealed by both genetic MIL knockout and by siSAFB/B2 knockdown (Figs. 5, 6). These data elucidated an important, previously unappreciated interaction between the living and dead L1s to benefit the propagation of their species (Fig. 7j).

Our conclusion that m^6^A positively controls L1 expression and retrotransposition is based on a series of knockdown of m^6^A writers/reader, on re-analyzing ENCODE and other datasets, and by employing L1 retrotransposition reporter assays (Fig. 4; Supplementary information, Figs. S7, S8). Going beyond commonly used L1HS qPCR that detects a mixture of L1HS without clear identity/location information, we extended our experiments to a single hot L1HS-Ta that locates in Chr22-12.1, which is the most active L1 in human cancers.^22^ We found its high m^6^A modification as well as regulation by m^6^A regulators, analogous to pan-L1HS or dead MILs (Figs. 2e, 4e). These data documented a positive role of m^6^A in L1 RNA expression and mobilization. A recent report used permanent depletion of m^6^A enzyme METTL3 or reader YTHDC1 in mouse ESCs, and found an increased stability of “chromosome-associated RNAs” including mouse L1 RNAs.^93^ But this work focused on L1Md_F,^93^ a relatively old mouse L1 that was not known to bear retrotransposition activity. Our reanalysis of published data^58,92–97^ showed that depletion of m^6^A writers or reader (METTL3, METTL14, ZC3H13, YTHDC1) generally reduced levels of the youngest/live L1 RNAs in both mouse and human cells (Supplementary information, Fig. S8a–f), supporting a positive role of m^6^A for young L1 expression and retrotransposition. But some older and lower methylated L1s such as L1Md_F can be sometimes increased after m^6^A writer depletion (Fig. 2b; Supplementary information, Fig. S8a, b).

Importantly, our results went beyond knockdown of m^6^A regulators. We mutated more than one-third of RRACH motifs on the L1HS RNA sequence and proved a direct role of m^6^A, i.e., this consistently reduced m^6^A levels and RNA stability, and dampened L1 retrotransposition (Fig. 4). These results, to our knowledge, represent one of the first experiments that conducted m^6^A site mutations on a long RNA, having clearly demonstrated a direct and positive role of m^6^A on L1 expression and mobilization in human cells. Our data also raised important questions for future pursuit, for example, how exactly does m^6^A promote L1 RNA stability, or are there additional mechanisms that underlie its positive roles on RC-L1 activity, such as nucleus-cytosol trafficking or RNA translation efficiency. Both of these functions are important for RC-L1 to fulfill a complete life cycle, and both have been functionally connected to m^6^A on mRNAs.^96,105,125–128^

From the viewpoints of the host genome, our results revealed several RNA binding proteins as previously unappreciated defense factors. L1s are parasitic genetic elements in the human genome.^3–9^ In response to the m^6^A benefits to L1s, the host cells utilized m^6^A-L1 reader proteins particularly the SAFB&SAFB2 to counteract: 1), for both the living RC-L1 and dead MILs, SAFB/SAFB2 bind their RNAs in an m^6^A-enhanced manner to suppress their expression, at least in part by decreasing RNA stability; 2) for the transpositionally-dead intronic MILs, SAFB inhibits their expression and rectifies their transcription blocking effects on important host genes (Figs. 5,6,7j). Notably, our data suggest that SAFB binds m^6^A-marked L1 RNAs via a process distinct from canonical m^6^A readers such as the YTH proteins, i.e., SAFB does not specifically recognize the m^6^A mark.^44,85^ Indeed, short motifs identified from SAFB eCLIP-Seq cannot explain the binding between SAFB and L1 RNAs. L1–SAFB binding assays demonstrated that even long fragments of L1s (e.g. ~1 kb) that carried a comparable density of predicted motifs cannot bind SAFB well (Fig. 3f, g). These results are in favor of a mechanism that L1 RNAs bind SAFB via specific high-order structures, with the presence of m^6^A quantitatively enhances, rather than indispensably licensing, their interaction. As m^6^A has been shown to alter local RNA structure to confer RNA–RBP interaction (i.e., “m^6^A switch”),^129^ we propose that m^6^A may regulate higher-order L1 RNA structures to permit stronger L1–SAFB binding.

An unexpected advance from our work is that we established MILs as a large category of overlooked regulatory elements for gene transcriptional control. Indeed, as compared to extensive studies of RNA polymerase II (RNAPII) action at the stages of transcription initiation, pause release and elongation,^130^ research on its processivity control in gene bodies, particularly in long genes, is a minimally explored area. The number of MILs (~2,000–4,000 per cell type) enlisted them as a large class of elements with comparable numbers in each cell type as other well-known elements such as enhancers.^51,131,132^ Analogous to enhancers, MILs exhibited cell type specificity, as well as computational features of being ‘typical’ or ‘Super’. Our data indicated that MILs function in suppressing DDR genes in a tissue-invariant manner but regulate neuronal/synaptic genes in a tissue-specific fashion. These findings thus add novel insights to the regulatory roles played by RTEs in host gene regulation or genome evolution.^3,4,9^ Moreover, we provided mechanistic evidence by TT-Seq that the roles of MILs lie in their ability to impede RNAPII transcription. Our computational attempts, i.e., by defining TBIs for intronic elements, pave way for systematically examining the intronic control of RNAPII processivity in health and disease. The exact mechanisms of MILs on blocking host gene transcription remain unclear at this stage. We observed some overlap between Super-MILs and R-loops by re-analyzing a published dataset^94^ (data not shown). However, future work is warranted to elucidate the complete mechanisms by which the m^6^A-marked intronic L1 RNAs impede host genes, which may involve R-loop formation and/or dissolving.

It is generally thought that introns are quickly degraded after splicing, except in rare stress conditions.^133,134^ Here, our data revealed that a portion of introns, i.e., the L1s embedded therein, can be made into stable RNAs (Supplementary information, Fig. S5). We further found that MILs are bound by a dozen of RBPs that seem to often associate with the nuclear matrix (Fig. 3). Intriguingly, Hall et al. has found that a large quantity of chromatin-associated RNAs can be detected by CoT1 FISH probes and they stably stay on chromatin over time, of which a large portion was considered to be L1 RNAs.^80,135^ In this light, our results support that m^6^A-L1 RNAs from introns constitute an important portion of L1 transcripts on chromatin, and may underlie the observed importance of L1 RNAs in regulating development and gene expression.^80,135^

Several unusual features of MILs suggest yet unknown post-transcriptional RNA processing and m^6^A deposition mechanisms. First, MILs are predominantly sense-oriented and largely co-transcribed with host genes without separate promoters, yet they mostly do not splice into neighboring mRNA exons (Supplementary information, Fig. S5). Second, MILs are highly-m^6^A marked but little m^6^A signals can be seen on the flanking intron sequences; this observation supports a notion that MILs are processed from introns into separate transcripts, but the flanking intron regions are removed (Fig. 1b; Supplementary information, Fig. S5). Third, the presence of m^6^A on MILs cannot be well explained by current models of m^6^A deposition via a co-transcriptional process facilitated by the H3K36me3 mark (Fig. 1b; Supplementary information, Fig. S4a). In-depth understanding of these observations demands new knowledge. Their further characterization will shed new light on L1–host interaction, intronic RNA processing and the specificity/selectivity of RNA m^6^A deposition.

The occurrence of MILs is not random and shows an intriguing preference for long genes. Unexpectedly, this can be functionally connected to an important but mechanistically elusive observation of long gene vulnerability in human NNDs.^39^ Long genes harbor long introns and may therefore unsurprisingly have higher chances to harbor intronic L1s.^38^ But MIL-hosting brain genes are particularly long, much larger than average neuronal/synaptic genes, or than those genes that harbor non-m^6^A-marked control L1s. Importantly, MIL-hosting brain genes showed a significant overlap with autism-associated genes (e.g., SFARI). These genes displayed length-aggravated defects in human NPCs in the absence of SAFB&B2, indicating that they are more prone to impediment by MILs (Fig. 7g). Interestingly, SAFB and SAFB2 showed high expression in the brain, most prominently in the hippocampus and cerebellum,^79^ coinciding with the high activity of L1 in the hippocampus.^32,33,136^ Our data suggest a possibility that SAFB&B2 may play important roles in specific brain regions to properly control L1s and to safeguard long gene transcription.

Collectively, the comprehensive landscape of m^6^A RNA methylome on RTEs we revealed, together with the conceptual frameworks we build in this study, lay the foundation for a largely unexplored area of epitranscriptomic regulation of RTE–host interaction in development and disease.

## Materials and methods

### Cell culture

K562 cells were a gift from Dr. Yun (Nancy) Huang lab from Texas A&M University Health Science Center Houston, and were originally purchased from ATCC. They were cultured in RPMI 1640 medium (CORNING) supplemented with 10% fetal bovine serum. HeLa, MCF7 and 293T cells were purchased from ATCC, and cultured in DMEM medium (CORNING) supplemented with 10% fetal bovine serum. HMC3 cells are immortalized primary human embryonic microglia cells, and were purchased from ATCC. They were cultured in EMEM medium supplemented with 10% FBS. hNPCs were generated by an in-house protocol (see below). Mycoplasma was examined every 6 months to a year.

### Antibodies

Antibodies were used at the indicated dilutions as follows: anti-SAFB (Bethyl, A300-812A, 1:1000 for Western blots and RIP-qPCR), anti-YTHDC1 (Bethyl, Cat# 305-096A, 1:1000 for Western blots and RIP-qPCR), anti-METTL3 (Synaptic Systems, Cat# 417-003, 1:1000 for Western blots), anti-METTL14 (Bethyl, A305-847A-M, 1:1000 for Western blots), anti-GAPDH (Proteintech, 60005-1-Ig,1:1000 for Western blots), anti-m^6^A (Synaptic Systems, 202-003, 1:1000 used for MeRIP-Seq or MINT-Seq), anti-Sox1 (Millipore, AB15766, 1:250), anti-Sox2 (R&D systems, MAB2018, 1:200), anti-Nestin (Proteintech, 60004-1-Ig, 1:500).

### Oligonucleotides

See Supplementary information, Table S4.

### siRNA-mediated knockdown

Transfections of siRNAs were performed mostly using Lipofectamine 2000 following the manufacturer’s instructions. RNAiMax was used for transfecting HMC3 and human NPC cells. Often, 5 × 10^5^ cells cultured in a well of 6-well plate were transfected with 40 nM siRNA for qPCR purposes, and two rounds of transfection were often conducted to increase the efficiency of knockdown. Large culture volumes of cells during transfection can be used for RIP or other purposes. A commercial non-targeting control siRNA (Sigma, SIC002) was used as negative control. 48 h to 72 h after transfection, cells were harvested and subjected to RNA extraction, RIP or western blots.

### Establishment of shRNA knockdown stable cell line

In brief, 1.5 μg pLKO shRNA vector with scramble sequences or with sequences targeting specific target genes (purchased from sigma) together with 1 μg psPAX2 and 0.5 μg pMD2.G plasmids were co-transfected into 5 × 10^5^ 293T cells cultured in 6-well plates by using Lipofectamine 3000 (Thermo Fisher) following the manufacturer’s instructions. Virus containing medium was harvested 48 h after transfection. 5 × 10^5^ K562 cells were infected with 1 mL fresh virus medium supplemented with polybrene at a final concentration of 8 μg/mL in 6-well plates. 1 mL fresh RPMI-1640 culturing medium was added to each well 12 h after infection. Selection was performed with 1.5 μg/mL puromycin 24 h after infection and maintained for 3 days. Survived K562 cells were used for experiments, or frozen stocked, or kept cultured in normal medium supplemented with 1 μg/mL puromycin.

### CRISPR/cas9-mediated L1 genetic deletion and inversion

For intronic L1 KO in K562 cells, two sgRNAs targeting L1 flanking sequences were designed using CHOPCHOP,^137^ and were cloned into pSpCas9(BB)-2A-GFP (Addgene 48138) and pSpCas9(BB)-2A-Puro (Addgene 48139) backbones, respectively. Plasmids were prepared with E.Z.N.A.^®^ Plasmid Midi Kit (Omega). Wild-type K562 cells were electroporated with both sgRNA plasmids at 1:1 ratio using Gene Pulser Xcell™ Electroporation Systems (Biorad) following the manufacturer’s instructions. Transfected cells were recovered in the fresh RPMI medium for 1 day and selected by 1.5 μg/mL puromycin for 3 days (to select Addgene 48139 transfected cells). These cells were then sorted by FACS with a BD FACSAria II system (BD Biosciences) to select GFP^+^ cells (indicating Addgene 48138 transfected). Sorted cells were plated into 96-well plates at a density of 1 cell per well and expanded. The genotypes of these isogenic cell clones were validated by PCR assays. Validated cell clones with Super-MIL inversion or homozygous KO were expanded for experiments. SgRNA and primer sequences used are listed in Supplementary information, Table S4.

### CRISPRi

To generate K562 TRE-dCas9-KRAB stable line, 1.5 μg of pHAGE-TRE-dCas9-KRAB (a gift from Rene Maehr & Scot Wolfe, Addgene #50917)^138^ together with 1 μg psPAX2 and 0.5 μg pMD2.G plasmids were co-transfected into 5 × 10^5^ 293T cultured in a well of 6-well plate using Lipofectamine 3000 (Thermo Fisher). Virus was harvested after 48 h and used to infect K562 cells supplemented with polybrene. 1 mL fresh RPMI-1640 culturing medium as added to each well 2 h after infection. Selection was performed with 50 μg/mL G418 48 h after infection and maintained for 6 days before subsequent experiments.

To suppress *PSMA1* gene promoter, two sgRNAs targeting *PSMA1* promoter sequences were designed using CHOPCHOP,^137^ and were cloned into lenti sgRNA(MS2)-zeo backbone (a gift from Feng Zhang, Addgene plasmid # 61427).^139^ SgRNA sequences used are listed in Supplementary information, Table S4. SgRNAs targeting *PSMA1* promoter or a non-targeting control were transfected into 293T cells together with psPAX2 and pMD2.G for lenti-virus generation (see above). Virus was harvested after 48 h and used to infect K562 TRE-dCas9-KRAB stable line supplemented with polybrene. Infected cells were selected with 400 μg/mL zeocin for 7 days and then cultured in medium supplemented with 2 μg/mL doxycycline. Three days later cells were harvested for RNA extraction and RT-qPCR quantification.

### L1-Neo Reporters

The L1HS consensus sequence is derived from EF06R (a gift from Eline Luning Prak, Addgene plasmid # 42940).^140^ To make L1HS-neo-Con reporter, L1HS consensus coding sequences were cloned into L1-neo-TET backbone (a gift from Astrid Roy-Engel, Addgene # 51284, containing a codon-optimized L1)^141^ by replacing the codon-optimized L1 sequence using Gibson assembly. To make L1HS RRACH mutation reporter (L1HS-neo-RRACH-Mut), we disrupted the “RAC” sequences in the middle of 49 RRACH motifs without affecting coding sequence or significant changing codon usage efficiency.^142^ This mutant L1HS sequence was fully synthesized (sequence see Supplementary information, Table S4) and was cloned into L1-neo-TET backbone with Gibson assembly.

### L1 retrotransposition reporter assay

L1 retrotransposition reporter assay was performed according to Kopera et al.^143^ with modification. In brief, 5 million HeLa cells were transfected with siRNAs targeting desired proteins to achieve high knockdown efficiency in retrotransposition time window. 24 h later, cells were co-transfected with L1-reporter constructs (see the previous section) and a puromycin-resistant construct serving as an internal control (Addgene 48139). Two days later, transfected cells were splitted at the same ratio for each group to 6-well plates for G418 selection (400 μg/mL) or puromycin selection (2 μg/mL). To count the numbers of cell colonies with successful L1 retrotransposition, G418 selection was maintained for ~ 14 days, and cells were fixed with a fixation buffer (2% formaldehyde, 0.2% glutaraldehyde in PBS) for 1 h with gentle shaking at room temperature. Fixed cells were washed with ddH2O and stained with 0.1% crystal violet blue for 1 h with shaking at room temperature. Stained cells were rinsed with ddH_2_O and images were taken with a Bio-Rad V3 imaging system. The foci in each plate/condition were counted with ImageJ with the same settings.

### RNA extraction and RT-PCR

Total cellular RNA was extracted with Quick-RNA Miniprep Kit (Zymo Research, #11-328) or TRIzol following the manufacturer’s instructions. Reverse transcription was performed using Superscript™ IV kit (Thermo Fisher, #18091050) using oligodT for MILs, mRNAs or random hexamer for 18S RNA, and U1 as specified in each experiment. SYBR-qPCR was performed using SsoAdvanced™ Universal SYBR^®^ Green Supermix (Bio-Rad, 172-5274) following standard parameters recommended by the manufacturer. All primer sequences are shown in Supplementary information, Table S4.

### Northern blot

Northern blot was performed using DIG Northern Starter Kit (Roche, 12039672910) following the manufacturer’s instructions. In brief, RNA was separated with 1.2% formaldehyde agarose gel and transferred to a positively charged nylon membrane (Roche) by capillary transfer overnight with standard 2× SSC solution. Membrane was UV crosslinked with 400 millijoule (mJ)/cm^2^. Membrane was hybridized with denatured DIG-labeled RNA probe overnight at 68 °C. Strand specific DIG labeled RNA probe was generated with MEGAscript™ T7 Kit (Thermo Fisher). DNA template for probe synthesis was generated by primers specifically targeting the first 100 bp of reporter L1 region, which is shared by both L1 consensus and RRACH mutation reporter (Sequences see Supplementary information, Table S4). T7 promoter sequence was added to the beginning of the reverse primer. Hybridized nylon membrane was washed with four rounds of stringent washes (with 0.1% SDS). Membrane was then blocked, washed and imaged following manufacturer’s instructions.

### dCas13d (dCasRx)-FTO mediated Super-MIL m^6^A demethylation

pLenti-dCasRx-FTO-HA was generated by cloning the coding sequence of human FTO into the backbone of a published construct pLenti-dCAS9-VP64_Blast (Addgene #61425), but with dCas9-Vp64 replaced by dCasRx (which is from Addgene #109050),^103^ all of which were based on gibson cloning. FTO enzymatically-dead mutant (H231A/D233A, FTO-mut), was PCR-amplified from a plasmid kindly provided by Dr. Chuan He lab as a gift,^93^ which was introduced to the backbone of pLenti-dCAS9-VP64_Blast (Addgene #61425, but with dCas9-VP64 replaced by dCasRx) in the same manner as the wildtype FTO. Stable cell lines expressing dCasRx-FTO or dCasRx-FTO-mut were generated by infection with lentivirus packaged with helper constructs and were selected with blasticidin. Guide RNAs for Cas13d (or CasRx) system were designed following a published algorithm,^144^ and were cloned into a lentiviral backbone (pLentiRNAGuide_002-hU6-RfxCas13d-DR-BsmBI-EFS-Puro-WPRE, Addgene # 138151). Sequences of gRNAs were listed in the Supplementary information, Table S4. Cells expressing either dCasRx-FTO or dCasRx-FTO-mut were infected with either the Non-targeting (NT) or the specific gRNA lentiviruses, and after selection with puromycin for 3 days, cells were harvested for experiments.

### UV-RIP

UV-RIP was performed according to Jeon and Lee^145^ with modifications. Briefly, 10 millions of HeLa cells per RIP were UV-crosslinked at 254 nm (400 mJ/cm^2^) in 10 mL ice-cold PBS and collected by scraping. Cells were incubated in a cold lysis buffer (0.5% NP-40, 0.5% sodium deoxycholate, 20 unit (U)/mL Superase-In, 1× Protease Inhibitor in PBS) at 4 °C for 25 min with rotation. Lysate was sonicated with Qsonica Q800R3 sonicator for 5 min at 25% amplitude with 10/20 s ON/OFF cycle followed by 10 U/mL Turbo DNase treatment for 15 min at 37 °C. After centrifugation, the supernatant was incubated with 2 μg Rabbit IgG or anti-YTHDC1 antibodies immobilized on 20 μl Dynabeads™ Protein G beads overnight at 4 °C. Next morning, protein G beads bound with RNA–protein complexes were washed three times with 1 mL ice-cold wash buffer (1% NP-40, 150 mM NaCl, 0.5% sodium deoxycholate, 20 U/mL Superase-In, 1× Protease Inhibitor in PBS), and treated with 10 U Turbo DNase for 30 min at 37 °C. After three more times of wash with wash buffer supplemented with 10 mM EDTA, beads were digested with 200 μL Proteinase K buffer (100 mM Tris-HCl, 50 mM NaCl, 10 mM EDTA, 0.5% SDS and 500 μg/mL Protease K) for 30 min at 65 °C. RNA was recovered by TRIzol reagent and examined by RT-qPCR.

### MTS TT-seq and MINT-seq

Nascent RNA labeling and capture was performed according to Duffy et al.^146^ with modifications. 1 × 10^8^ cells were cultured in normal medium supplemented with 700 uM 4SU (Sigma T4509) for 5 min at 37 °C. These cells were immediately lysed by TRIzol (Thermo Fisher) after medium removal. Total RNA was extracted following the manufacturer’s instructions and was dissolved in the biotinylation buffer (10 mM HEPES-KOH pH7.5, 1 mM EDTA) to a concentration of 0.4 μg/μL. Biotinylation of nascent RNA was done by adding 1/4 volume of MTSEA biotin-XX (Biotium, 90066-1, 166 μg/mL, dissolved in DMF, Sigma, 227056) followed by rotating in dark for 2 h. RNA was extracted with phenol/chloroform (acidic, pH 4.5) according to standard precipitation protocol. Purified RNA dissolved in the fragmentation buffer (10 mM ZnCl_2_, 10 mM Tris-HCl pH7.4) was fragmented at 70 °C for 15 min to an average size of 100–200 nt. Fragmentation was terminated by adding 1/10 volume of 0.5 M EDTA on ice. An aliquot of fragmented total RNA was transferred into a new tube and was subjected to RNA-Seq and MeRIP-Seq if desired. Other fragmented RNA was diluted with the high-salt buffer (50 mM Tris-HCl, pH 7.4, 1 M NaCl, 10 mM EDTA, 0.05% Tween-20, 20 U/mL Superase-In) by at least 5 folds. 50 μL pre-balanced streptavidin C1 beads (Thermo Fisher, 65002) were added and the mixture was incubated at room temperature for 30 min with rotation. The beads were washed 3 times with 700 μL high-salt buffer, twice with 700 μL TET buffer (10 mM Tris-HCl, pH 7.4, 1 mM EDTA, 0.05% Tween-20), once with 1 mL TE buffer (10 mM Tris-HCl, pH 7.4, 1 mM EDTA), with 2 min rotation at room temperature for each wash. Nascent RNA was eluted twice by 150 μL fresh-made 5% beta mercaptoethanol (b-ME) with rotation for 15 mins at dark. The two elutes were combined and precipitated with iso-propanol. Eluted RNA was dissolved in 1010 μL of ice-cold IP buffer (10 mM Tris-HCl, pH 7.4, 150 mM NaCl, 0.1% NP-40, 20 U/mL Superase-In, 1× Protease Inhibitor). 10 μL of dissolved nascent RNA was transferred to a new tube and was used for TT-Seq (also serves as 1% of MINT-seq input). The remaining 1000 μL will be used for m^6^A immunoprecipitation. m^6^A-spike-in mixture (see in vitro RNA transcription) was added to both input and m^6^A IP samples. In the meantime, anti-m^6^A antibody conjugated Dynabeads™ Protein G beads (Thermo Fisher, 10004D) were prepared by adding anti-m^6^A antibody to 20 μL pre-balanced beads in 1 mL IP buffer followed by incubation at room temperature for 30 min. The beads (i.e., beads with m^6^A antibody) were then washed 3 times with the IP buffer and mixed with the RNA samples for IP. The mixture of RNA and beads was incubated at 4 °C for at least 4 h with rotation. The beads were washed 3 times with ice-cold IP buffer, twice with ice-cold low-salt wash buffer (10 mM Tris-HCl, pH 7.4, 50 mM NaCl, 0.1% NP-40, 20 U/mL Superase-In, 1× Protease Inhibitor), twice with ice-cold high-salt wash buffer (10 mM Tris-HCl, pH 7.4, 500 mM NaCl, 0.1% NP-40, 20 U/mL Superase-In, 1× Protease Inhibitor), once with ice-cold TE buffer, with 5 min rotation at 4 °C for each wash. The same amount of ERCC-RNA spike-in (Thermo Fisher, 4456740) was added to both washed beads and the input RNAs. Immunoprecipitated RNAs and input RNAs were extracted by TRIzol-LS (Thermo Fisher) following the manufacturer’s instructions. Both immunoprecipitated and input RNAs were subjected to next generation sequencing library preparation, which are MINT-seq and TT-seq samples, respectively.

### RNA-Seq and MeRIP-Seq

Fragmented total RNA (see MTS-TT-seq and MINT-seq) was diluted with ice-cold IP buffer (10 mM Tris-HCl, pH 7.4, 150 mM NaCl, 0.1% NP-40, 20 U/mL Superase-In, 1× Protease Inhibitor) to a final volume of 1010 μL. 10 μL of diluted RNA was transferred to a new tube and was used for RNA-seq (also serves as 1% of MeRIP-seq) and kept on ice. The remaining 1000 μL will be used for m^6^A immunoprecipitation. m^6^A-spike-in mixture (see in vitro RNA transcription) was added to both input and IP samples. In the meantime, anti-m^6^A antibody conjugated Dynabeads™ Protein G beads were prepared by adding anti-m^6^A antibody to 20 μL pre-balanced beads in 1 mL IP buffer followed by incubation at room temperature for 30 min. The beads were washed 3 times with the IP buffer and added to the RNA samples for IP. The mixture of RNA and beads was incubated at 4 °C for at least 4 h with rotation. The beads were washed 3 times with ice-cold IP buffer, twice with ice-cold low-salt wash buffer (10 mM Tris-HCl, pH 7.4, 50 mM NaCl, 0.1% NP-40, 20 U/mL Superase-In, 1× Protease Inhibitor), twice with ice-cold high-salt wash buffer (10 mM Tris-HCl, pH 7.4, 500 mM NaCl, 0.1% NP-40, 20 U/mL Superase-In, 1× Protease Inhibitor), once with ice-cold TE buffer, with 5 min rotation at 4 °C for each wash. ERCC-RNA spike-in (Thermo Fisher) was added to both washed beads and the input RNAs previously prepared. Immunoprecipitated RNAs and input RNAs were extracted by TRIzol-LS following the manufacturer’s instructions. Both immunoprecipitated and input RNAs were subject to next generation sequencing library preparation, which are MeRIP-seq and RNA-seq, respectively.

### Library preparation and next generation sequencing

We used ribo-depleted total RNA for most of the RNA-sequencing in this paper. Only for a few cases, we used polyA^+^ RNA for sequencing, and we labeled them in the legends (e.g., Figs. 6b, 7). 5–200 ng RNA was used for each library preparation using NEBNext Ultra II Directional Library Prep Kit for Illumina (NEB, E7760) following the manufacturer’s instructions. Ribosome RNA was depleted with NEBNext rRNA Depletion Kit (NEB, E6301). For Poly-A RNA-Seq, the total RNAs were selected by NEBNext^®^ Poly(A) mRNA Magnetic Isolation Module (NEB, E7490) and the resulting polyA RNAs were used for library making in the same way as above described. Generated libraries in this study were mostly sequenced using NextSeq 550 Sequencing System with paired-end 40nt/40nt mode following the manufacturer’s instructions.

### Western blots

Protein samples were prepared by adding 2× Laemmli sample buffer (Bio-Rad, 1610737) to cell pellets followed by passing through QIA shredder (QIAGEN, 79656). Denatured proteins were separated by 4%–15% Mini-Protean TGX SDS-PAGE gels (BioRad, 4561096) and transferred to PVDF membranes. Membranes were blocked with 5% milk in the TBST buffer (10 mM Tris-HCl, pH 7.4, 150 mM NaCl, 0.1% tween-20) followed by incubation with primary antibodies at 4 °C overnight. Membranes were washed 3 times with TBST buffer and then incubated with anti-mouse/rabbit secondary antibodies conjugated to HRP (Jackson) for 30 min at room temperature. After 3 times TBST buffer wash, proteins on the PVDF membrane were detected by chemiluminescence using Clarity™ Western ECL Substrate (Biorad, 1705060) and a ChemiDoc™ Gel Imaging System according to manufacturer’s instructions.

### In vitro RNA transcription

We made m^6^A-labeled mixed RNA species with known m^6^A levels as spike-in controls in our MINT-seq. Twenty non-human template sequences at 200–300 bp in length were generated by PCR with T7 containing primers (see Supplementary information, Table S4). These template DNAs were divided into 5 non-redundant groups with 4 sequences in each group. For each group, template DNAs were mixed and transcribed with MEGAscript™ T7 Transcription Kit (Invitrogen, AM1334) with ATP or ATP/m^6^ATP mix (1:1) to generate non-methylated RNAs or m^6^A-methylated RNAs, respectively. Non-methylated RNAs and m^6^A-methylated RNAs generated from the same group were mixed with the following ratios (1:0, 3:1, 1:1, 1:3 or 0:1 for groups 1–5, respectively), which generates spike-in groups with different methylation levels (0%, 25%, 50%, 75% and 100%). Equal amounts from the five RNA spike-in groups were combined and mixed as final m^6^A-spike-in RNAs.

For in vitro RNA pulldown assay in Fig. 3 and Supplementary information, Fig. S6, templates of L1HS, control sequence and L1 fragments were generated by PCR from the EF06R plasmid (Addgene #42940), L1-neo-TET (Addgene #51284) and human genomic DNA with primers containing T7 polymerase binding sequences (see Supplementary information, Table S4), respectively. For in vitro RNA pulldown assay in Supplementary information, Fig. S6h, templates of L1HS, PSMA1 Super-MIL and a non-L1 intron region from PSMA1 gene were generated by PCR from human genomic DNA with T7 containing primers (see Supplementary information, Table S4). These template DNAs were in vitro transcribed using MEGAscript™ T7 Transcription Kit (Invitrogen, AM1334) with extra biotin-11-UTP (1.5 mM final concentration, 20% of all UTP in this reaction) and *N*^6^-methyl-ATP (3 mM final concentration, 40% of all ATP in this reaction), or biotin-11-UTP only to generate biotin-labeled m^6^A-RNAs or biotin-labeled RNAs. In vitro transcribed RNAs were examined by formaldehyde RNA agarose gel to verify theirpurify, size and abundance.

### Generation of recombinant SAFB protein

Briefly, the protein coding region of human SAFB was amplified from K562 cDNA (primers see Supplementary information, Table S4) and cloned into a pFastBac His6 TEV LIC cloning vector (4B) (a gift from Scott Gradia, Addgene #30115) with a 6× His tag at the N-terminus. The resulting plasmid was transformed into DH10Bac competent cells (ThermoScientific). The recombinant bacmid plasmid was used for transfection of Sf9 insect cells. High-titer baculoviruses obtained after three rounds of viral amplification were used for infection of High Five cells (ThermoScientific). 72 h post infection, High Five cells were harvested and then lysed by dounce homogenizer. Lysates were clarified by high-speed centrifugation at 18,000 rpm for 30 min. The supernatant was subjected to immobilized metal affinity chromatography purification using for Ni-NTA resin (ThermoScientific) at 4 °C. The eluted protein was then loaded into a heparin column (HiTrap HP, ThermoScientific) equilibrated in buffer A (20 mM HEPES pH 7.0, 50 mM KCl, 5 mM MgCl_2_, and 10% glycerol). The SAFB protein was eluted in a linear gradient over 10 column volumes with buffer B (buffer A containing 1 M KCl). The elutes were examined by SDS-PAGE. The fractions that contain pure SAFB protein were combined for subsequent in vitro pulldown experiments.

### In vitro biotinylated RNA pulldown

In vitro RNA pulldown assay was performed according to a published work Tsai et al.^147^ 10 μg in vitro synthesized, biotin or biotin/m^6^A labeled RNAs in 20 μL DEPC treated water was denatured at 65 °C for 3 min and then put on ice. RNA samples were diluted with 130 μL RNA structure buffer (10 mM Tris-HCl, pH 7.4, 100 mM KCl, 10 mM MgCl_2_, 50 U/mL Superase-In) and then incubated at room temperature for 20 min. 800 μL High-salt buffer (50 mM Tris-HCl, pH 7.4, 1 M NaCl, 10 mM EDTA, 0.05% Tween-20, 20 U/mL Superase-In) and 50 μL pre-balanced streptavidin C1 beads were added to RNA samples. The mixtures were incubated at room temperature for 1 h and then washed 3 times with the high-salt buffer.

For pulldown against cell lysates, 2 ×  10^7^ K562 cells were lysed with 1 mL RIPA buffer (Thermo Fisher, supplemented with 20 U/mL Superase-In and 1× Protease inhibitor) and then centrifuged at 12,000× for 10 min at  °C. The supernatant was added to the prepared RNA-bound C1 beads. For pulldown against recombinant proteins, protein solution was diluted with RIPA buffer for at least ten folds before added to the prepared RNA-bound C1 beads. The mixture was then incubated at 4 °C for 4 h with rotation. Beads were washed twice with RIPA buffer, twice with high salt wash buffer (10 mM Tris-HCl, pH 7.4, 500 mM NaCl, 0.1% NP-40, 20 U/mL Superase-In), with 5 min of rotation at 4 °C for each wash. Washed beads were directly boiled in sample buffer and subjected to western blots or coomassie blue staining for target protein detection.

### In vitro RNA competition assay to test SAFB and L1 binding

To immobilize SAFB-L1 complex on streptavidin beads, we first bind biotinylated L1HS RNA to the beads. 250 ng biotin-labeled L1HS RNA (see in vitro RNA transcription) was denatured at 65 °C for 3 min, chilled on ice, diluted with 130 μL RNA structure buffer (10 mM Tris-HCl, pH 7.4, 100 mM KCl, 10 mM MgCl2, 50 U/mL Superase-In) and then incubated at room temperature for 20 min. 800 μL High-salt buffer (50 mM Tris-HCl, pH 7.4, 1 M NaCl, 10 mM EDTA, 0.05% Tween-20, 20 U/mL Superase-In) and 50 μL pre-balanced streptavidin C1 beads were then added to the RNA samples. After incubation at room temperature for 1 h, the RNA-beads complex was washed 3 times with the high-salt buffer, and then incubated with 2 μg recombinant SAFB proteins in RIPA buffer (Thermo Fisher, supplemented with 20 U/mL Superase-In and 1× Protease inhibitor) at 4 °C for 4 h with rotation. Beads bound with L1HS RNA and SAFB were washed twice with RIPA buffer with 5 min of rotation at 4 °C for each wash. The immobilized L1HS-SAFB complex was then incubated with various amounts of synthesized competition RNA at 4 °C for more than 4 h with rotation. After that, beads were washed twice with RIPA buffer, twice with high salt wash buffer (10 mM Tris-HCl, pH 7.4, 500 mM NaCl, 0.1% NP-40, 20 U/mL Superase-In), with 5 min of rotation at 4 °C for each wash. Washed beads were directly boiled in sample buffer and were subjected to western blots or coomassie blue staining for target protein detection.

### Genomic L1HS measurement by qPCR

K562 cells stably expressing shRNA targeting mRNA of SAFB were continuously cultured for 20–30 passages in full medium or supplemented with 10 μM 3TC (2′-3′-dideoxy-3′-thiacytidine, Sigma). Genomic DNA was extracted by Quick-DNA Miniprep Kit (ZYMO research) and sheared by sonication using Qsonica Q800R3 sonicator for 5 min at 25% amplitude with 10 s/20 s ON/OFF cycle. Same amount of fragmented genomic DNA was subjected to qPCR with SsoAdvanced™ Universal SYBR® Green Supermix (Biorad, 172-5274) to examine the relative copy number of L1HS or L1PA2.

### Generation of iPSC-derived hNPCs

Human NPCs were generated from human iPSCs using a modified dual SMAD inhibitors method.^148^ Human iPSCs were reprogrammed from the dermal fibroblasts from a male patient using Sendai Virus kit (CytoTune Sendai Reprogramming Kit) containing transcription factors OSKM (OCT4, SOX2, KLF4, C-MYC following manufacturer’s instruction. Reprogrammed iPSCs were digested into small clumps using 0.5 mM EDTA, and were then transferred to petri dishes and suspended as embryoid bodies (EBs) in human iPSC media (minus bFGF) supplemented with 10 mM SB-431542, 1 mM dorsomorphin, 3 mM CHIR 99021, 0.5 mM purmorphamine, and 10 mM ROCK inhibitor Y-27632 (all from Tocris Bioscience). On day 2, medium was replaced by N2B27 medium supplemented with the same small molecule supplements without ROCK inhibitor. N2B27 medium contains DMEM/F12: Neurobasal (1:1) supplemented with 1× Glutamax, 1× NEAA, 1× N2 and 1× B27 minus Vitamin A (all from Thermo Fisher). On day 4, all the small molecules were withdrawn and the medium was changed to NPC medium, comprising N2B27 medium with 20 ng/mL bFGF. On day 6, EBs were attached to the cell culture dish coated with Matrigel. After 2–4 days, neural rosettes were manually isolated and dissociated into single cells. The cells were expanded in NPC medium and were passaged every 4–5 days in a 1:4–6 ratio.

### Immunocytochemistry

Human NPCs were characterized by immunocytochemistry. Briefly, cells grown on glass coverslips were fixed with 4% paraformaldehyde and incubated in the blocking buffer (5% goat serum, 1% bovine serum albumin, and 0.1% Triton X-100) for 15 min. Cells were then incubated in primary antibodies diluted in the blocking buffer at 4 °C overnight. Appropriate secondary antibodies were used for single and double labeling. All secondary antibodies were tested for cross-reactivity and nonspecific immunoreactivity. The following primary antibodies were used, anti-SOX2 (1:200, R&D Systems), anti-Nestin (1:500, R&D Systems), anti-SOX1 (1:250, Millipore). Bis-benzamide (DAPI, 1:1000; Sigma) was used to visualize the nuclei. Images were captured using a Zeiss Axiovision microscope with z-stack split view function.

### Bioinformatic processing of MINT-Seq, TT-Seq, RNA-Seq and MeRIP-Seq data

MINT-Seq, TT-Seq, MeRIP-Seq raw data were de-multiplexed by bcl2fastq (v2.20), and quality-controlled with fastqc. All clean reads were mapped to human reference genome hg19 by STAR v2.7.0,^149^ with parameters of –-genomeSAindexNbases 14 –outFilterMultimapNmax 10 –outFilterMismatchNmax 10. Duplicated reads were removed, and only unique aligned reads will be considered for later visualization and quantification. For gene/mRNA quantification, hg19 RefSeq gene annotation coordinates were used. HOMER toolset was used to calculate the FPKM value of individual repeat elements, based on repeat mask annotation.^150^ The unique aligned reads were further converted to bigwig format with a fragment length of 75 nt for visualization on IGV browser.

### Quality control for MINT-Seq and TT-Seq based on dual spike-in

The quality control plots for MINT-Seq and TT-Seq are shown in Supplementary information, Fig. S1b, c. The raw sequencing reads of dual-spiked-in identified in MINT-Seq or MeRIP-Seq were aligned to two pre-built genomes (based on the 92 ERCC sequences and our in-house m^6^A spike-in sequences, see Supplementary information, Table S4) with STAR v2.7.0. In order to estimate the pulldown efficiency of m^6^A-spike-in RNAs, we first used ERCC reads to normalize the RNA-Seq reads of both the m^6^A IP group (MINT-Seq) and Input RNA group (TT-Seq). We used a linear regression model (MINT-Seq/TT-Seq ERCC reads) to obtain an ERCC normalization factor. The observed m^6^A level of one transcript will be calculated by raw m^6^A ratio (MINT-Seq/TT-Seq m^6^A spike-in reads) divided by ERCC normalization factor. Further, as the m^6^A levels of the spike-in m^6^A reads are known, we can compare the observed m^6^A levels of spike-in to their expected m^6^A levels by calculating the spearman’s correlation coefficient (Supplementary information, Fig. S1c).

### Quantification of the m^6^A levels of L1 subfamily by TE transcript

Most RTE repeat quantification is based on uniquely aligned reads. We also considered multi-mapped reads to estimate m^6^A level on L1 sub-family by the *TEtranscript* pipeline,^57^ which applied an EM algorithm to handle non-unique mapped reads. The clean reads of MINT-Seq were aligned to the reference genome (hg19) (using STARv2.5.2) with the parameters of “–winAnchorMultimapNmax 100 –outFilterMultimapNmax 100” as recommended by *TEtranscripts*. *TEtranscripts* was run using stranded options (–stranded reverse and –stranded yes).

### Identification of m^6^A peaks, and the annotation of MILs and Super-MILs

We first performed m^6^A peak calling by using MACS2^151^ with the parameters of -f BAM -q 0.01 -n. We are aware that there are alternative tools to call m^6^A peaks,^152,153^ but MACS2 remains one of the commonly used tools and works robustly for many different m^6^A RNA-Seq datasets in different tissues or conditions. For MINT-Seq peak calling, we considered TT-Seq as input, and for MeRIP-Seq peak calling, we treated RNA-Seq as input. The called m^6^A peaks were overlapped with annotated LINE-1 elements using bedtools v2.1.0.^154^ We used HOMER de novo motif discovery tool (findMotifGenome.pl -rna) with MINT-Seq peaks overlapping intronic L1s as input sequences to identify potential m^6^A RNA motifs. Annotation of m^6^A peaks overlapping genomic regions was performed by the HOMER toolset. MILs (m^6^A-methylated intronic L1s) were identified as transcribed intronic L1s (FPKM > 0.1, length > 200 bp) that overlap at least one m^6^A peak (MINT-Seq or MeRIP-Seq). Then the methylation level of each MIL was computed by the ratio between (MINT-Seq FPKM + pseudo-count)/(TT-Seq FPKM + pseudo-count), which was ranked to generate the plot shown in Fig. 1e. Mappability-adjusted FPKM values and pseudo-count were applied to ensure that the results are not biased towards low reads coverage of L1s. The Super-MILs were identified in a similar way as the method used to find Super-enhancers,^51^ i.e., we find the data point that shows a slope closest to 1 in the ranked plot of m^6^A methylation levels (e.g., Fig. 1e). MILs ranked higher than that point were considered as Super-MILs.

### Evolution related analysis of L1s

This analysis is related to Supplementary information, Fig. S6d. We followed a recent work that conducted evolutionary estimates of L1 ages,^155^ in which L1s specific to the primate or the euarchontoglires lineages were considered young L1, while L1s present in cows and dogs were considered old L1s. For motif analyses, the L1 subfamily sequences were retrieved from Dfam database (https://dfam.org/), and ages of different L1 subfamilies were based on a previous publication.^55^

### De novo transcript assembly

The ENCODE K562 poly-A RNA-Seq (Supplementary information, Table S4) datasets were used to perform de novo transcript assembly for identifying MILs splicing or their derived transcripts in a genome-wide manner. Briefly, the BAM files were first sorted with samtools,^156^ then a de novo transcript assembly tool, *Stringtie*^72^ was applied to analyze the data with these parameters (-p 64 -f 0.5 -m 200 -a 10 -j 3 -c 2 -g 200 –rf). To identify de novo transcripts that contain MILs, we overlapped the StringTie de novo assembly transcripts with K562 MILs identified from MINT-Seq datasets using bedtools (Supplementary information, Fig. S5).

### Analysis of ENCODE eCLIP-Seq datasets

K562 ENCODE eCLIP-Seq raw datasets were downloaded from ENCODE data portal (see Supplementary information, Table S5). All clean-reads were mapped to human reference genome hg19 by STAR v2.7.0, with parameters of –genomeSAindexNbases 14 –outFilterMultimapNmax 10 –outFilterMismatchNmax 10. Duplicated reads were removed, and only unique aligned reads were considered for visualization and quantification (except for Supplementary information, Fig. S3a that used both unique and non-unique mappable reads). For gene/mRNA quantification, hg19 RefSeq gene annotation coordinates were used. HOMER tool-sets were used to calculate the FPKM values of individual repeat elements, based on repeat mask annotation. The unique aligned reads were further converted to bigwig format with a fragment length of 75 bp to visualize on IGV browser. For SAFB eCLIP motif analyses in Supplementary information, Fig. S6g, top 1000 CLIP binding sites (from ENCFF639MAG, from ENCODE data portal) located to L1s were used and each binding sites were extended 15 base pairs as input sequences for motif discovery by GraphProt. For the distribution of eCLIP binding sites on L1HS consensus sequences, we largely followed the procedure of a previous method.^67^ Briefly, eCLIP reads were mapped to the hg19 reference genome with STAR (parameters: --winAnchorMultimapNmax 200 --outFilterMultimapNmax 100 --outFileNamePrefix $3 --alignEndsType EndToEnd --alignEndsProtrude 100 DiscordantPair). The UMI information of eCLIP reads were utilized to remove possible duplicates with ENCODE eCLIP tool: barcode_collapse_pe.py (https://www.encodeproject.org/software/barcode_collapse_pe.py/). We further extracted reads that can be aligned to genome annotated L1HS regions in hg19 reference genome, filtered these reads with clipping, insertion, and deletions (to ensure the reads are not from other L1 sub-families), and aligned the filtered reads (read2 only) to the L1HS consensus sequence with bwa-mem (default parameters). The distribution of eCLIP binding sites on L1HS were calculated with bedtools (genomeCoverageBed -strand + -3 -d).

### MILs in human fetal tissues

The human fetal tissue MeRIP-Seq and RNA-Seq datasets were retrieved from a previous study.^112^ All MeRIP and RNA-Seq reads were mapped to human reference genome hg19 by STAR v2.7.0, with parameters of –genomeSAindexNbases 14 –outFilterMultimapNmax 10 –outFilterMismatchNmax 10. Duplicated reads were removed, and only unique aligned reads will be considered for visualization and quantification. Any intronic LINE-1 that overlapped with an m^6^A peak (based on multiple replicates of MeRIP-seq datasets) in a specific human fetal tissue was considered an MIL. The RefSeq genes that harbor at least one MIL were used to perform functional enrichment analysis using Metascape^157^).

### TBI

TBI was calculated utilizing TT-Seq reads coverage. Upstream TT-Seq signal denotes the reads coverage (FPKM) between a specific intronic L1s’ 5' end and the hosting gene TSS, while downstream TT-Seq signal denotes reads coverage (FPKM) between an intronic L1s’ 3-end and the hosting gene TES. Then TBI for each intronic L1 were calculated as the ratio between the upstream TT-Seq FPKM and the downstream TT-Seq FPKM. Only sense-oriented (relative to host gene) intronic L1s were considered when we calculated the TBI values. MIL-hosting genes were excluded from Control L1-hosting genes (i.e., if a gene is a MIL-host, then it was no longer counted as Control L1 host genes even if it does have Control L1 in its introns). For PSMA1 Super-MIL TBI calculation, the TES of PSMA1 gene were manually annotated to exclude the reads from PSMA1 gene short isoforms.

### Host gene annotation and information

MIL-hosting genes are RefSeq genes (hg19) that harbor identified MILs in their introns (regardless of orientation, although MILs are predominantly sense to genes). The list of DDR genes was generated by combining genes included in two GO terms (cellular response to DNA damage stimulus (GO: 0006974), and DNA repair (GO: 0006281), Supplementary information, Table S6). A list of neuronal/synaptic genes was generated based on combining genes included in any GO terms containing “neural/neuro” or “nerv” or “synap” (Supplementary information, Table S7). L1 suppressor genes were defined based on two recent genome-wide screens.^11,63^ In Liu et al.^63^ (K562 CRISPR screen), any genes that showed CasTLE score < 0 (i.e., repressor) in their high-coverage secondary screen targeting the high-confident hits (150 genes, FDR < 0.3) were considered as L1 suppressor genes in our current work. Mita, et al., identified 220 inhibitory hits (Z-score > 3.0) in their RNAi screen, and all were considered as L1 suppressor genes in our current analysis. Genes associated with the Autism spectrum disorders (ASD) were obtained from the SFARI database (https://www.sfari.org/resource/sfari-gene/).

All qPCR data were analyzed with R and were presented as means ± SD as indicated. Two-tailed Student’s *t*-test was used to compare means between groups as indicated; *P* < 0.05 was considered significant, and *P-*values are indicated in each figure. All other statistical analyses were performed with Python script. Key software used in analysis of high-throughput sequencing data are described above.

## Supplementary information

Supplementary Fig 1

Supplementary Fig 2

Supplementary Fig 3

Supplementary Fig 4

Supplementary Fig 5

Supplementary Fig 6

Supplementary Fig 7

Supplementary Fig 8

Supplementary Fig 9

Supplementary Fig 10

Supplementary Fig 11

Supplementary Fig 12

Table S1

Table S2

Table S3

Table S4

Table S5

Table S6

Table S7

## Data Availability

All data generated in this study have been deposited to GEO database GSE137752. Other published datasets used in this work have been listed in Supplementary information, Table S5. Custom scripts used for the analysis have been deposited to GitHub (https://github.com/wblilab-uth/MIL-scripts).
