# Supplementary material for: RNA m6A modification orchestrates a LINE-1–host interaction that facilitates retrotransposition and contributes to long gene vulnerability
Source: Cell Res. 2021 Jun 9;31(8):861–85. doi: 10.1038/s41422-021-00515-8 (PMC8324889; doi:10.1038/s41422-021-00515-8)
Supplement: Supplementary file 1 — Supplementary Fig 1 [file 41422_2021_515_MOESM1_ESM.pdf]

Supplementary information, Fig. S1

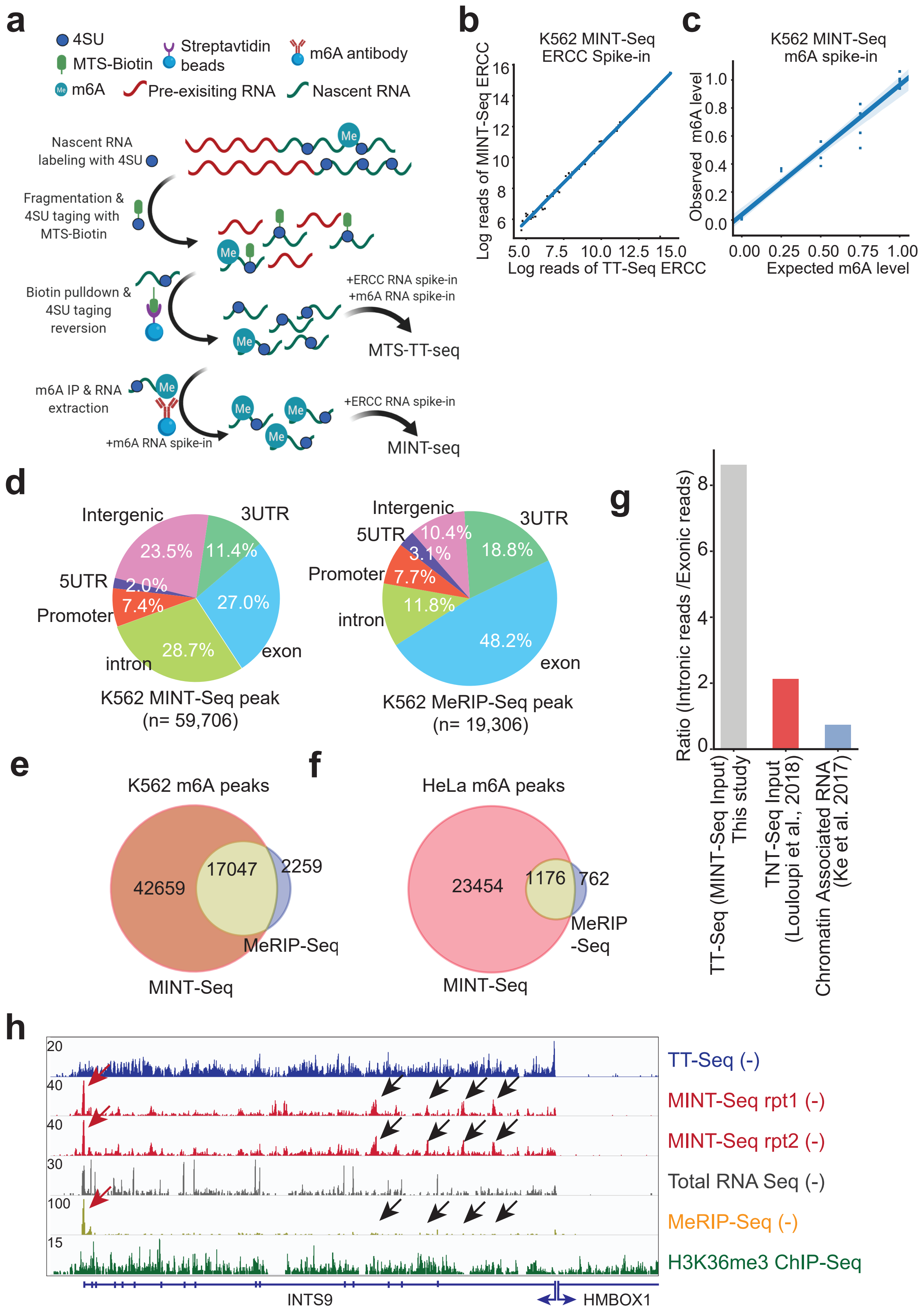

Supplementary information, Fig. S1 | The establishment of MINT-Seq and the global landscapes of nascent RNA m^{6}A methylome.

a) A diagram describing the workflow of MTS TT-Seq and MINT-Seq methods.

b, c) Correlation plots for the dual Spike-in controls used in MINT-Seq and TT-seq. In the B panel, a scatter plot shows a high concordance of raw read counts of in total 92 ERCC spike-in sequences detected in one example of K562 TT-Seq (x-axis) and MINT-Seq (y-axis). In the C panel, this is a scatter plot showing the m^{6}A levels of synthesized m^{6}A spike-in non-human RNA sequences based on their known m^{6}A ratios during synthesis (x-axis) as compared to their observed m^{6}A levels from this set of MINT-Seq and TT-Seq. Linear regression (blue lines) was performed to calculate the regression factor.

d) Pie charts depicting the genomic distributions of m^{6}A peaks identified from K562 MINT-Seq (left panel) and MeRIP-Seq (right panel).

e-f) Venn diagrams showing the numbers of m^{6}A peaks by MINT-Seq versus m^{6}A peaks by MeRIP-Seq in K562 **(e)** or HeLa cells **(f)**.

g) A bar plot comparing the ratios between intronic reads and exonic reads detected in three different studies on nascent or chromatin-associated RNAs (MINT-Seq by this study, TNT-Seq Input by Louloupi et al. Cell Reports, 23, 3429–3437, 2018; Chromatin-associated RNA by Ke et al. Genes & Dev. 31, 990–1006; 2017).

h) A genome browser track of K562 TT-Seq, MINT-Seq, ribo-depleted RNA-Seq and MeRIP-Seq, as well as H3K36me3 ChIP-Seq (ENCODE) at the INTS9 gene loci. The black arrows indicate some nascent RNA m^{6}A peaks that can only be detected by MINT-Seq, while the red arrows point to 3'UTR peaks that exist in both MINT-Seq and MeRIP-Seq.
