# Supplementary material for: RNA m6A modification orchestrates a LINE-1–host interaction that facilitates retrotransposition and contributes to long gene vulnerability
Source: Cell Res. 2021 Jun 9;31(8):861–85. doi: 10.1038/s41422-021-00515-8 (PMC8324889; doi:10.1038/s41422-021-00515-8)
Supplement: Supplementary file 2 — Supplementary Fig 2 [file 41422_2021_515_MOESM2_ESM.pdf]

Supplementary information, Fig. S2

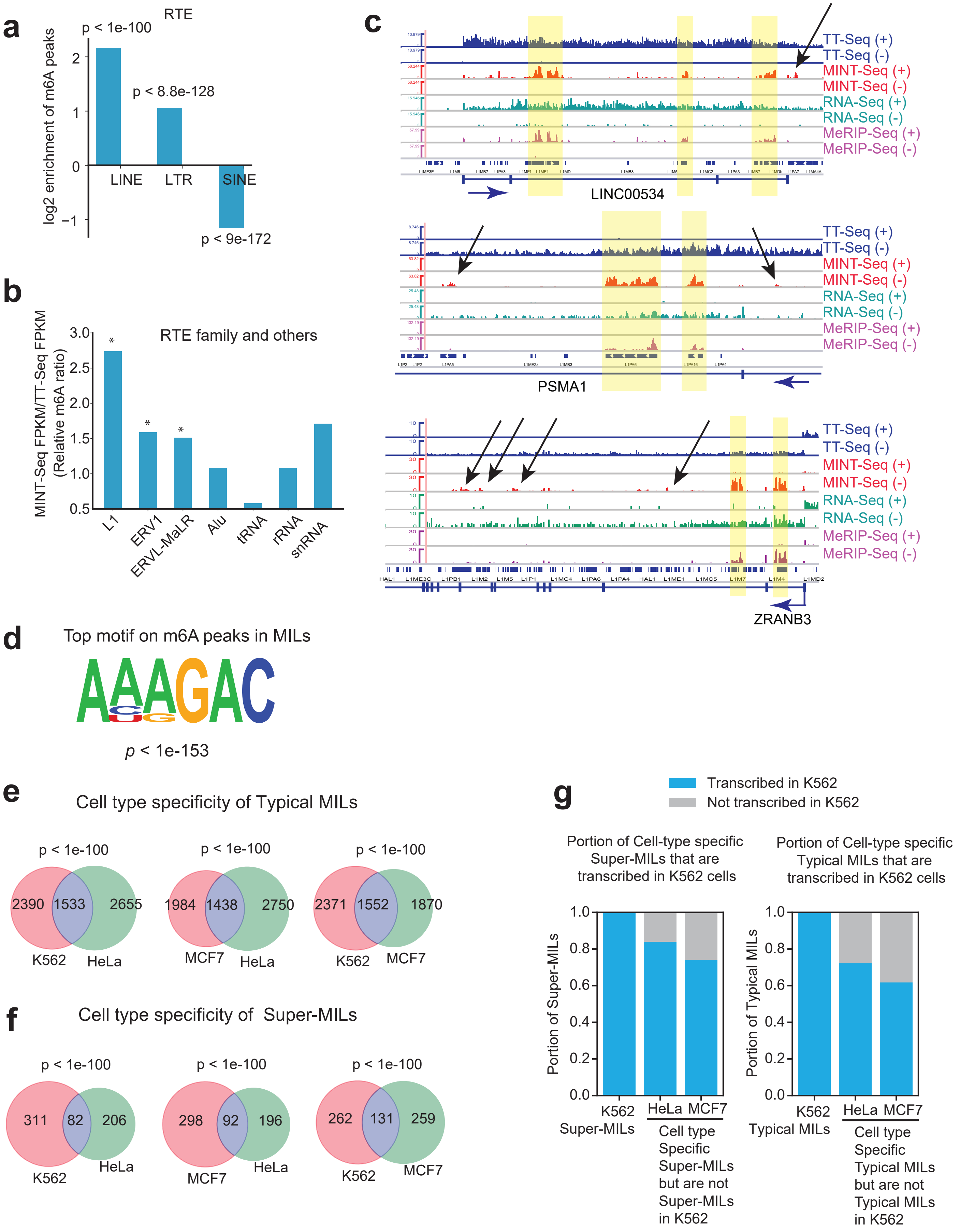

Supplementary information, Fig. S2 | m^{6}A levels of retrotransposons and L1s, their motifs and MIL landscapes.

a) A bar plot showing the log2 enrichment (observed/expected) of MINT-Seq peaks on the LINE, LTR, and SINE elements (based on Fig. 1a). P-values indicate significant enrichment over expectation, which were calculated with Fisher's exact tests and labeled on top of each bar.

b) A bar plot showing the relative m^{6}A levels (MINT-Seq/TT-Seq) of different types of RTEs or other noncoding RNAs that are not RTEs. Here, all annotated regions of each RTE category were considered. tRNA, rRNA and snRNA are included for comparison. The ratio indicates the enrichment of MINT-seq signal to each category. * : $p < 1e-100$, Fisher's exact test.

c) Snapshots of the genome browser tracks of K562 TT-Seq, MINT-Seq, RNA-Seq, MeRIP-Seq at LINC00534 (upper), PSMA1 (middle), and ZRANB3 (lower) loci. Yellow highlights show peaks of Super-MILs. The arrows indicate the m^{6}A peaks (mostly are MILs) that can only be well-identified from MINT-Seq.

d) Logo of the top m^{6}A motif identified by HOMER from intronic L1 m^{6}A peaks showing a motif consistent with the known one: RRACH (where R = A/G, and H = A/C/U). P-value was calculated by HOMER [153].

e-f) Venn diagrams showing the overlap of typical MILs (**f**) and Super-MILs (**g**) between K562 and MCF7 or HeLa cells. P-values were calculated with hyper-geometric tests.

g) Bar Graphs showing that cell type specific MILs and Super-MILs are often transcribed in other cell types. For this plot, cell type specific Super-MILs or MILs in K562, HeLa or MCF7 were examined as to how many of them are transcribed in K562 cells (K562 TT-Seq FPKM > 0.1). More than 60% of HeLa- or MCF7- specific MILs or Super-MILs are transcribed in K562 cells, but are not identified as MILs or Super-MILs in K562. This suggests that cell type specificity of MILs is not solely due to cell type specific transcription, although the latter contributes to the former.
