# Supplementary material for: RNA m6A modification orchestrates a LINE-1–host interaction that facilitates retrotransposition and contributes to long gene vulnerability
Source: Cell Res. 2021 Jun 9;31(8):861–85. doi: 10.1038/s41422-021-00515-8 (PMC8324889; doi:10.1038/s41422-021-00515-8)
Supplement: Supplementary file 3 — Supplementary Fig 3 [file 41422_2021_515_MOESM3_ESM.pdf]

Supplementary information, Fig. S3

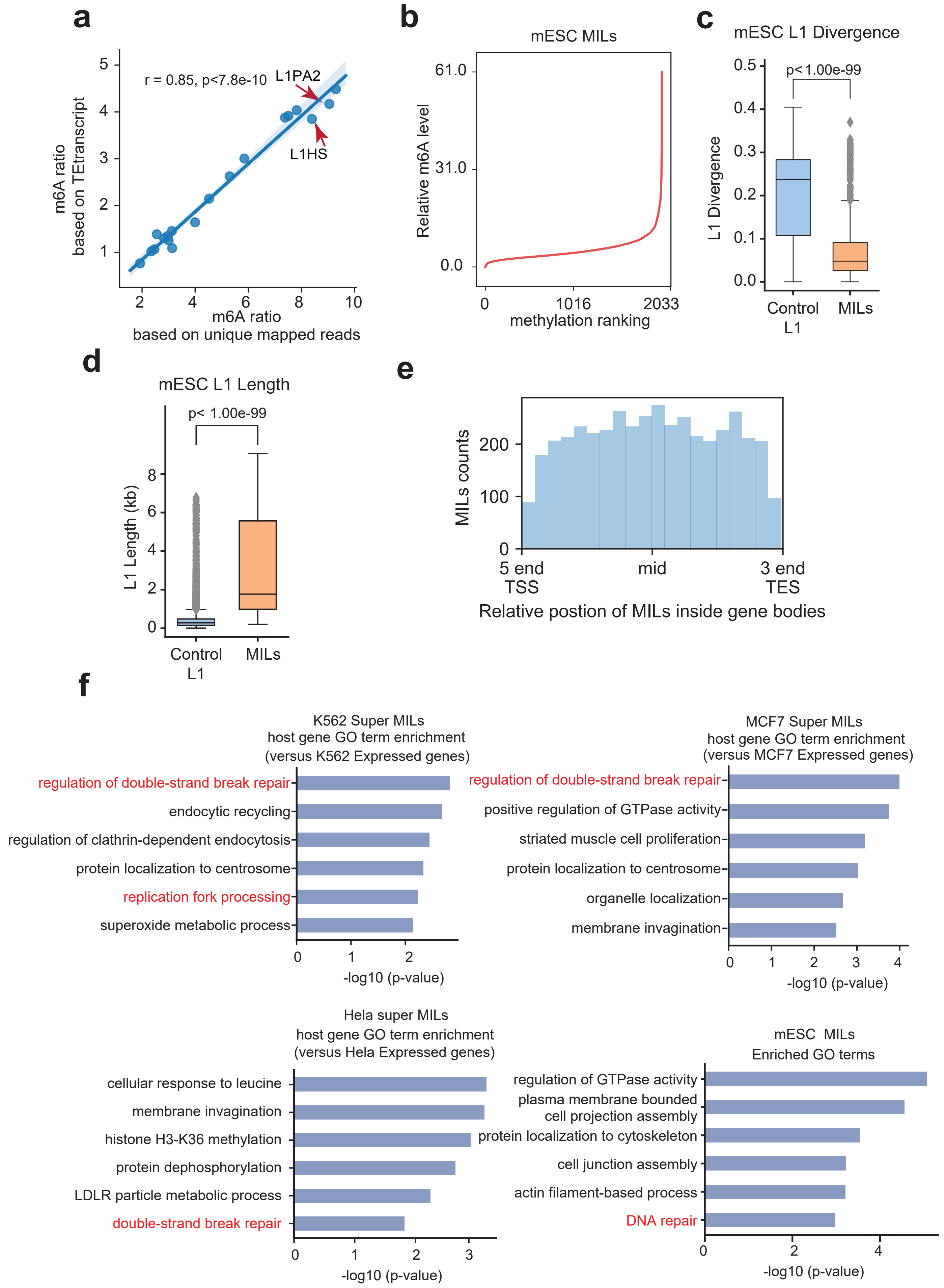

Supplementary information, Fig. S3 | MILs are conserved in mouse ESCs and enriched in DNA damage repair genes

a) A scatter plot showing the m^6A ratio on different L1 sub-families calculated based on uniquely mapped reads (x-axis) or based on the *TEtranscript* pipeline (including non-unique aligned reads). Data are from K562 MINT-Seq and TT-Seq (their division generates m^6A ratio). Correlation coefficient and p-value were calculated with Spearman correlation.

b) Similar to Fig. 1e, a distribution plot of relative m^6A level (using mESC nuclear RNA MeRIP-Seq FPKM/ nuclear RNA-Seq FPKM) across murine MILs.

c-d) A boxplot showing features of random selected transcribed L1s (Control L1) or MILs in mouse ESC, including their L1 sequence divergence (**c,** DNA sequence differences relative to the L1 consensus sequences) and length (**d**). P-values were calculated with Mann-Whitney U test.

e) The relative locations of MILs in the host genes. This histogram indicates the number (Y axis) of all MILs plotted against its location in different sections (20 bins) of their host genes. TSS: transcription start sites; TES: transcription end sites.

f) The top functional enrichment of genes that host Super-MILs in K562 cells (top-left panel), in MCF-7 cells (top-right panel), in HeLa cells (bottom left panel), and in mouse ESCs (bottom right panel). Terms of DNA damage repair were shared by all cell types and are thus highlighted. All the enrichment analysis was conducted by using the genes expressed in that cell type (FPKM>0.1) as the "background".
