# Supplementary material for: RNA m6A modification orchestrates a LINE-1–host interaction that facilitates retrotransposition and contributes to long gene vulnerability
Source: Cell Res. 2021 Jun 9;31(8):861–85. doi: 10.1038/s41422-021-00515-8 (PMC8324889; doi:10.1038/s41422-021-00515-8)
Supplement: Supplementary file 4 — Supplementary Fig 4 [file 41422_2021_515_MOESM4_ESM.pdf]

Supplementary information, Fig. S4

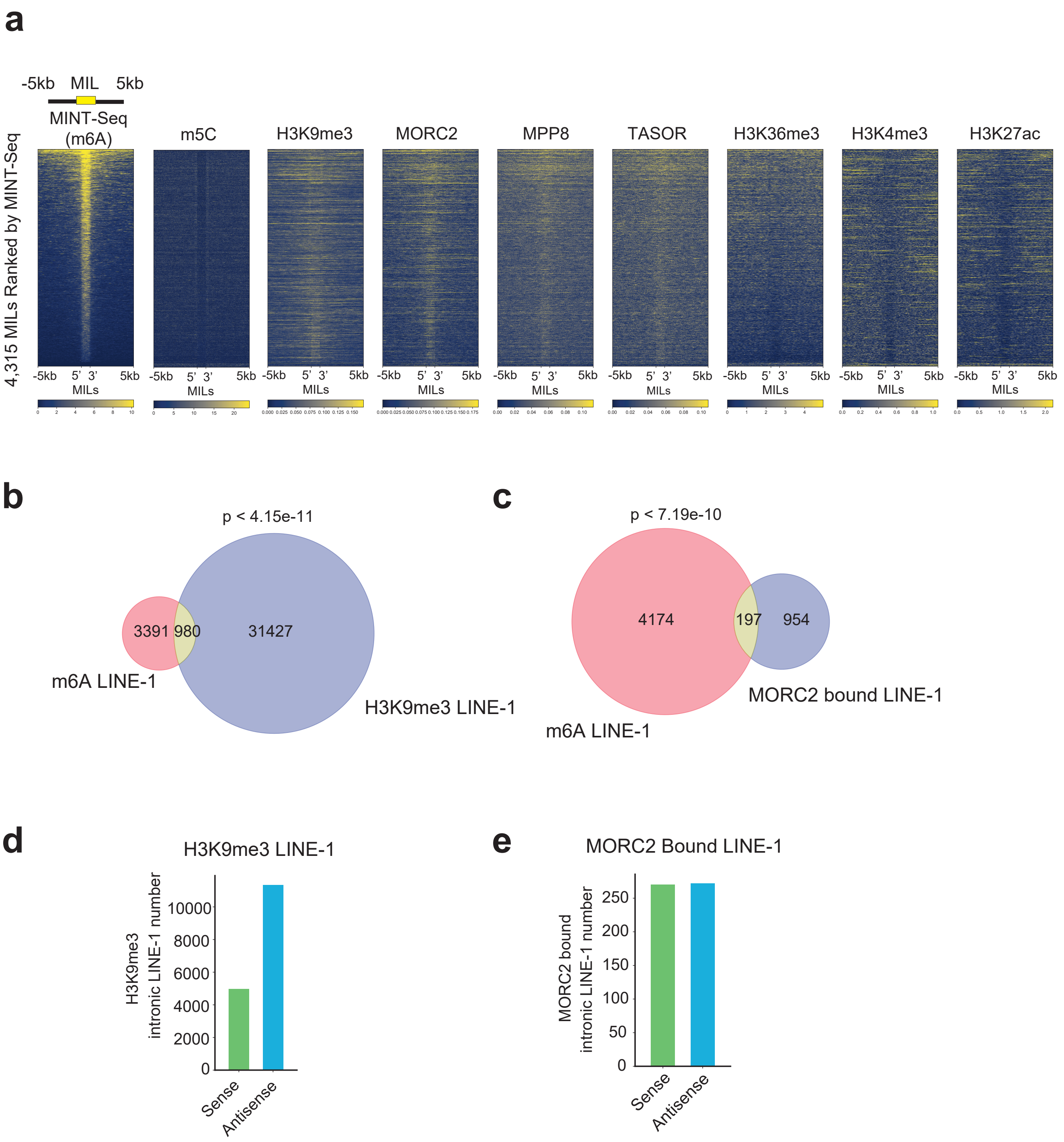

Supplementary information, Fig. S4 | m^{6}A-marked L1s show limited overlap with those marked by H3K9me3 or HUSH complex

a) MIL-centered (+/- 5kb) heatmaps showing the normalized signals of MINT-Seq, DNA 5C methylation (WGBS), ChIP-Seq of H3K9me3, HUSH complex (MORC2, MPP8 and TASOR) and other histone marks (H3K36me3, H3K4me3, H3K27ac) in K562 cells. The heatmaps are all vertically ranked based on MINT-Seq signals.

b-c) Venn-diagrams showing overlaps between intronic L1s with MINT-Seq m^{6}A peaks and L1s with H3K9me3 ChIP-Seq peaks **(b)** or L1s with MORC2 ChIP-Seq peaks **(c)**. P-values were calculated with hyper-geometric tests.

d-e) Barplots showing the observed numbers of sense- and antisense- (relative to host gene direction) orientated H3K9me3-marked **(d)** or MORC2 bound **(e)** intronic L1s.
