# Supplementary material for: RNA m6A modification orchestrates a LINE-1–host interaction that facilitates retrotransposition and contributes to long gene vulnerability
Source: Cell Res. 2021 Jun 9;31(8):861–85. doi: 10.1038/s41422-021-00515-8 (PMC8324889; doi:10.1038/s41422-021-00515-8)
Supplement: Supplementary file 5 — Supplementary Fig 5 [file 41422_2021_515_MOESM5_ESM.pdf]

Supplementary information, Fig. S5

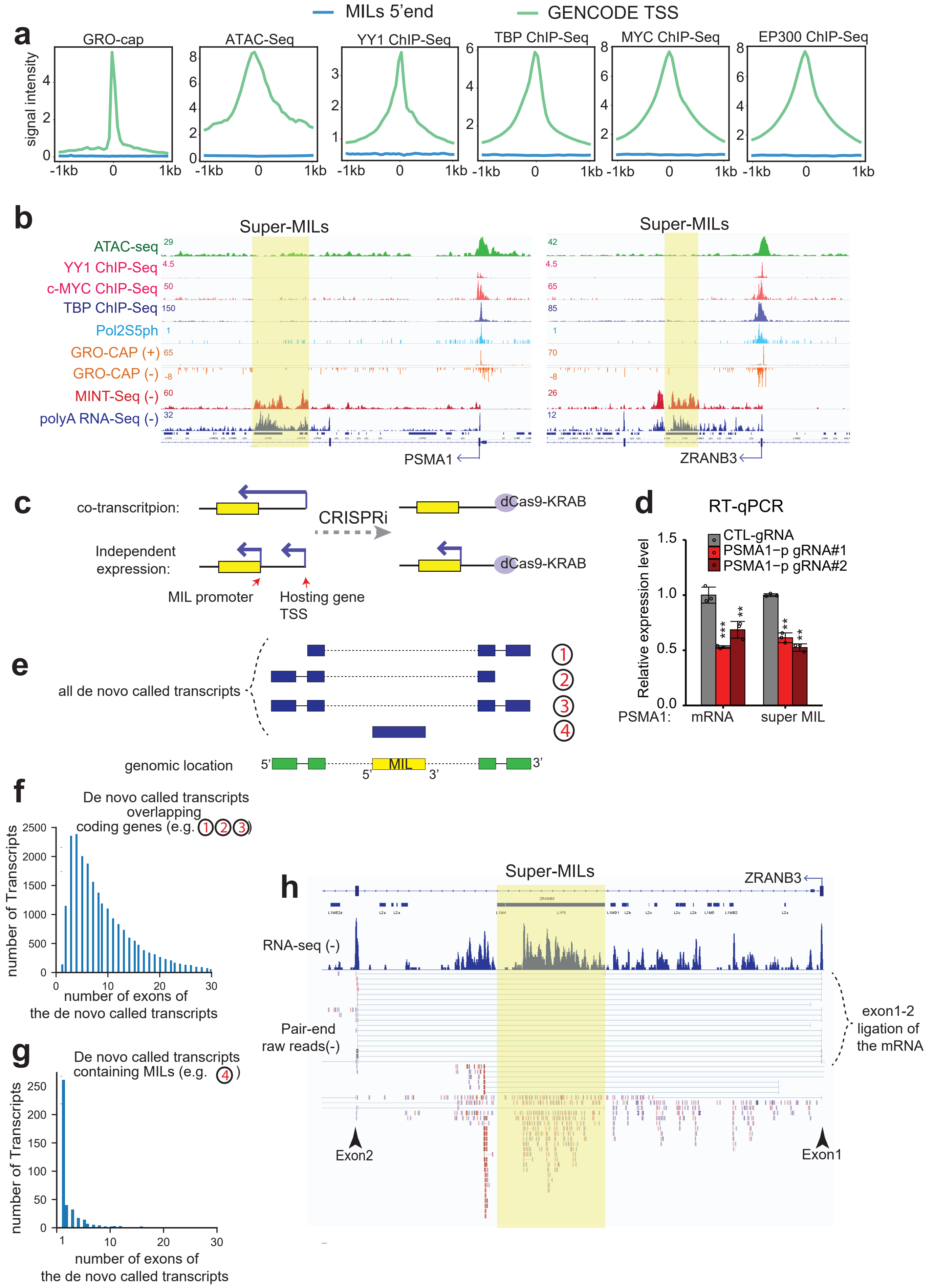

Supplementary information, Fig. S5 | MILs are not produced by their independent promoters and are largely not spliced to host gene mRNAs

a) Profile plots showing the lacking of active transcription initiation signals, including GRO-CAP, ATAC-Seq and multiple ChIP-Seqs, on MILs 5'ends (blue signals) as compared to regular gene transcription start sites (GENCODE TSSs, green signals).

b) Snapshots of IGV genome browser showing that some full-length MILs (two Super-MILs in the introns of PSMA1 and ZRANB3, also see Fig. 5) are highly expressed based on poly-A RNA-Seq, with levels that are comparable to, or even higher than the host gene levels. But they do not show signs of independent promoters, i.e. lack of signals in ATAC-Seq, GRO-CAP, and no binding of several transcription factors or ser-5 phosphorylated RNA polymerase II (Pol2S5ph). These features contrast that of normal gene TSSs, or that of autonomously active L1HS promoter (e.g. **Fig. 2e**).

c) A diagram showing that the two models of MIL transcription (e.g. co-transcription based on the hosting gene promoter or independent transcription based on its own promoter) respond differently to CRISPRi suppression of the host gene promoter. The right side shows the two possible consequences when the hosting gene promoter is inhibited.

d) RT-qPCR results showing the reduction of both PSMA1 mRNA and the Super-MIL after CRISPRi inhibition of PSMA1 gene promoter with two separate guide RNAs (#1 and #2). A scramble gRNA (CTL-gRNA) was used as a control. Data show mean +/- SD. **, $p < 0.01$; ***, $p < 0.001$, Student's t-test.

e) A diagram for the next two panels **f** and **g** showing our strategy to use *de novo* transcript calling to test how often MILs are spliced into mRNA exons. The circled numbers 1,2,3,4 are four examples of *de novo* called transcripts. We then examined the transcripts overlapping with RefSeq mRNAs or with MILs. For example, for the circled number 4, if a *de novo* transcript overlaps MILs, and has only one exon, then this indicates that it is not spliced into neighboring exons.

f, g). Histograms showing the distribution of exon numbers for the *de novo* transcripts that either overlap mRNAs or MILs. **f:** *de novo* transcripts that overlap RefSeq mRNA exons are often multi-exonic (which is expected). **g:** *de novo* transcripts that overlap MILs are generally single-exonic, suggesting that MILs are often not spliced with any neighboring exons.

h) Pair-end BAM raw reads of poly-A RNA-Seq showing that the reads over the ZRANB3 Super-MIL predominantly come from its independent RNA. Reads that span distant regions indicate potential splicing events, which are very common in between the exons 1 and 2 of the gene mRNA (indicated by black arrows and the dotted line bracket to the right).
