# Supplementary material for: RNA m6A modification orchestrates a LINE-1–host interaction that facilitates retrotransposition and contributes to long gene vulnerability
Source: Cell Res. 2021 Jun 9;31(8):861–85. doi: 10.1038/s41422-021-00515-8 (PMC8324889; doi:10.1038/s41422-021-00515-8)
Supplement: Supplementary file 6 — Supplementary Fig 6 [file 41422_2021_515_MOESM6_ESM.pdf]

Supplementary information, Fig. S6

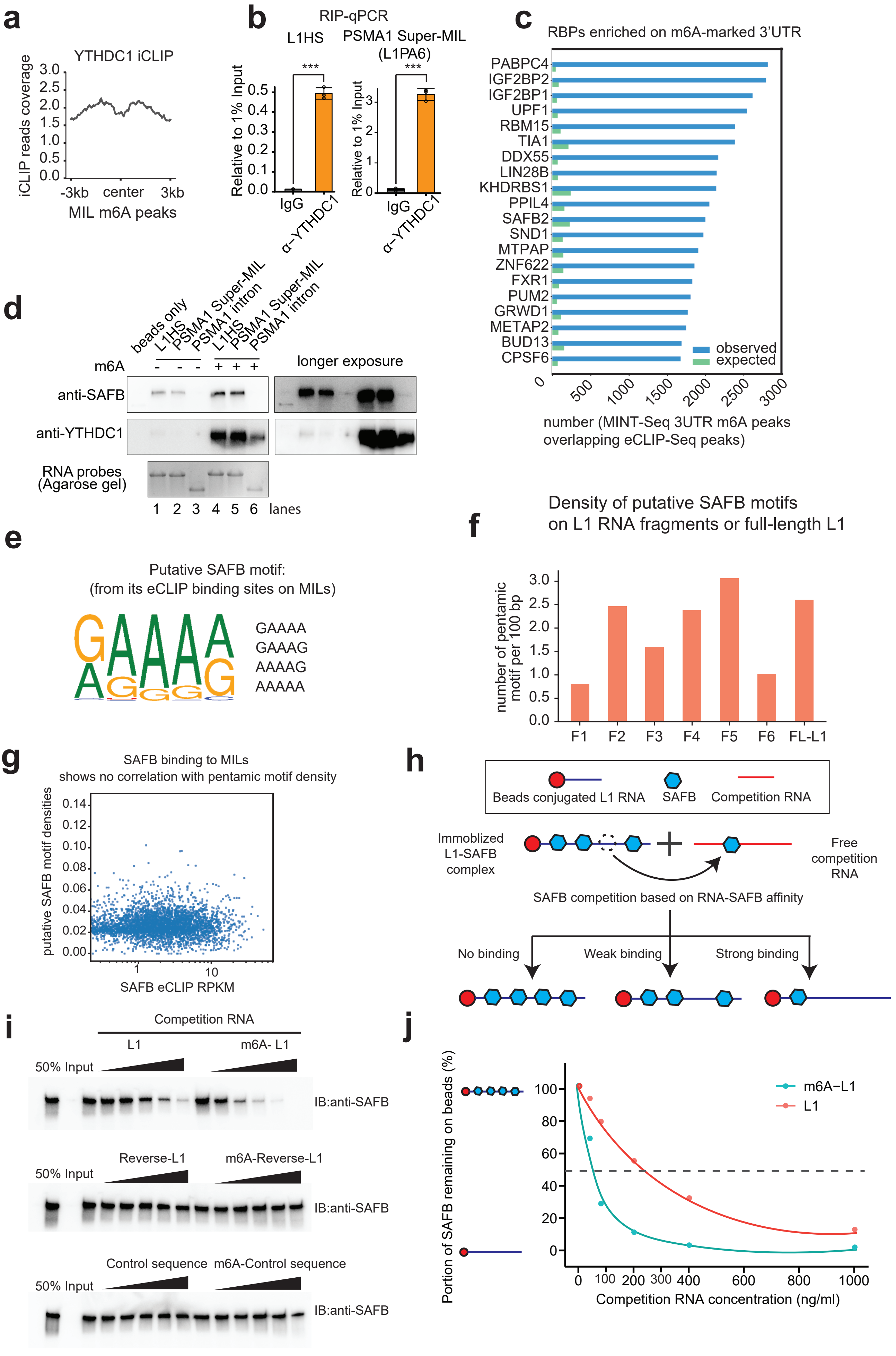

Supplementary information, Fig. S6 | RBPs binding to MILs and 3'UTRs, and additional characterization of SAFB and MIL binding.

a) A profile plot showing YTHDC1 iCLIP read densities in 293T cells centered on intronic m^6A peaks in L1s (i.e., MILs).

b) YTHDC1 UV-RIP-qPCR showing its binding with L1HS and a Super-MIL in the PSMA1 intron.

c) A ranked bar plot showing the numbers of m^6A peaks in gene 3'UTRs that overlap with RBP eCLIP peaks (ENCODE K562 datasets). The blue bars indicate observed m^6A peak numbers, and the green bars are expected numbers calculated using randomly shuffled regions. p-values were calculated with Fisher's exact tests and all are highly significant ($p < 1e-100$), so are not labeled.

d) Western blots following in vitro RNA pull down showing binding between *in vitro* synthesized biotinylated RNAs (with or without m^6A) and SAFB or YTHDC1.

e) The putative SAFB motif identified from the top 1000 SAFB eCLIP peaks locating to L1s, by using the *GraphProt* algorithm. The right side shows the four possible A/G rich pentamer motifs based on the left-side logo.

f) A bar plot showing the densities (number per 100bp) of the four putative SAFB motifs (shown in e panel) occurring on the six L1HS fragments or the full-length L1HS (FL-L1).

g) A scatterplot showing no correlation between putative pentamic motif densities (numbers of the four pentamers per bp on each MIL) and the observed SAFB binding (eCLIP FPKM on each MIL). Each dot represents a MIL. Spearman's correlation coefficient is shown.

h) A scheme of in vitro RNA competition assay. Pre-bound SAFB to biotinylated L1HS RNAs were immobilized on streptavidin beads (red dots). A variable amount of competition RNAs were used to compete with the pre-bound L1HS RNA to examine their affinity binding SAFB protein, which can be determined by the remaining amount of SAFB on beads.

i-j) The relative binding affinity of SAFB to different RNA species analysed by competition assay. For each group, the competitor RNA of six different concentrations (0, 50, 100, 200, 400, 1000 ng/ml) was used. The remaining SAFB protein on beads was determined by western blots (**i**) and the relative affinity curve of L1 and m^6A-L1 (**j**) was calculated based on western blot results. All the other control RNAs showed negligible binding with SAFB (**i**). The concentrations of L1 and m^6A-L1 RNAs to compete off 50% of immobilized SAFB are indicated by a dashed line.
