# Supplementary material for: RNA m6A modification orchestrates a LINE-1–host interaction that facilitates retrotransposition and contributes to long gene vulnerability
Source: Cell Res. 2021 Jun 9;31(8):861–85. doi: 10.1038/s41422-021-00515-8 (PMC8324889; doi:10.1038/s41422-021-00515-8)
Supplement: Supplementary file 7 — Supplementary Fig 7 [file 41422_2021_515_MOESM7_ESM.pdf]

Supplementary information, Fig. S7

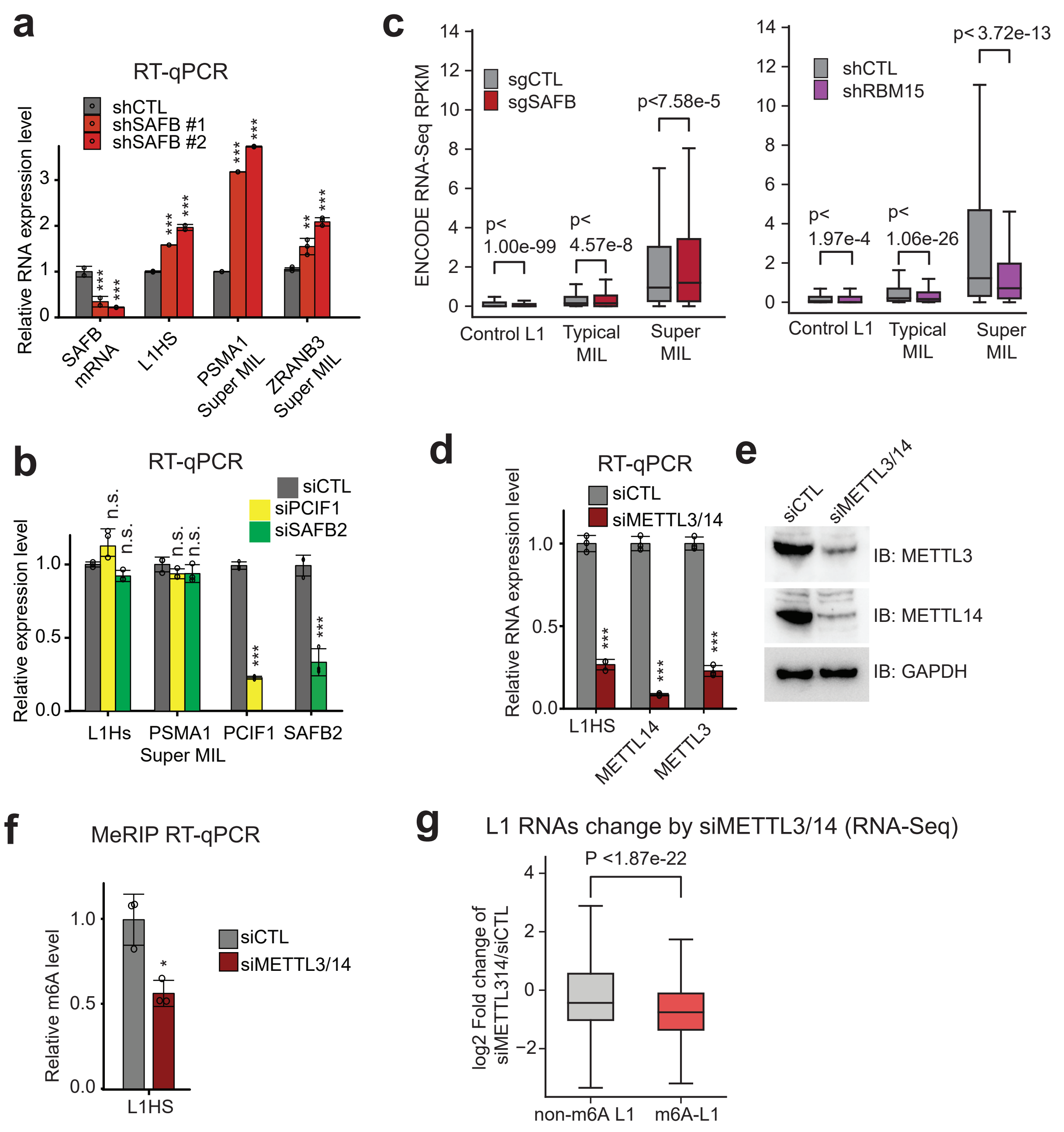

Supplementary information, Fig. S7 | Roles of m^{6}A regulators and SAFB on L1 expression

a) RT-qPCR results showing depletion of SAFB with two separate shRNAs increased the expression of both L1HS and Super-MILs.

b) RT-qPCR showing depletion of PCIF1 or SAFB2 caused negligible effect on L1HS or Super-MIL expression.

c) Boxplots showing the RNA-Seq results after SAFB (left) or RBM15 (right) depletion, which impact the abundances of Control L1 RNAs, Typical MILs, and Super-MILs (RNA-Seq from ENCODE). P-values were calculated with paired Student's t-tests.

d) RT-qPCR data showing that co-depletion of METTL3 and METTL14 (siMETTL3/14) reduced the expression of L1HS.

e) Western blot showing the efficiency of METTL3/METTL14 double knockdown used in D and F, and in Fig. 4b,c.

f) m^{6}A antibody-based RIP (MeRIP) followed by RT-qPCR showing that co-depletion of METTL3 and METTL14 reduced the m^{6}A methylation level of L1HS RNA.

g) Box plot showing the RNA expression changes of m^{6}A and non-m^{6}A intronic L1s after co-depletion of METTL3 and METTL14 determined by RNA-Seq. P value calculated by Mann-Whitney U test.

For all statistical analysis of qPCR, data show mean +/- SD. * $p < 0.05$; **, $p < 0.01$; ***, $p < 0.001$, Student's t-test.
