# Supplementary material for: RNA m6A modification orchestrates a LINE-1–host interaction that facilitates retrotransposition and contributes to long gene vulnerability
Source: Cell Res. 2021 Jun 9;31(8):861–85. doi: 10.1038/s41422-021-00515-8 (PMC8324889; doi:10.1038/s41422-021-00515-8)
Supplement: Supplementary file 8 — Supplementary Fig 8 [file 41422_2021_515_MOESM8_ESM.pdf]

Supplementary information, Fig. S8

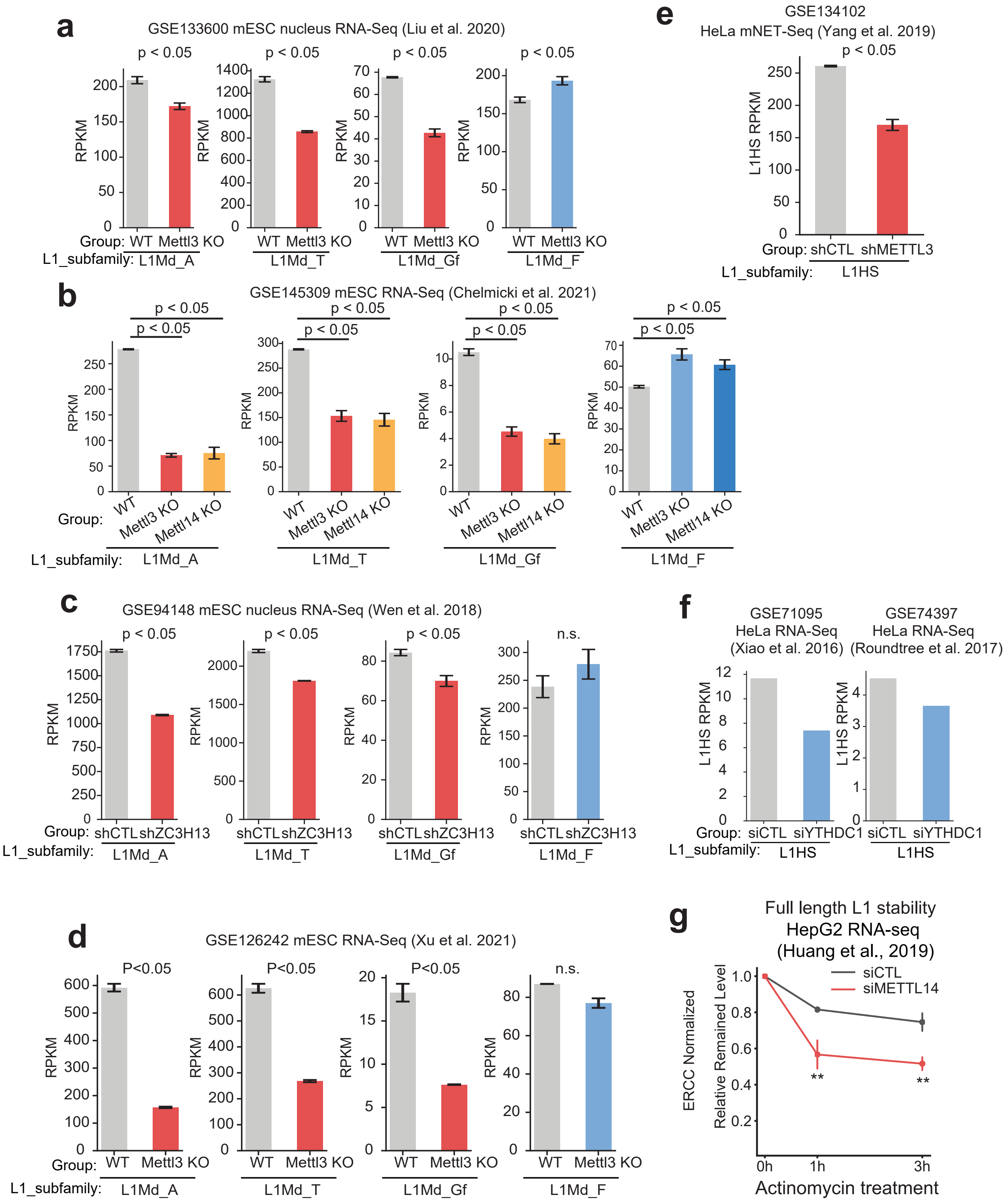

Supplementary information, Fig. S8 | Reanalysis of published data indicates that the expression levels of youngest and retrotranspositionally-active L1 subfamilies RNAs were reduced upon depleting m^{6}A methyltransferases or YTHDC1

a) Barplots showing reduced RNA abundances of the three retrotranspositionally active and evolutionarily young murine L1 sub-families (L1Md_A, L1Md_T, L1Md_Gf) in mESCs after Mettl3 knockout (KO), while the abundance of an older L1 sub-family (L1Md_F) was slightly increased. The L1Md_A and L1Md_T RNAs are also generally of higher abundance (see the following panels b,c as well). The three young L1s are of higher m^{6}A levels (**Fig. 2b**). The plots here were generated by reanalyzing published nucleus RNA-Seq (Liu et al.; GSE133600)[93]. L1Md_A, L1Md_T, and L1Md_Gf are the three major retrotranspositionally active L1s in the mouse genome, and the estimated evolutionary ages of different murine L1s can be found in our **Fig. 2b** or from Sookdeo et al.[59].

b) Barplots showing abundances of the three active murine L1 sub-families and one older L1 sub-family (L1Md_F) in mESCs based on RNA-Seq from a recent work (Chelmicki et al.; GSE145309)[92]. The three groups in each bar plot represent mESCs of wildtype, and of Mettl3 KO or Mettl14 KO, respectively. KO: genetic knockout.

c-d) Similar to those in panel a, barplots showing abundances of three young and active murine L1 sub-families (L1Md_A, L1Md_T, L1Md_Gf) and one older L1 sub-family (L1Md_F) in mESCs after knockdown of the m^{6}A methyltransferase adaptor Zc3h13 (panel **c**) or knockout of Mettl3 (panel **d**). Plots are generated by re-analyzing nucleus RNA-Seq from Wen et al.[58] (GSE94148, panel **c**) and RNA-Seq from Xu et al. [97] (GSE126242, panel **d**).

e) Barplots showing RNA abundances of the active human L1 sub-family (L1HS) in HeLa cells, revealed by mNET-Seq with or without METTL3 knockdown. Data are from Yang et al.[94]; (GSE134102).

f) Barplots showing abundances of the active human L1 sub-family (L1HS) in two sets of HeLa RNA-Seq generated by two different groups with and without knockdown of YTHDC1 (i.e., siCTL, siYTHDC1) (the left one was based on data from Xiao et al.[95]; GSE71095; the right one Roundtree et al.[96]; GSE74397).

g) A line plot showing the RNA stability of full length L1s after transcription inhibitor actinomycin treatment for the indicated time in control or METTL14 knockdown cells (in HepG2 cells)[65]. RNA abundance was calculated based on RNA-Seq and normalized to 0hr time point in each group, by using ERCC spike-ins. P values were calculated by Student's t-test, **, $p< 0.01$.

For all barplots, data show mean +/- SD. P-values in all panels are calculated by Student's t-test from at least two replicates (if the published work included replicates).
