# Supplementary material for: RNA m6A modification orchestrates a LINE-1–host interaction that facilitates retrotransposition and contributes to long gene vulnerability
Source: Cell Res. 2021 Jun 9;31(8):861–85. doi: 10.1038/s41422-021-00515-8 (PMC8324889; doi:10.1038/s41422-021-00515-8)
Supplement: Supplementary file 9 — Supplementary Fig 9 [file 41422_2021_515_MOESM9_ESM.pdf]

Supplementary information, Fig. S9

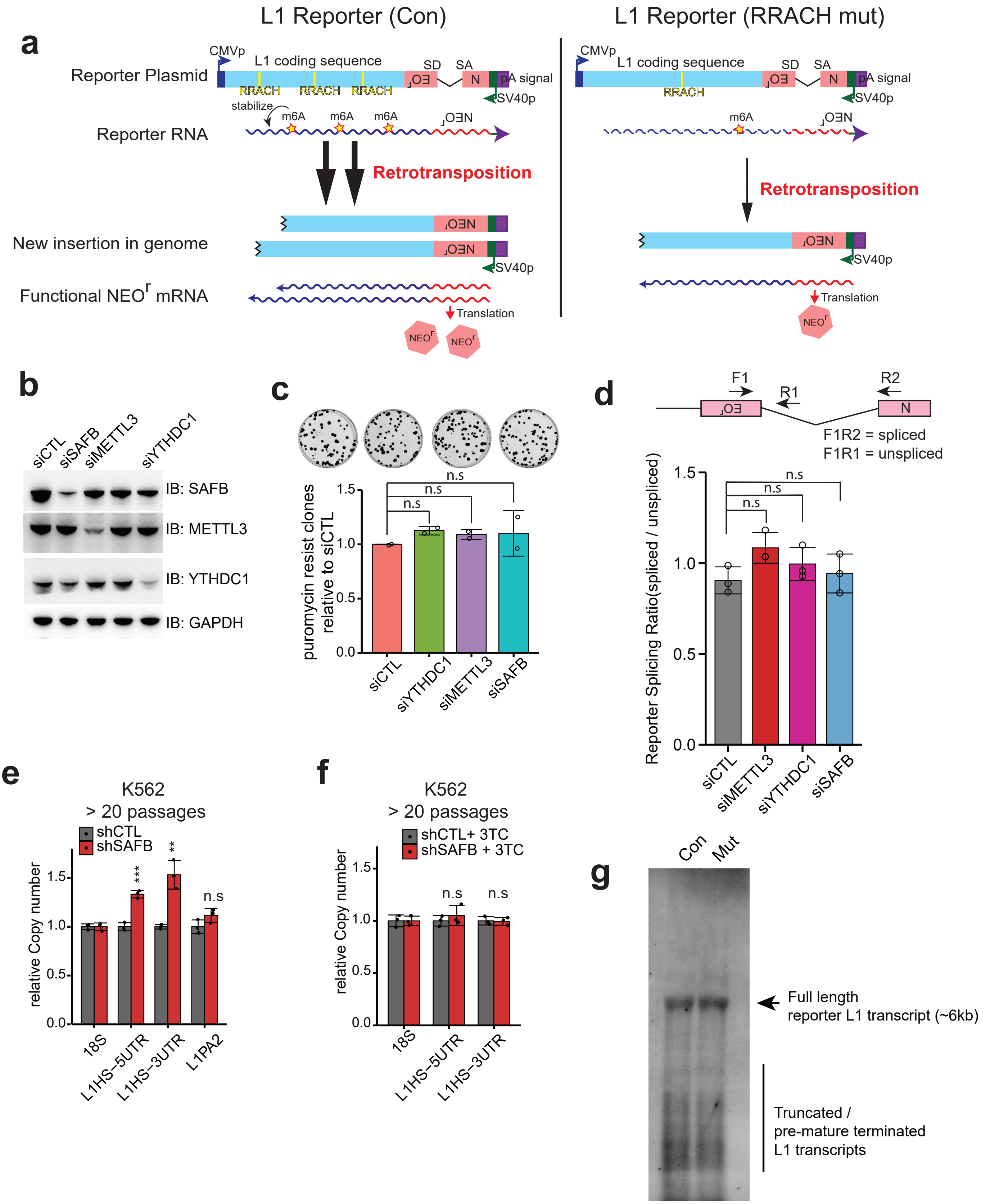

Supplementary information, Fig. S9 | Effects of m^6A and SAFB on L1HS retrotranscription activity

a) A schematic diagram of the retrotransposition assay we used in Fig. 4 that are based on either the L1HS reporter with consensus sequence (Con) or with a RRACH-motif-mutant L1HS reporter (Mut).

b) Western blots showing the knockdown efficiency of the indicated regulators.

c) A control experiment for Fig. 4f (See Material and methods). A puromycin resistant construct was co-transfected with L1-neo reporter, and was subjected to various knockdown; cells were selected with puromycin to ensure any changes of L1 retrotransposition activity is not due to transfection efficiency and/or proliferation rates between groups. Colony numbers were counted and compared to the control group (lower panel). Representative pictures are shown in the upper panel.

d) RT-qPCR showing the splicing ratio of L1 reporter RNA is not affected by depletion of the indicated factors. The splicing ratio was determined by the relative RNA amount of spliced mRNA to unspliced mRNA. Primers used for qPCR were indicated in the upper panel.

e) Genomic DNA qPCR showing that depletion of SAFB in K562 cells for more than 20 passages increased the genomic copy number of L1HS but not that of L1PA2 or 18S DNA.

f) Similar to **e**. Long term depletion of SAFB did not affect the genomic copy number of L1HS when lamivudine/3TC, a well-established inhibition of reverse transcriptase, was added during the procedure.

g) Northern blot showing similar RNA transcript profiles of L1HS-consensus reporter (Con) and RRACH mutant reporter (Mut).

For all qPCRs, data show average +/- SD. n.s, not significant; *, $p< 0.05$; **, $p< 0.01$; ***, $p< 0.001$, two-tailed Student's t- test.
