# Supplementary material for: RNA m6A modification orchestrates a LINE-1–host interaction that facilitates retrotransposition and contributes to long gene vulnerability
Source: Cell Res. 2021 Jun 9;31(8):861–85. doi: 10.1038/s41422-021-00515-8 (PMC8324889; doi:10.1038/s41422-021-00515-8)
Supplement: Supplementary file 10 — Supplementary Fig 10 [file 41422_2021_515_MOESM10_ESM.pdf]

Supplementary information, Fig. S10

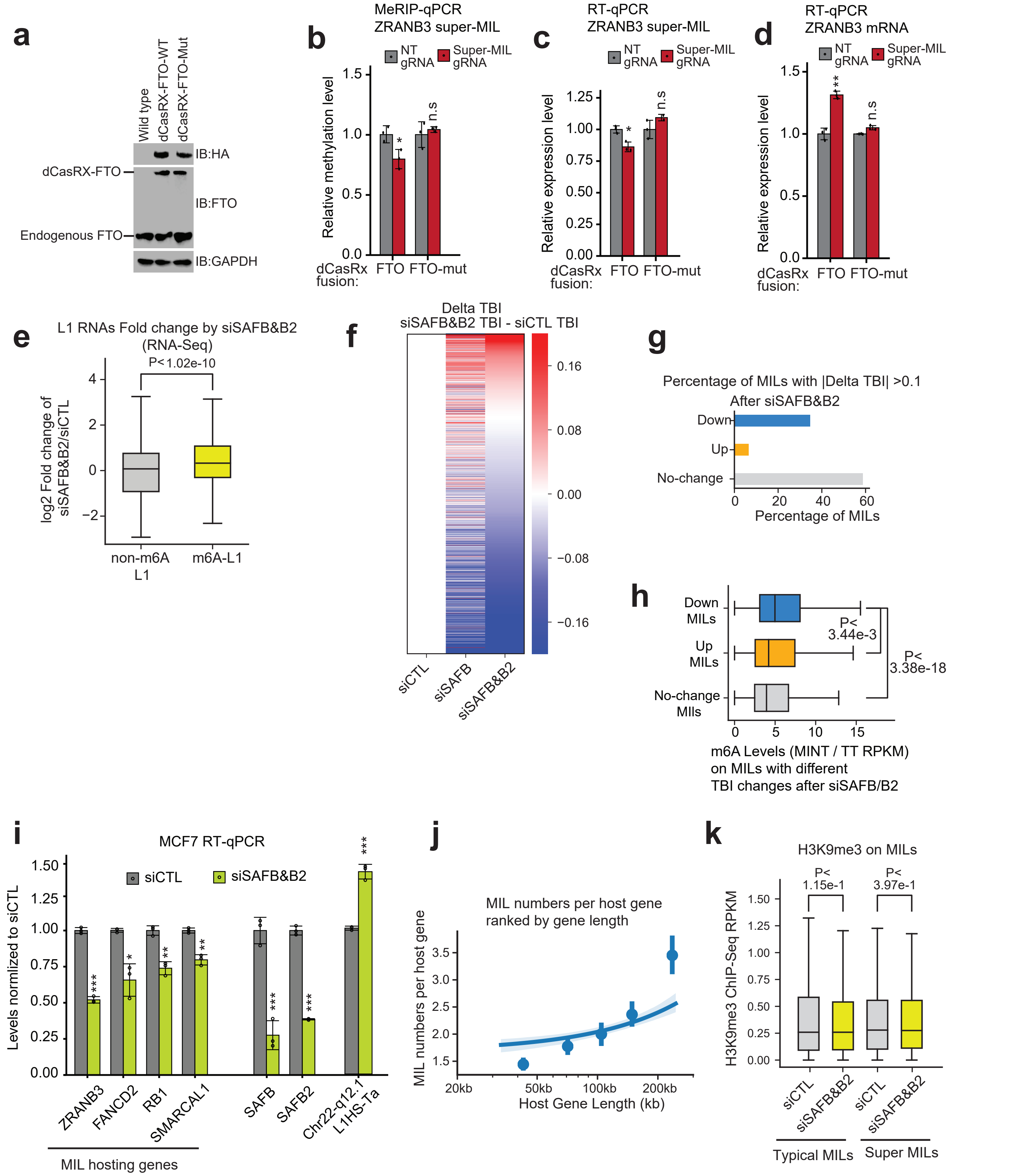

Supplementary information, Fig. S10 | The transcription blocking effect of MILs and Super-MILs is m^6A dependent; SAFB/B2 depletion impacts MILs and host genes without affecting H3K9me3

a) Western blots showing the expression of dCasRX-FTO and dCasRX-FTO-mut (enzymatically-dead mutant FTO). The fusion protein has a C terminus HA tag allowing immunoblotting by HA antibody.

b-d) Recruitment of dCasRX-FTO but not dCasRX-FTO-mut to the Super-MIL in ZRANB3 by specific gRNA reduced the m^6A level of the Super-MIL **(b)**, decreased the RNA level of the Super-MIL **(c)** and increased the host gene ZRANB3 mRNA expression **(d)**. NT gRNA: non-targeting guide RNA; Super-MIL gRNA: guide RNA targeting the ZRANB3 Super-MIL.

e) Boxplot of RNA-Seq data showing that m^6A intronic L1 was more induced versus non-m^6A intronic L1 after SAFB and SAFB2 co-depletion (associated with Fig.6). P-values were calculated with Mann-Whitney U tests.

f-g) Heatmap (**f**) and bar plot (**g**) showing the changes of TBIs for all MILs after SAFB and SAFB2 co-depletion (TBIs calculated based on TT-Seq).

h) Box plot showing that the TBI down MILs (same group of MIL in panel **g**) after SAFB/B2 co-depletion have the highest m^6A level. P-values were calculated with Mann-Whitney U tests.

i) RT-qPCR showing that co-depletion of SAFB and SAFB2 reduced the expression of MIL-hosting DDR genes in MCF7 cells, but increased the L1HS-Ta in Chr22-q12.1 (associated with Fig. 6).

j) Similar to Fig.6i, this is a regression plot showing the relationship between gene length and the number of MIL it hosts.

k) H3K9me3 ChIP-Seq results showing the SAFB and SAFB2 co-depletion did not change H3K9me3 level on typical MILs or Super-MILs. P-values were calculated with Mann-Whitney U tests.

For all qPCRs, data show average +/- SD. n.s, not significant; *, $p< 0.05$; **, $p< 0.01$; ***, $p< 0.001$, two-tailed Student's t-test.
