# Supplementary material for: RNA m6A modification orchestrates a LINE-1–host interaction that facilitates retrotransposition and contributes to long gene vulnerability
Source: Cell Res. 2021 Jun 9;31(8):861–85. doi: 10.1038/s41422-021-00515-8 (PMC8324889; doi:10.1038/s41422-021-00515-8)
Supplement: Supplementary file 11 — Supplementary Fig 11 [file 41422_2021_515_MOESM11_ESM.pdf]

Supplementary information, Fig. S11

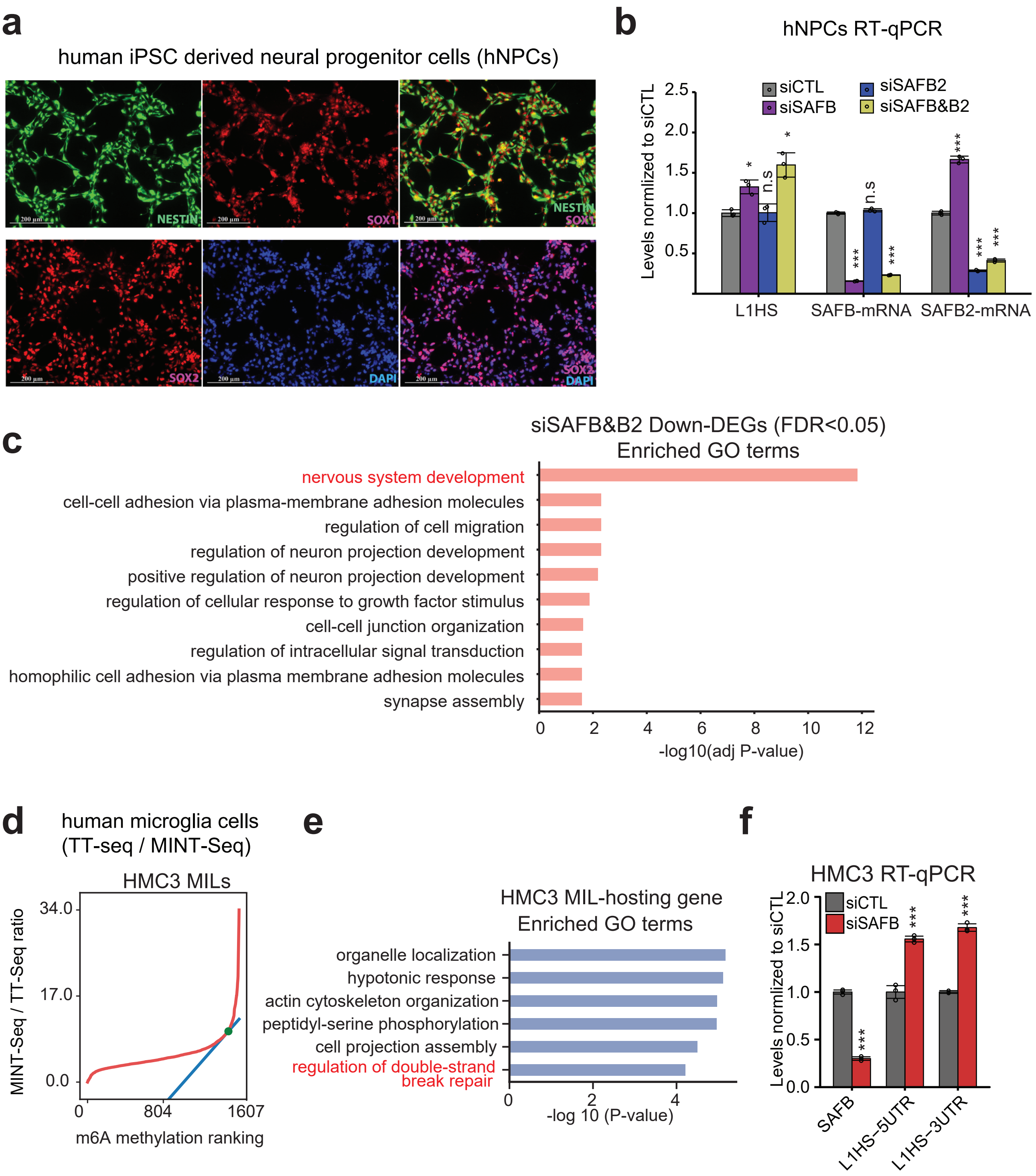

Supplementary information, Fig. S11 | Expression and function of MILs/SAFB in human iPSC derived neural progenitor cells (NPCs) and a human microglia cell line HMC3.

a) Immunofluorescent staining of human iPSC-derived neural progenitor cells (hNPCs) using antibodies targeting Nestin, SOX1, SOX2 together with DAPI staining. Scale bars are included, 200um. Proteins targeted by each staining were labeled at the lower right corners of each panel, together with DAPI.

b) RT-pPCR results showing the expression changes of L1HS, SAFB and SAFB2 after knocking down SAFB or SAFB2 separately or together in hNPCs.

c) Top GO terms of genes down-regulated after dual knockdown of SAFB and SAFB2 (i.e., Down-DEG, EdgeR, FDR < 0.05) in hNPC cells. Enrichment was calculated based on expressed genes in hNPCs (RNA-Seq FPKM > 0.1).

d) Similar to Fig. 1e**,** this is a ranked plot showing relative m^6A levels (MINT-Seq FPKM/ TT-Seq FPKM) across MILs in the HMC3 cell line that represents human embryonic microglia cells. Super-MILs were identified by using the slope of the distribution curve (see Materials and methods).

e) Top terms of gene functional enrichment analysis for genes hosting MILs in the HMC3 cells, using the transcribed genes in this cell line (HMC3 TT-Seq FPKM > 0.1) as the background gene group.

f) RT-qPCR results showing that knockdown of SAFB in HMC3 cells increased L1HS expression, revealed by two different primer sets targeting the 5'UTR and 3'UTR of L1HS RNAs, respectively. For RT-qPCRs in panels **b** and **f**, data show mean +/- SD. *, p< 0.05, **, p< 0.01; ***, p< 0.001, Student's t-test.
