# Supplementary material for: RNA m6A modification orchestrates a LINE-1–host interaction that facilitates retrotransposition and contributes to long gene vulnerability
Source: Cell Res. 2021 Jun 9;31(8):861–85. doi: 10.1038/s41422-021-00515-8 (PMC8324889; doi:10.1038/s41422-021-00515-8)
Supplement: Supplementary file 12 — Supplementary Fig 12 [file 41422_2021_515_MOESM12_ESM.pdf]

Supplementary information, Fig. S12

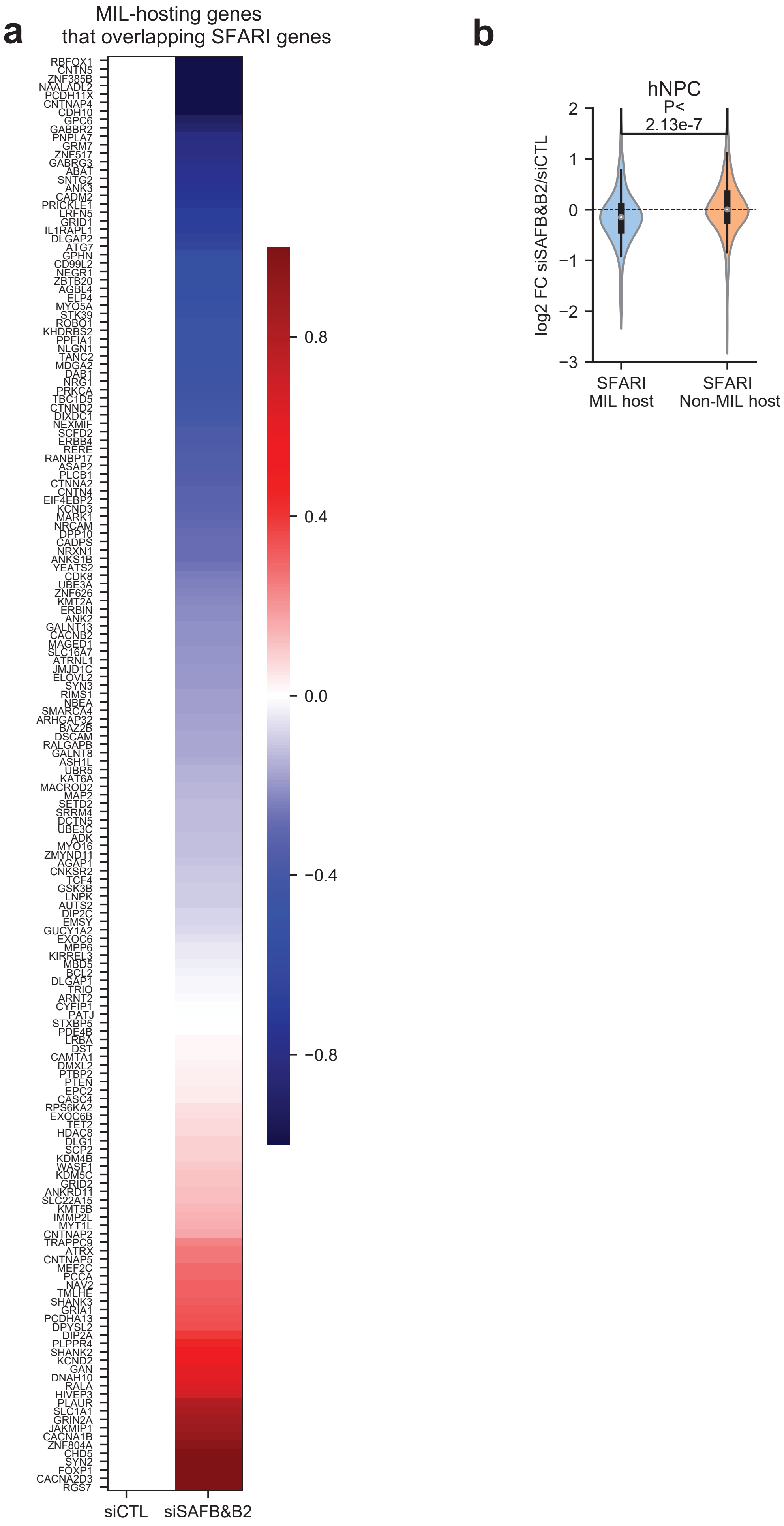

Supplementary information, Fig. S12 | MIL-hosting, autism-associated neuronal/synaptic genes and their vulnerability to deregulation by MILs after SAFB&B2 knockdown.

a) A heat map showing the log2 fold changes of the list of SFARI genes that host MILs after co-depleting SAFB and SAFB2 in hNPCs. Many important neuronal/synaptic genes discussed in the main manuscript were deregulated.

b) A violin plot showing the expression change of SFARI genes with or without MILs in their introns after co-depletion of SAFB and SAFB2 in hNPCs. P-values were calculated with Mann-Whitney U tests.
